# Genome sequencing reveals metabolic and cellular interdependence in an amoeba-kinetoplastid symbiosis

**DOI:** 10.1038/s41598-017-11866-x

**Published:** 2017-09-15

**Authors:** Goro Tanifuji, Ugo Cenci, Daniel Moog, Samuel Dean, Takuro Nakayama, Vojtěch David, Ivan Fiala, Bruce A. Curtis, Shannon J. Sibbald, Naoko T. Onodera, Morgan Colp, Pavel Flegontov, Jessica Johnson-MacKinnon, Michael McPhee, Yuji Inagaki, Tetsuo Hashimoto, Steven Kelly, Keith Gull, Julius Lukeš, John M. Archibald

**Affiliations:** 1 Department of Biochemistry & Molecular Biology, Dalhousie University, Halifax, Nova Scotia Canada; 2 Centre for Comparative Genomics and Evolutionary Bioinformatics, Dalhousie University, Halifax, Nova Scotia Canada; 3 Sir William Dunn School of Pathology, University of Oxford, Oxford, United Kingdom; 4 Center for Computational Sciences, University of Tsukuba, Tsukuba, Japan; 5 Institute of Parasitology, Biology Centre, Czech Academy of Sciences, České Budějovice, Czech Republic; 6 Life Science Research Centre, Faculty of Science, University of Ostrava, Ostrava, Czech Republic; 7 Graduate School of Life and Environmental Sciences, University of Tsukuba, Tsukuba, Japan; 8 Department of Plant Sciences, University of Oxford, Oxford, United Kingdom; 9 Faculty of Sciences, University of South Bohemia, České Budějovice, Czech Republic; 10 Canadian Institute for Advanced Research, Program in Integrated Microbial Biodiversity, Toronto, Canada; 11 Present Address: Department of Zoology, National Museum of Nature and Science, Tsukuba, Japan; 12 Present Address: Laboratory for Cell Biology, Philipps University, Marburg, Germany; 13 Present Address: Graduate School of Life Sciences, Tohoku University, Tohoku, Japan; 14 Present Address: Institute for Marine and Antarctic Sciences, University of Tasmania, Launceston, Australia; 15 Present Address: National Institute of Advanced Industrial Science and Technology, Tsukuba, Japan

## Abstract

Endosymbiotic relationships between eukaryotic and prokaryotic cells are common in nature. Endosymbioses between two eukaryotes are also known; cyanobacterium-derived plastids have spread horizontally when one eukaryote assimilated another. A unique instance of a non-photosynthetic, eukaryotic endosymbiont involves members of the genus *Paramoeba*, amoebozoans that infect marine animals such as farmed fish and sea urchins. *Paramoeba* species harbor endosymbionts belonging to the Kinetoplastea, a diverse group of flagellate protists including some that cause devastating diseases. To elucidate the nature of this eukaryote-eukaryote association, we sequenced the genomes and transcriptomes of *Paramoeba pemaquidensis* and its endosymbiont *Perkinsela* sp. The endosymbiont nuclear genome is ~9.5 Mbp in size, the smallest of a kinetoplastid thus far discovered. Genomic analyses show that *Perkinsela* sp. has lost the ability to make a flagellum but retains hallmark features of kinetoplastid biology, including polycistronic transcription, *trans*-splicing, and a glycosome-like organelle. Mosaic biochemical pathways suggest extensive ‘cross-talk’ between the two organisms, and electron microscopy shows that the endosymbiont ingests amoeba cytoplasm, a novel form of endosymbiont-host communication. Our data reveal the cell biological and biochemical basis of the obligate relationship between *Perkinsela* sp. and its amoeba host, and provide a foundation for understanding pathogenicity determinants in economically important *Paramoeba*.

## Introduction

The nucleus-associated ‘parasome’ (or *Nebenkörper*) of *Paramoeba* species has puzzled biologists for more than a century. Originally thought to be a ‘secondary’ or ‘parasitic’ nucleus, the parasome of *Paramoeba* Schaudinn, 1896, was in the 1970s proposed to be a distinct organism^1–3^. Ultrastructural data led Hollande^4^ to posit that the parasome was a kinetoplastid protozoan, a taxonomic assignment subsequently confirmed by ribosomal RNA (rRNA) gene sequencing^5^.

The kinetoplastids are named by virtue of their shared possession of a prominent disk-shaped mass of DNA—the ‘kinetoplast’—inside their mitochondrion^6, 7^. The best studied kinetoplastids include the parasitic flagellates *Trypanosoma cruzi* and *Leishmania* spp., which have the capacity to invade cells of vertebrates and are notorious in causing mass mortality in humans and other animals^8, 9^. Other kinetoplastid parasites include the fish pathogens *Cryptobia* and *Ichthyobodo*, the latter to which the endosymbionts of *Paramoeba* species appear closely related^10–13^. Kinetoplastids are also known for their unusual biochemical and molecular features, including mitochondrial RNA editing, mRNA *trans*-splicing, use of modified nucleotides, and the presence of genes in polycistronic arrays^6, 8, 9, 14^.

While molecular data suggest an ancient co-evolutionary relationship between *Paramoeba*/*Neoparamoeba* hosts and their *Perkinsela* sp. endosymbionts^15–17^, precisely when, how, and why the latter came to reside within the former is a long-standing mystery. Because the endosymbiont is non-photosynthetic—and thus unlike the algae involved in the spread of plastids (chloroplasts) by ‘secondary’ endosymbiosis^18^—it provides no obvious energetic benefit to its amoeba host.

**Figure 1:**
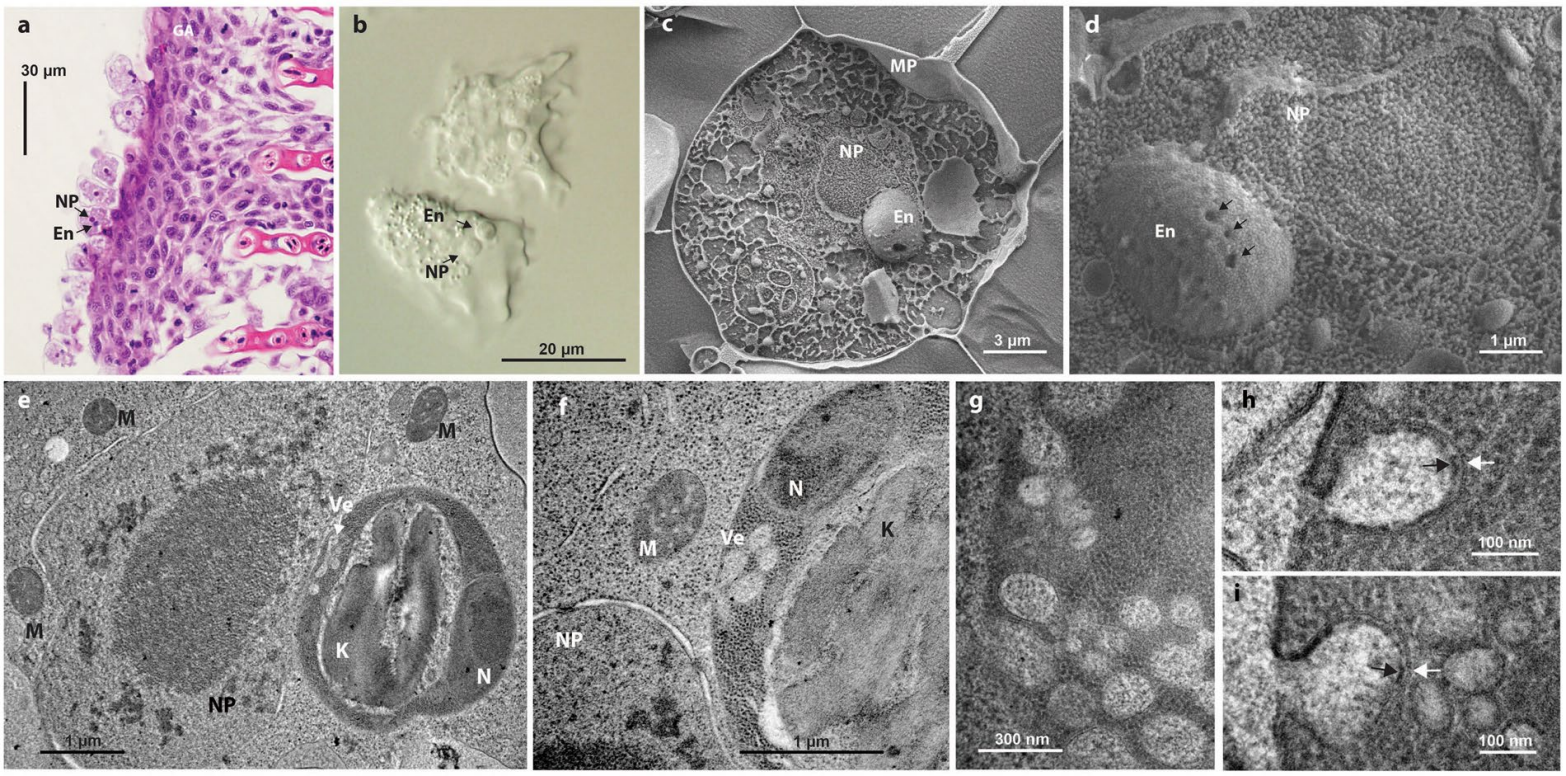
*Paramoeba* and its kinetoplastid endosymbiont *Perkinsela*. (**a**) *Paramoeba* sp. cells stained with haematoxylin and eosin in histological sections of gill tissue of *Salmo salar* (NP = nucleus of the host amoeba; En = *Perkinsela* sp. endosymbiont). (**b**) Trophozoites of *P*. *pemaquidensis* in hanging drop preparations under Nomarski differential interference contrast microscopy. (**c**) High-pressure freezing scanning electron microscopy (SEM) of a *P*. *pemaquidensis* cell with prominent endosymbiont (MP = plasma membrane of *P*. *pemaquidensis*). (**d**) SEM of the host amoeba nucleus and associated endosymbiont with surface invaginations (arrows). (**e**–**i)**. Transmission electron microscopy (TEM) of *P*. *pemaquidensis* and *Perkinsela* sp. (**e** and **f**) TEMs showing close association of the *P*. *pemaquidensis* nucleus (NP) and the endosymbiont *Perkinsela* sp., the kinetoplast (K) of the endosymbiont, the endosymbiont nucleus (N), vesicles within the endosymbiont cytoplasm (Ve), and mitochondria (M) within *P*. *pemaquidensis*. (**g**–**i**) TEMs showing ultrastructure of plasma membrane-associated putative endocytotic vesicles within the cytoplasm of *Perkinsela* sp. White arrows indicate the vesicle membrane, black arrows highlight glycoprotein-rich material on the inner surface of the vesicle, which is continuous with the outer surface of the plasma membrane.

We have used genomics, transcriptomics, and electron microscopy to explore the biology of *Paramoeba pemaquidensis* CCAP 1560/4 (a fish gill-associated species closely related to *Neoparamoeba perurans*, the causative agent of amoebic gill disease^19–22^) and the *Perkinsela* sp. living in its cytoplasm (Fig. 1). Our results show that the metabolisms of the two organisms are interwoven—this explains why their relationship is obligate. Although its gene repertoire is substantially reduced, *Perkinsela* sp. retains kinetoplastid-specific biochemical pathways that could be exploited in the prevention and treatment of diseases caused by the amoeba in which it resides.

## Results and Discussion

### Endosymbiont and host nuclear genome sequencing

The *P*. *pemaquidensis* (host) and *Perkinsela* sp. (endosymbiont) nuclear genomes and transcriptomes were sequenced using Illumina and 454 technologies. All genomic data were derived from the sequencing of a DNA fraction isolated from a Hoechst dye-cesium chloride density gradient. The fraction was enriched in endosymbiont nuclear DNA but also contained host nuclear DNA and mitochondrial DNA from both organisms. A comprehensive set of bioinformatic analyses were performed in order to assign contigs to endosymbiont or host (see Methods). Of particular importance was comparison of genomic and transcriptomic data; we analyzed two different RNA-seq datasets, one of which was generated from a library amplified using an endosymbiont-specific primer corresponding to the spliced leader (SL) sequence (this served to selectively amplify transcripts derived from the kinetoplastid endosymbiont).

From a set of 15,623 genomic scaffolds, 693 scaffolds (all manually curated) were determined to be from the *Perkinsela* sp. nuclear genome. The N50 for these scaffolds was 57,461 base pairs (bp), with an average Illumina sequence depth of ~550X and a G + C content of 47.1% (Table S1.1). The total genome size was estimated to be ~9.5 Mbp. 5,252 protein-coding genes were predicted, with a mean intergenic distance of ~515 bp. 332 of a set of 458 ‘core eukaryotic genes’ (72%) were found in *Perkinsela* sp. using webMGA^23^.

9,331 scaffolds were derived from the *P*. *pemaquidensis* nuclear genome (~120X read depth, N50 = 8,161 bp) (Table S1.1). G + C content was 43.3%. Considering 43.7 Mbp of genomic scaffolds and transcriptomic data, 11,648 protein-coding genes were predicted. Unlike *Perkinsela* sp., whose nuclear genes are apparently intron-free, spliceosomal introns were abundant in the *P*. *pemaquidensis* genome. 40,539 introns were predicted, ~75% of which are 50–150 bp in length (data not shown). The number of introns per gene ranged from zero (~3,500 genes) to > 15 (~500 genes). In terms of ‘completeness’, an analysis of the host-assigned transcriptome dataset identified 378/458 (82%) and 363/458 (79%) core genes using CEGMA and webMGA, respectively. These numbers fall within the range of those obtained in analyses of other sequenced amoebozoan genomes (e.g., using CEGMA, *Dictyostelium* = 422/458 (92%) and *Entamoeba* = 255/458 (56%)).

A single scaffold was found to correspond to the host mitochondrial genome, and three scaffolds were clearly derived from the fragmented, recombination-prone, endosymbiont mitochondrial genome, as described by David *et al*.^24^ (Supplementary Note 1.5). 5,595 scaffolds were ultimately discarded as being of bacterial origin.

### Biology of a kinetoplastid endosymbiont

**Figure 2:**
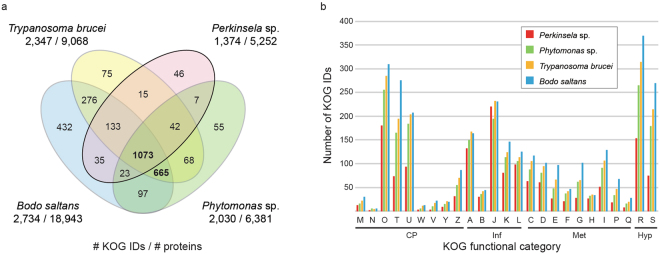
Functional diversity of proteins in *Perkinsela* sp. compared to other kinetoplastids. **(a)** Venn diagram shows overlap in the number of proteins assigned a KOG ID encoded in the nuclear genome of *Perkinsela* sp. CCMP1560/4, the free-living flagellate *Bodo saltans* ^25^, the human pathogen *Trypanosoma brucei* ^27^, and the plant pathogen *Phytomonas* sp.^26^. Functions were assigned based on the euKaryotic Orthologous Groups (KOG) database^29^. The total number of proteins predicted from the nuclear genome of each organism is also shown. **(b)** Histogram showing the unique numbers of KOG IDs found in *Perkinsela* sp., *Phytomonas* sp., *T*. *brucei* and *B*. *saltans*. KOG categories are as follows: A, RNA processing and modification; B, chromatin structure and dynamics; C, energy production and conversion; D, cell cycle control, cell division and chromosome partitioning; E, amino acid transport and metabolism; F, nucleotide transport and metabolism; G, carbohydrate transport and metabolism; H, coenzyme transport and metabolism; I, lipid transport and metabolism; J, translation, ribosomal structure and biogenesis; K, transcription; L, replication, recombination and repair; M, cell wall, membrane or envelope biogenesis; N, cell motility; O, post-translational modification, protein turnover, chaperones; P, inorganic ion transport and metabolism; Q, secondary metabolites biosynthesis, transport and catabolism; R, general function prediction only; S, function unknown; T, signal transduction; U, intracellular trafficking, secretion and vesicular transport; V, defence mechanisms; W, extracellular structures; Y, nuclear structure; Z, cytoskeleton. Higher KOG categories are as follows: CP, cellular processing and signalling; Hyp, poorly characterized; Inf, information storage and processing; Met, metabolism.

At ~9.5 Mbp in size and with < 5,500 predicted protein-coding genes, the *Perkinsela* sp. nuclear genome is substantially smaller than that of the free-living kinetoplastid flagellate *Bodo saltans* ^25^, the plant pathogen *Phytomonas* sp.^26^, and the human parasites *Trypanosoma brucei* ^27^ and *Leishmania major* ^28^ (Table 1). An analysis of proteins with KOG-based functional predictions (euKaryotic Orthologous Groups^29^) revealed > 600 KOG identifiers present in *B*. *saltans*, *T*. *brucei*, and *Phytomonas* sp. but absent in *Perkinsela* sp. (Fig. 2a; refs 25–27). The size and functional diversity of the *Perkinsela* sp. proteome is thus substantially reduced (Fig. 2a and b), presumably as a consequence of adaptation to intracellular life. Almost 14% of its proteome (721 of 5,252 proteins) appears to service its giant mitochondrion (including its mass of mitochondrial DNA, the kinetoplast) (Supplementary Note 1.7), which occupies the majority of the cell’s volume (Fig. 1e) and whose transcripts have been shown to undergo extensive and error-prone U-insertion/deletion RNA editing^24^.

**Table 1:** Features of the *Perkinsela* sp. nuclear genome and those of select kintetoplastids.

	*Perkinsela* sp.^a^	*Bodo saltans* ^b^	*Phytomonas* sp.^c^	*Trypanosoma brucei* ^d^	*Leishmania major* ^e^
Genome size (Mbp)	9.5	39.9	17.8	26.1	32.8
G + C (%)	47.1	46.6	48.0	46.4	59.7
Protein-coding genes	5,252	18,943	6,381	9,068	8,272
Mean intergenic distance (bp)	515.4	462.9	1,140	1,279	2,045
*Trans*-splicing?	yes	yes	yes	yes	yes

^a^Strain CCAP1560/4; This study.

^b^Strain Konstanz; numbers shown correspond to Jackson *et al*.^25^. We analyzed 18,963 protein-coding genes taken from http://www.sanger.ac.uk/resources/downloads/protozoa/bodo-saltans.html

^c^Strain EM^26^.

^d^Strain TREU927^27^.

^e^Strain Friedlin^28^.

The *Perkinsela* sp. genome is compact (mean intergenic distance = 515 bp); it exhibits strand polarity as observed in trypanosomatids^9, 30^ and *B*. *saltans* ^25^ (Supplementary Fig. S1.1), and contains numerous mobile genetic elements similar to those in other *Trypanosoma* species (most notably *ingi*-type non-LTR retrotransposons; Supplementary Note 1.2). SL-mediated *trans*-splicing, a hallmark feature of kinetoplastids, is readily apparent from RNA-seq data and RT-PCR amplifications, but is highly unusual. 5′ and 3′ untranslated regions are extremely short (both average < 100 nucleotides in length) and splice acceptor site usage deviates significantly from the canonical AG dinucleotide acceptor used 99% of the time in other eukaryotes (diplonemids, which are related to kinetoplastids, appear to be another interesting exception^31^). In *Perkinsela* sp., AG is used only 27% of the time, with TG and AT serving as acceptors 26% and 15% of the time, respectively. Only a single intact SL RNA gene was identified in *Perkinsela* sp.; the genome is littered with hundreds of SL RNA gene fragments, some of which are associated with retrotransposons (Supplementary Note S1.3). Canonical *cis* introns were not detected, and while genes for some spliceosomal components were found (e.g., the U5-specific PRP8 protein and various helicases), the *Perkinsela* sp. spliceosome is predicted to be highly reduced (evolutionarily conserved splicing proteins not detected include SMN, SmD3, SmE, SmF, Sm16.5k, SSm4, LSm2, LSm4, LSm7, LSm8, RBP14A, U1-24K, U1-C and U5-40K).

Important aspects of the biology of *Perkinsela* sp. can be inferred by comparing its gene complement to those of more intensively studied kinetoplastids, such as *T*. *brucei*. The flagellum (and associated flagellar pocket) of trypanosomatids is an important organelle, not only for motility but mediating host-parasite interactions and cellular morphogenesis^32–35^. Strikingly, we found that homologs of nearly all of the genes associated with building and maintaining a flagellum are missing from *Perkinsela* sp. (Supplementary Note 2.1). These include homologs of proteins found in the experimentally derived flagellum and flagellum transition zone proteomes of *T*. *brucei* (Supplementary Table S2.1.1)^36, 37^, as well as intraflagellar transport proteins, dynein associated genes, and genes that have been shown to be present only in ciliated organisms^38^. Furthermore, *Perkinsela* sp. has lost the tubulins associated specifically with flagellum function (i.e., delta, epsilon and zeta tubulins), its alpha tubulin has a lysine substitution that is predicted to impact microtubule dynamics, and beta tubulin is missing two motifs required for dynein arm attachment and specification of the central pair. *Perkinsela* sp. is thus unique amongst all kinetoplastid species studied to date: it appears to have lost the basal body, flagellum, and associated membranous and cytoskeletal structures that were presumably present in its free-living ancestors, a conclusion which is in agreement with their absence in microscopy data.

Another hallmark feature of trypanosomatids is the glycosome, a single membrane-bound, peroxisome-like organelle in which glycolytic reactions take place^39^. We found evidence for a glycosome-like organelle in the residual cytoplasm of *Perkinsela* sp. (Supplementary Note 2.5). This includes genes for Pex5 and Pex7 (the cytoplasmic receptors for peroxisomal targeting signal (PTS) 1- and 2-mediated import, respectively), various Pex family membrane proteins, and glycosome/peroxisome division proteins (Supplementary Table S2.2.1 and S2.5.2). Putative peroxisomal targeting signals (PTS 1 or 2) were also found on the first seven glycolytic enzymes encoded in the *Perkinsela* sp. genome (from hexokinase to phosphoglukokinase, with the exception of phosphoglucose isomerase, which has an ambiguous PTS signal; Supplementary Fig. S2.5.1, Supplementary Table S2.5.2). The biochemical processes taking place in the putative glycosome/peroxisome of *Perkinsela* sp. are diverse—beyond glycolysis, these include amino acid, nucleotide, and sterol/isoprenoid metabolism. This has potential implications for the biology of both endosymbiont and host.

### Host-endosymbiont interactions

*P*. *pemaquidensis* and *Perkinsela* sp. have never been successfully cultured separately (ref. 5 and references therein). Indeed, Page noted that “the *Nebenkörper*… [*Perkinsela* sp.] was always so close to the [amoeba] cell nucleus and followed its movements so faithfully that a physical connection between the 2 must be assumed…”^40^. We attempted to discern the underlying reason(s) for the obligate relationship between the two organisms. Endosymbiotic gene transfer (EGT), a well-known factor in the transition from endosymbiont to organelle^41^, is one possibility. We used a phylogenomics pipeline to search the >11,000 genes in the *P*. *pemaquidensis* nuclear genome for genes of kinetoplastid ancestry. Out of 3,846 protein alignments for which interpretable phylogenies could be reconstructed, only 8 genes likely derived from the endosymbiont were identified in the amoeba (Supplemental Note S1.6, Supplemental Fig. S1.6.1a–h). The role of EGT in forging this unusual symbiosis thus appears to have been minimal.

**Figure 3:**
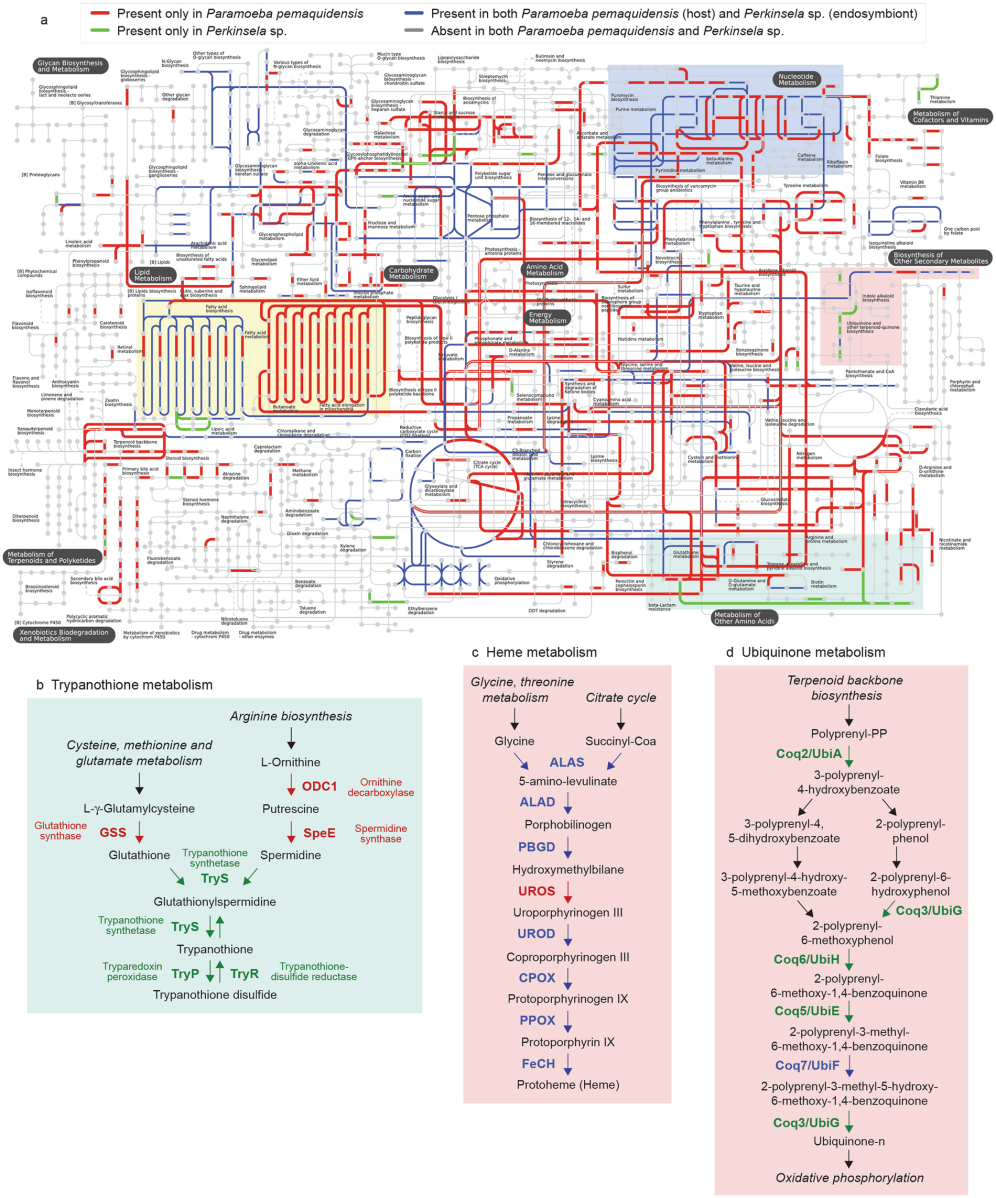
Metabolic maps for *Paramoeba pemaquidensis* (host) and *Perkinsela* sp. (endosymbiont). (**a**) Global metabolic maps. Nodes represent metabolic compounds and lines represent enzyme-catalyzed biochemical reactions. Metabolic maps were based on KEGG annotations and generated using the interactive Pathways Explorer (iPath 2.0). Colored lines indicate enzymes/reactions predicted to be present in one or both organisms. **(b**–**d)** Close-ups of trypanothione, heme and ubiquinone metabolism, respectively. Color-coding corresponds to part a.

We next considered the possibility that the host and endosymbiont metabolisms are functionally intertwined. A global comparison of the KEGG-inferred metabolic capacities of both organisms supports this hypothesis (Fig. 3a). For example, *Perkinsela* sp. appears capable of making trypanothione—an antioxidant unique to kinetoplastids^42^—with its own trypanothione synthase (TryS), but only using glutathione and spermidine synthesized by enzymes encoded by its host (Fig. 3b and Supplementary Fig. S3.1.1a). Genes for 7 of 8 heme biosynthetic enzymes are found in *Perkinsela* sp., with a gene for the ‘missing’ enzyme (uroporphyrinogen-III synthase, UROS) located in the amoeba genome^43^ (Fig. 3c); and genes for the synthesis of mitochondrial ubiquinone are exclusively found in *Perkinsela* sp., with the exception of the ubiquinone biosynthesis monooxygenase (Coq7) gene, a version of which resides in both nuclear genomes (Fig. 3d and Supplementary Fig. S3.1.1b; the ubiquinone biosynthetic pathway is otherwise conserved in amoebozoans). Conversely, arginine and proline metabolic enzymes are primarily encoded by the *P*. *pemaquidensis* genome (Fig. 3a and Supplementary Fig. S3.1.1c), as are fatty acid degradation enzymes (Fig. 3a and Supplementary Fig. S3.1.1d), while purine metabolism is a complex mixture of proteins encoded by both genomes or one or the other (Fig. 3a and Supplementary Fig. S3.1.1e).

**Figure 4:**
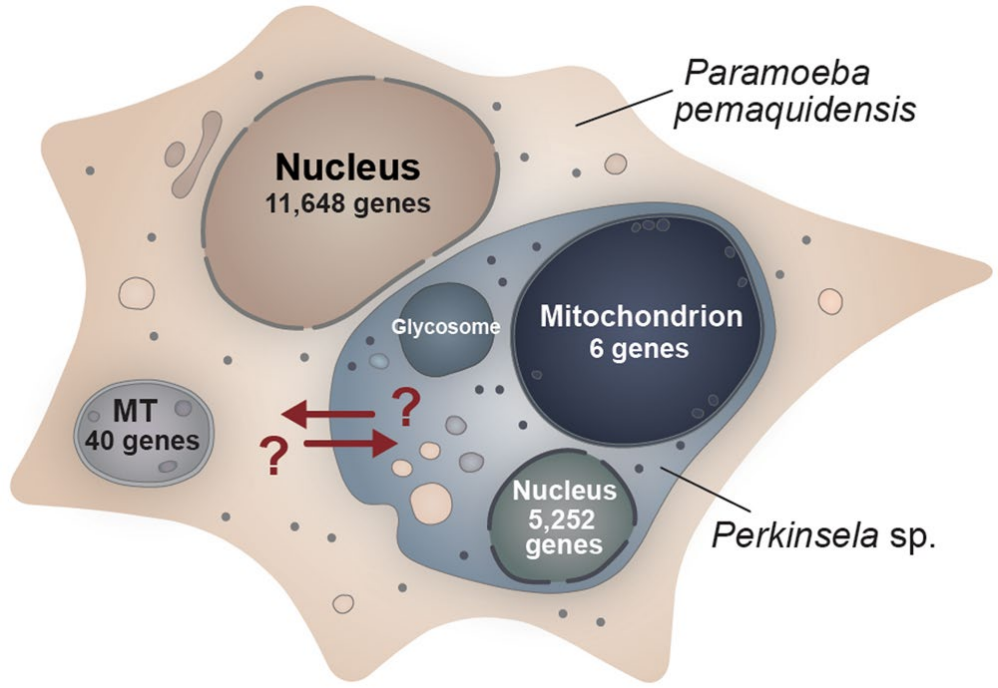
Schematic diagram of *Paramoeba pemaquidensis* and its endosymbiont *Perkinsela* sp. The number of protein-coding genes in each genome is shown. Arrows show possible host-endosymbiont exchange of metabolites via endocytosis and exocytosis by *Perkinsela* sp. Abbreviation: MT, mitochondrion.

How might *Perkinsela* sp. and *P*. *pemaquidensis* ‘communicate’? Using TEM and freeze-fracture cryo-SEM, we discovered invaginations on the surface of the endosymbiont, which appears bound by a single membrane (Fig. 1h,i, Supplementary Fig. S2.6.1). Intracellular vesicles with electron densities similar to the *P*. *pemaquidensis* cytoplasm were also apparent, like those seen in an early study of *P*. *perniciosa* ^2^. These observations suggest that *Perkinsela* sp. carries out endocytosis (although with no flagellar pocket, the site of endocytosis in trypanosomes^32^, the process is presumably not restricted to any particular region of the plasma membrane). This idea is further supported by genomic data. The *Perkinsela* sp. genome encodes a varied set of proteins involved in clathrin-mediated endocytosis, exocytosis, and vesicular trafficking (e.g., clathrin, dynamin, SNARE proteins, and a set of Rab proteins) (Supplementary Note 2.6), as well as 66 putative membrane transporters with diverse substrate specificities (Supplementary Note 2.7). Although their intracellular destination(s) is unknown, we speculate that the endocytic vesicles of *Perkinsela* sp. serve to internalize amoeba-derived metabolites (and, possibly, enzymes), thereby allowing the contents of the amoeba cytoplasm to feed directly into the biochemical pathways of the endosymbiont (Supplementary Note 3.14). In the opposite direction, *Perkinsela* sp. may secrete key proteins and metabolites to its plasma membrane or out into the cytoplasm of its host (Fig. 4). These are hypotheses that can be tested experimentally.

Despite its evolutionary transformation from a free-living flagellate to an aflagellate, obligate endosymbiont, several factors speak to the semi-autonomous nature of *Perkinsela* sp. Most unexpected is our discovery of genes (with RNA-seq support) for sexual recombination. Homologs of DMC1 (Tb927.9.9620), HOP1 (Tb927.10.5490), Spo11 (Tb927.5.3760) and MND1 (Tb927.11.5670) have been identified in *T*. *brucei* and are expressed during the meiotic lifecycle stage of the parasite^44^. The *Perkinsela* sp. genome has clear orthologs of three of these: it possesses DMC1 (a RAD51 homolog that promotes strand exchange), HOP1 (a component of the synaptonemal complex) and Spo11 (which catalyzes meiosis-specific double stranded breaks), but appears to have lost MND1 (known to stabilize heteroduplexes after double stranded break formation) (Supplementary Table S2.1.1). Additionally, *Perkinsela* sp. contains a divergent homolog of HAP2 (Tb927.10.10770), a polytopic protein involved in gamete fusion that is widely conserved across eukaryotes. Collectively, these results suggest that opportunities for meiotic exchange exist between *Perkinsela* sp. cells, perhaps in conjunction with the sexual cycle of *P*. *pemaquidensis* (which also has meiosis genes). *Perkinsela* sp. also has various cell cycle-related genes (e.g., cyclins and MCM proteins) (Supplementary Note 2.8), evidence that it retains at least some control over its own division.

### *Perkinsela* sp. as secondary endosymbiont: practical and conceptual implications

The precise role of *Perkinsela* sp. in the pathogenicity of *Paramoeba* species is unknown. Nevertheless, our demonstration of an intimate metabolic association between endosymbiont and host suggests that *Perkinsela* sp. should not be ignored in future efforts to diagnose, treat and prevent amoebic gill disease and related afflictions in marine animals. The co-evolutionary association between the two organisms is clearly ancient and kinetoplastid-specific metabolic pathways such as trypanothione biosynthesis are potential therapeutic targets—drugs aimed at the endosymbiont could indirectly kill the host.

At the same time, a fuller understanding of the extent to which *Perkinsela* sp. provides essential metabolites to *P*. *pemaquidensis* and vice versa will hopefully shed light on the circumstances that led to this unusual endosymbiosis. In the case of algae, the phenomenon of secondary (i.e., eukaryote-eukaryote) endosymbiosis is typically explained in terms of the host-associated benefits of acquiring a plastid and photosynthesis^18^. However, experimental and theoretical investigations of the recently evolved facultative symbiosis between the ciliate *Paramecium bursaria* and the green alga *Chlorella* sp. paint a more complex picture. In this system, ‘acquired phototrophy’ can manifest itself as parasitism, mutualism or host-driven symbiont exploitation depending on environmental conditions such as light intensity and nutrient availability^45–47^. Given that parasitic kinetoplastids have evolved from free-living species on multiple occasions^7, 11, 48^, it would not be surprising if the initial interactions were deleterious from the amoeba’s perspective. Indeed, the closest known relatives of *Perkinsela* sp. within Kinetoplastea are members of the genus *Ichthyobodo* ^10–12^, which are ectoparasites of fish^13^.

These uncertainties aside, the *Paramoeba* – *Perkinsela* sp. endosymbiosis provides a unique perspective on secondary endosymbiosis. In some ways the metabolic mosaicism we describe is reminiscent of the nutritional symbioses that exist between sap-feeding insects and bacteria, the latter carrying out essential metabolic processes (e.g., vitamin and amino acid biosynthesis) that serve to augment the insect’s nutrient-poor diet^49, 50^. Key challenges for future research include (i) defining the biochemical and cellular channels of communication between host and endosymbiont (Fig. 4), (ii) understanding how and why *Perkinsela* sp. associates with the amoeba nucleus, and (iii) determining the significance of the giant DNA-packed mitochondrion of *Perkinsela* sp. and how its biology might link to the host amoeba.

## Methods

### Cell Culturing, Nucleic Acid Extraction, and Density Gradient Centrifugation

*Paramoeba pemaquidensis* CCAP 1560/4 was grown on marine agar plates for 14 days as described previously^51^. To remove *E*. *coli* food bacteria and concentrate amoeba cells, agar surfaces were washed three times with seawater and the resulting cell suspensions filtered through a 3-μm polycarbonate membrane. Membranes were rinsed twice with seawater and the washes discarded. After the second wash, amoebae were collected from the membranes by gentle pipetting with seawater. For further removal of bacteria, the cell suspension was treated with antibiotics (Sigma antibiotic mix P4083; 100 μL of mix per 5 mL filtered cells). This mixture was spread on an agar plate and incubated overnight. Cells were then collected as before with seawater rinses and membrane filtrations.

Total cellular DNA was extracted and subjected to Hoechst dye-cesium chloride density gradient centrifugation at 40,000 *g* for 67 hours, as described by Lane and Archibald^52^. Three distinct fractions were isolated, purified and eluted in 50 μL of Tris-EDTA buffer. Semi-quantitative PCR was used to assess the genomic origin and purity of each fraction using gene- and genome-specific primers (*Perkinsela* sp. nuclear genome *rpb1* gene, host amoeba nuclear genome *rpb1*, and the *cox1* gene of the host mitochondrial genome) (Supplementary Fig. S4). The second fraction, which was rich in DNA from the endosymbiont (but nevertheless still contained DNA from the amoeba nuclear and mitochondrial genomes), was used for sequencing.

### DNA Sequencing and Assembly

Several sequencing libraries were made from total genomic DNA (fraction 2), including a small-insert (0.5 kbp) paired end library and a large insert (2.5 kbp) mate pair library. Illumina sequencing produced 30 × 2 million reads and 242 × 2 million reads from the small and large insert libraries, respectively. Small insert and mate-paired reads were trimmed to 90 bp and 95 bp, respectively, using FASTX-toolkit (http://hannonlab.cshl.edu/fastx_toolkit/). Trimmed reads with quality scores >20 for at least 75% of their length were retained. After quality control and trimming, 22 × 2 million reads and 220 × 2 million reads were kept from the small- and large-insert libraries, respectively. Six million single reads whose counterpart failed at the quality control stage were also retained. Genome sequencing using Roche GS-FLX 454 technology was also performed, generating ~1 million reads with an average length of 440 bp. Illumina and 454 sequence reads were assembled using Ray (Ray-v2.2.0)^53^. Kmer-genie (kmergenie-1.5533)^54^ was used to predict an optimal kmer size for genome assembly of 49. Contigs were scaffolded using SSPACE (SSPACE-BASIC-2.0_linux-x86_64) (k = 5, a = 0.7, z = 500)^55^, resulting in 15,623 scaffolds.

### Transcriptome Sequencing and Assembly

Two RNA-seq libraries were generated and sequenced for CCAP 1560/4. The first was constructed from total RNA extracted from *P*. *pemaquidensis* cells (and their endosymbionts) using TRIzol reagent and treated with DNase I to remove DNA contamination. This ‘total RNA’ library was prepared using an Illumina TruSeq RNA Sample Preparation kit. The second library involved using an endosymbiont-specific primer corresponding to the spliced leader (SL) sequence for second strand synthesis, the goal being to selectively amplify RNA from the kinetoplastid endosymbiont. This ‘SL’ library was prepared using total RNA extracted with a Qiagen RNeasy Mini kit, followed by mRNA purification using a PolyAT Tract mRNA Isolation kit (Promega). First strand DNA was synthesized with oligo-dT primers using the Qiagen Ominiscript RT Kit. After purification of single stranded cDNA, the second strand was synthesized with a SL-specific primer (5′-AAAATAGTTCAGTTTCTGTACTTAATTG-3′) using Phusion High-Fidelity DNA polymerase (New England BioLabs). After second strand synthesis, the single stranded cDNAs which do not contain a SL (i.e., host amoeba and mitochondrial transcripts) were digested using exonuclease I enzyme. To obtain enough DNA for a sequencing library, cDNAs were amplified by PCR using oligo-dT and SL-specific primers for 5 cycles. A total RNA library was also prepared, using similar techniques, for a different strain of *P*. *pemaquidensis* (ATCC 50172).

Total RNA libraries for CCAP 1560/4 and ATCC 50172 were sequenced on an Illumina HiSeq 2000 at the McGill University and Genome Quebec Innovation Centre. The SL-amplified cDNA library was sequenced on a HiSeq 2000 at GENEWIZ (genewiz.com). Sequencing of the total RNA library of CCAP 1560/4, with 100 bp read lengths, generated 130 × 2 million reads while the SL library yielded 170 × 2 million reads. Reads were assembled using Trinity (trinityrnaseq-r2013-02-25)^56^, yielding 42,335 and 49,770 transcripts from the total RNA and SL RNA libraries, respectively. For ATCC 50172, 263 × 2 million reads were generated, and were assembled into 29,576 transcripts using Trinity.

### Genome Assemblies and Scaffold Curation

Density gradient-purified material from fraction 2 was found to be a mixture of DNA from the nuclear and mitochondrial genomes of the host (*P*. *pemaquidensis*), the nuclear and mitochondrial genomes of the endosymbiont (*Perkinsela* sp.), as well as DNA from contaminating bacteria. Starting with 15,623 genomic scaffolds, numerous bioinformatic analyses were carried out in order to filter out contamination and to produce endosymbiont nuclear and host nuclear genome assemblies.

Our assembly of all sequence data was first compared to the NCBI NT database using BlastN. Scaffolds with clear bacterial hits were removed. After gene models were generated (below), ORFs were compared to the NCBI NR database and tentatively flagged as either eukaryotic or bacterial. The ‘eukaryotic’ hits were further classified as ‘kinetoplastid-related’ (i.e., endosymbiont), ‘amoebozoan-related’ (host) or ‘unassigned but eukaryotic’. These rough designations were not seen as definitive, but rather used to help determine whether a given scaffold was likely to be of endosymbiont, host, or bacterial origin.

Since sequencing was carried out using an endosymbiont genome-enriched DNA fraction, we hypothesized that read coverage depth of the *Perkinsela* sp. genome might differ from that of the host nuclear genome as well as DNA sequenced from contaminating/food bacteria. We thus mapped genomic reads to the total assembly using BWA^57^ and calculated average read depth coverage for each scaffold using an in-house Perl script. We also mapped RNA-seq reads from the two transcriptome libraries, i.e., the total RNA library and the SL-amplified RNA library, to all scaffolds in the total genome assembly. The rationale here was that kinetoplastid endosymbiont-derived transcripts should be present in much higher abundance in the SL-amplified library than in the total RNA library. We attempted to quantify this difference by (i) determining the total number of RNA-seq reads from each library that mapped to each scaffold and (ii) calculating the ratio of RNA-seq reads in the SL RNA library to those in the total RNA library for each scaffold.

In addition to library-specific RNA-seq data, we considered various lines of evidence in our initial assignment of scaffolds to the nuclear genomes of *P*. *pemaquidensis* (host) or *Perkinsela* sp. (endosymbiont). These included (i) the presence of SL RNA genes/gene fragments, (ii) the presence of a kinetoplastid-specific retrotransposon (L1Tc), (iii) the presence/absence of spliceosomal introns, (iv) gene density, and (v) the results of BLAST analyses and published single gene phylogenies^24, 58^. This step involved manual curation of 136 putative endosymbiont nuclear scaffolds, 68 host nuclear scaffolds, 3 mitochondrial scaffolds, and 44 bacterial (i.e., contaminant) scaffolds. Scaffold-specific SL RNA/total RNA ratios were then plotted against sequence read depth and G/C content. While most of the curated host nuclear contigs had SL RNA/total RNA ratios of < 0.5, the initial set of endosymbiont scaffolds had ratios between 10 and 100 (Supplementary Fig. S5). The average G + C content and read depths of these two sets of scaffolds were also somewhat different. We thus considered all unassigned scaffolds with a SL RNA/total RNA ratio of > 2.5, a read depth of > 80x, and a G + C content of > 45% as also possibly being endosymbiont-derived. Genomic scaffolds without any total RNA-seq reads were removed from further consideration. A second, more exhaustive round of manual curation was then performed (including visual inspection of BlastX results), allowing scaffolds of more ambiguous origin to be designated as host, endosymbiont, or bacterial contamination.

### Gene Modeling and Annotation

Gene models were predicted separately for the host and endosymbiont nuclear genome assemblies using AUGUSTUS^59^. ‘Training’ gene sets were obtained by partitioning the total RNA-seq assembly into host- or endosymbiont-specific contigs based on BlastN searches against confidently assigned genomic scaffolds. Parameter training was performed using the WebAUGUSTUS server (http://bioinf.uni-greifswald.de/webaugustus/about.gsp). AUGUSTUS gene predictions were then carried out with the parameters and ‘hints’ generated during training. Special attention was given to the gene modeling parameters for the endosymbiont genome, as it was clear from preliminary RT-PCR and transcriptome mapping experiments that *Perkinsela* sp. nuclear genes lack spliceosomal introns (only *trans* splicing events were identified) and can be transcribed in a polycistronic fashion.

Independent transcriptome-based gene predictions were also carried out for the *Perkinsela* sp. and host nuclear genomes using PASA^60^. In the case of *Perkinsela* sp., this process generated numerous gene models that corresponded to immature polycistronic transcripts that had not yet undergone *trans* splicing. We thus relied on AUGUSTUS as the primary source of gene models for the endosymbiont nuclear genome. Nevertheless, gene models identified by PASA, but missing from the AUGUSTUS predicted gene set, were investigated and added to the *Perkinsela* sp. gene set.

This merged set of gene models was used as an initial training set for a custom iterative training and prediction pipeline using AUGUSTUS. In brief, multiple iterations of prediction and training were conducted until prediction converged on a final set of gene models and no further genes could be detected. Following each prediction round, the orientation of each gene was analyzed and genes whose orientation was inconsistent with its immediate neighboring genes were discarded. Gene models were also predicted using OrthoFinder^61^ and GeneMarkES^62^. Gene models were retained if they satisfied three filtration criteria: (i) the gene model encompassed both start and stop codons, (ii) the gene model did not overlap with any existing AUGUSTUS-derived gene model, and (iii) the orientation of the predicted gene was consistent with the orientation of at least one direct neighboring gene. This iterative approach resulted in the identification of an additional 1,136 putative endosymbiont nuclear genes. After manual curation, these additional gene models were added to those generated using AUGUSTUS and PASA, resulting in a total of 5,252 predicted genes in the nuclear genome of *Perkinsela* sp.

For *P*. *pemaquidensis* (CCAP1560/4), comparison of a test set of 679 mRNA transcripts with strong amoebozoan signatures to our host nuclear genomic assembly and AUGUSTUS gene models revealed that the assembly was incomplete and that not all genes had been predicted (only 589 of 679 transcripts had a match). In order to produce a more complete picture of the coding capacity of the *P*. *pemaquidensis* nuclear genome, we thus augmented our set of AUGUSTUS gene models by considering mRNA transcripts from PASA (unlike *Perkinsela* sp., spliceosomal introns were abundant in *P*. *pemaquidensis* and there was no evidence of polycistronic transcription or SL *trans* splicing). After removal of splicing variants, the remaining transcripts were subjected to BlastN searches against the host and endosymbiont nuclear and mitochondrial genomes. Those with perfect or near perfect matches against the host nuclear assembly were retained if they matched areas not covered by existing gene models. Transcripts matching the other genomes were discarded. Transcripts that did not match any of the genome assemblies were compared to the NR database using BlastX (E value cutoff < 1E-10), and those for which the top five hits were to amoebozoan proteins were retained. We also determined the extent of overlap between the transcriptomes of CCAP 1560/4 and ATCC 50172. Any transcripts present in both datasets that had not already been considered were compared against the NT database to remove contaminants. In total, 20,406 mRNA transcripts were considered to be of host nuclear origin. This set of transcripts was considered for some (but not all) of our downstream analyses, recognizing that on the basis of RNA-seq data alone, mRNA transcripts cannot be definitively assigned to the nuclear genome of *P*. *pemaquidensis* CCAP 1560/4.

Gene models for the host and endosymbiont nuclear genomes were viewed and edited manually with GenomeView^63^ and various in-house Perl scripts. Gene annotations were performed automatically using the webMGA server^23^ to generate KOG, KEGG, PFAM, GO and EC predictions. Amino acid sequences were also used as BlastP queries against the NCBI protein database and an in-house database of kinetoplastid proteins obtained from TriTrypDB^64^. SL addition sites for the endosymbiont genome/transcriptome were identified using SLaP mapper^65^ and loaded into the genome browser.

### Mitochondrial genome assembly and annotation

The *P*. *pemaquidensis* mitochondrial genome was initially assembled into a single scaffold 53,489 bp in length. This scaffold was found to contain an artifactual sequence duplication of 4,967 bp on each end. Manual resolution of the duplicated area resulted in a scaffold of 48,522 bp. Gene annotation was carried out using Artemis version 16.0.0, NCBI BlastN and BlastP (http://blast.ncbi.nlm.nih.gov/Blast.cgi), as well as Uniprot Blast (http://www.uniprot.org/blast/uniprot/B2015111965CZCMIP1J). Transfer RNA (tRNA) genes were predicted with tRNAscan v.1.21 (http://selab.janelia.org/tRNAscan-SE/). The circular mapping genome was visualized using OrganellarGenomeDRAW (http://ogdraw.mpimp-golm.mpg.de). The *Perkinsela* sp. (endosymbiont) mitochondrial genome was sequenced and characterized as described by David *et al*.^24^.

### Phylogenomics

To identify gene transfers between *P*. *pemaquidensis* and *Perkinsela* sp., we implemented a phylogenomics pipeline (Supplementary Fig. S1.6.2) using predicted proteins from both the host and endosymbiont genomes. BlastP searches were used to retrieve a maximum of 1,000 sequences from a comprehensive database comprised of proteins from public genomic and transcriptomic databases (see Supplementary Table S1.6.1), with an E-value cut-off of 1E-15 and a query-target alignment length of 50% or more. After multiple sequence alignment and preliminary phylogenetic analysis using FastTree^66^, TreeTrimmer^67^ was used to reduce OTU redundancy for alignments containing > 100 sequences using the criteria shown in Supplementary Table S1.6.2 (paralogs within the *P*. *pemaquidensis* and *Perkinsela* sp. genomes were retained in each alignment, regardless of how similar they were to one another). Alignments were then repeated, trimAL ver. 1.4^68^ was used to remove ambiguously aligned regions, and phylogenetic trees were inferred using FastTree. The number of proteins for which trees could be constructed (i.e., at least 4 OTUs including the query) was 3,846 for *P*. *pemaquidensis* (out of 11,573 predicted proteins in total at the time of analysis) and 2,633 for the endosymbiont *Perkinsela* sp. (out of 5,252 proteins). Tree topologies were screened in an automated fashion using an in-house Ruby script; trees of interest (i.e., trees in which *P*. *pemaquidensis* or *Perkinsela* sp. proteins branch with one or more kinetoplastid or amoebozoan homologs, respectively) were selected for closer scrutiny. For these initial endosymbiotic gene transfer (EGT) candidates (35 host and 6 endosymbiont proteins), additional homologs (if present) were retrieved from the GenBank NR database using BlastP searches and added to the datasets. After elimination of redundant sequences, multiple alignment and removal of ambiguously aligned sites, trees were inferred from the final curated alignments using RAxML ver. 7.7.9 (with the LG + G + F model^69^) (Supplementary Fig. S1.6.2). Trees were then checked manually, and those with topologies consistent with EGT were flagged for further investigation. Branch support was evaluated by rapid bootstrapping in RAxML (100 replicates), using the same substitution model described above. The flagged datasets were also analyzed using PhyloBayes ver. 3.3f^70^ with the site-heterogeneous mixture CAT model. Two independent Markov chains were run for a total of 10,000 cycles. Bayesian posterior probabilities were calculated by sampling every 10 trees after discarding the first 2,500 trees as ‘burn-in’.

### Subcellular Localization Predictions

Sequence similarity searches for genes encoding mitochondrial-targeted proteins in *Perkinsela* sp. were based on a curated set of mitochondrial proteins taken primarily from proteomic studies of *T*. *brucei* ^71–74^. For each gene ID from *Trypanosoma brucei brucei* TREU 927, orthologous sequences were retrieved from the OrthoMCL database (v. 5) and aligned with MUSCLE (v. 3.8.31)^75^. A few alignments were manually edited as they contained ambiguous characters or artificially long sequences. Alignments were converted to Hidden-Markov model profiles and searched for in a 6 frame-translation of the *Perkinsela* sp. nuclear scaffolds using HMMER (v. 3.1b2)^76^ with an E-value cutoff of 1E-10 and a N^max^ of 10. Non-specific hits with E-values ≥1E-100 were removed if preceded by hits with substantially greater similarity, i.e., an E-value difference of 1E-20 or more. Annotated sequences were retrieved from hit regions and used as queries for reciprocal BlastP (v. 2.2.30) searches. Reciprocal hits were considered valid if the highest scoring hit was present in the initial *Trypanosoma* ID list with an E-value ≤ 1E-5.

To determine whether *Perkinsela* sp. contains a glycosome or peroxisome-like organelle, we carried out genomic and transcriptomic screens using various complementary approaches (summarized in Fig. S6). This included BlastP analysis of the *Perkinsela* sp. proteome using as queries a set of experimentally verified, “high-confidence” glycosomal proteins from the procyclic form of *Trypanosoma brucei* ^77^, as well as potential glycosomal biogenesis components from *Leishmanisa donovani* ^78^. We also (i) carried out BlastP searches using *Saccharomyces cerevisiae* and *Trypanosoma* sp. peroxisome biogenesis and protein import-specific peroxin (Pex) protein sequences retrieved from NCBI; (ii) considered the results of KEGG annotation (KAAS, http://www.genome.jp/tools/kaas/)^79^ of the endosymbiont and host nuclear genomes; and (iii) used an in-house script to perform a peroxisomal targeting signal 1 (PTS1) motif search of *Perkinsela* sp. proteins based on PTS1 signaling information taken from Jamdhade *et al*.^78^ (i.e., the presence of characteristic C-terminal tri-peptides ([ASCGPNYTV][KNRHQDS][LMVAIF]). We also performed BlastP searches of the *Perkinsela* sp. proteome using query proteins known to be localized to other single membrane bound kinetoplastid organelles, including the reservosome^80^ and the acidocalcisome^81^, as well as *T*. *brucei* proteins predicted to be involved in autophagy (pexophagy: degradation of peroxisomes/glycosomes)^82^. Finally, the presence of glycolysis and TCA cycle enzymes, the mevalonate and pentose phosphate pathways, Rab GTPases, and ESCRT components were investigated by BlastP analyses of the *Perkinsela* sp. protein database (queries were obtained from NCBI or KEGG).

For reference, the *P*. *pemaquidensis* nuclear genome was screened for the presence of peroxisome-specific peroxins as well as glycolytic enzymes via BlastP against the host “bestmodel protein” database (11,573 protein sequences) using NCBI-derived *Saccharomyces cerevisiae* and *Acanthamoeba castellanii* sequences as queries. In cases where key enzymes were not detected, more detailed BLAST analyses (tblastN) were performed. For example, host-encoded Pex16 and Pex19 protein components were found using tblastN, not blastP. Similarly, the gene for the key glycolytic enzyme triose-phosphate isomerase (TPI/TIM) was found only after examination of transcriptome data prior to sorting into host- and endosymbiont-derived transcripts.

Blast hits obtained using these approaches were analyzed for their completeness and the presence of conserved functional domains using NCBI blastP and *Conserved Domain Detection* (CDD). The presence of potential N-/C-terminal or internal targeting signals and transmembrane domains (TMDs) was investigated using a variety of online bioinformatic tools. Glycosomal/peroxisomal targeting sequences (PTS1, PTS2, Pex19BS) were predicted using PTS1 Predictor (http://mendel.imp.ac.at/mendeljsp/sat/pts1/PTS1predictor.jsp) and Target signal predictor (http://216.92.14.62/Target_signal.php). Signal peptides (SPs), signal anchors (SAs), and mitochondrial targeting peptides (mTPs) were predicted using SignalP3.0 (http://www.cbs.dtu.dk/services/SignalP-3.0/), SignalP4.1 (http://www.cbs.dtu.dk/services/SignalP/), TargetP1.1 (http://www.cbs.dtu.dk/services/TargetP/), Predotar (https://urgi.versailles.inra.fr/predotar/predotar.html) and PredSL (http://aias.biol.uoa.gr/PredSL/input.html). TMDs were predicted using TMHMM2.0 (http://www.cbs.dtu.dk/services/TMHMM/) and TOPCONS (http://topcons.cbr.su.se). *Perkinsela* sp. proteins were considered to be glycosome-localized only if a clear PTS1, PTS2 or mPTS signal was present and none of the other tools produced conflicting significant predictions (Table S2.2.6.2). Search criteria were as follows. PTS1 Predictor: ‘positive’; Target signal predictor: PTS1/PTS2/mPTS cutoff < 0.01; for PTS2 signal must be localized within the first 100 N-terminal amino acids; and for mPTS, at least one TMD must be predicted by TMHMM and/or TOPCONS.

### Electron Microscopy

High-pressure freezing and freeze substitution was conducted to prepare samples for transmission electron microscopy. *P*. *pemaquidensis* cells were concentrated by centrifugation and loaded into a freezing chamber filled with 20% BSA as a cryopreservant and processed at a freezing speed > 20,000 K/s and pressure > 2,000 bar. High-pressure frozen cell pellets were directly transferred in liquid nitrogen to a freeze substitution system. Samples were fixed in 2% OsO_4_ in anhydrous acetone at −90 °C for 28 h; the temperature was then gradually raised to 20 °C through a series of incubations in acetone. Samples were subsequently transferred for embedding in a series of freshly prepared Epon solutions. Epon infiltrated samples were polymerized for 48 h at 60 °C.

Ultrathin sections were stained using the periodic acid-thiosemicarbazide-silver proteinate method^83^. This involved (i) 20–25 min incubation with 1% periodic acid in distilled water, (ii) two washes with distilled water and three 10 min washes in distilled water, (iii) 40 min incubation in 1% thiosemicarbazide in 10% acetic acid, (iv) two washes in 10% acetic acid followed by 15 min incubation in 10% acetic acid, (v) three 15 min washes in distilled water, (vi) 30 min incubation in 1% protargol (silver proteinate) in distilled water in the dark, and (vii) a final wash in distilled water. Samples were also stained with 2% uranyl acetate in 30% EtOH, post-stained with lead citrate, and examined with a JEOL JEM-2100F electron microscope.

Freeze-fracture cryo-scanning electron microscopy was carried out as follows. A suspension of *P*. *pemaquidensis* cells in seawater was transferred into a 1 mm deep hole in a sapphire disc at room temperature, and then cryo-fixed by flash freezing in liquid nitrogen. The cartridge-mounted disc was then transferred under vacuum to the cryo-attachment chamber (CryoALTO 2500; Gatan, Inc). The frozen cell pellet was fractured using a metal spike, which was heated to −95 °C *in vacuo* for 5 min in order to remove ice contamination from the surface of the freeze-fracture by sublimation. The sample was then sputter-coated for 40 s with Pt/Pd, and the disc loaded directly into the microscope using a cryo-transfer shuttle cooled with liquid nitrogen. Freeze-fractured material was observed using a JSM-7401F scanning electron microscope (JEOL Ltd) operated at 1 kV with a working distance of approximately 8 mm and a stage temperature of approximately −140 °C.

### Data Availability

The *Perkinsela* sp. and *Paramoeba pemaquidensis* nuclear genome sequences have been deposited in GenBank under the accession numbers LFNC00000000 and MUHK00000000, respectively. RNA-Seq data are deposited under the following accession numbers: GEWA00000000 (assembled transcripts based on SRX1959907) and KC534504–KC534632 (based on SL-amplified short reads in SRX255943). Mitochondrial genome sequences are as follows: KT261384-KT261386 (*Perkinsela* sp.) and KX611830 (*P*. *pemaquidensis*).

## Electronic supplementary material

Supplementary Notes + Supp. figures

Table S1.1

Table S1.3.1

Table S1.3.2

Table S1.6.1

Table S1.6.2

Table S1.6.3

Table S1.7.1

Table S2.1.1

Table S2.5.1

Table S2.5.2

Table S2.6.1

Table S2.6.2

Table S2.6.3

Table S2.8.1
