# Supplementary material for: Genome sequencing reveals metabolic and cellular interdependence in an amoeba-kinetoplastid symbiosis
Source: Sci Rep. 2017 Sep 15;7:11688. doi: 10.1038/s41598-017-11866-x (PMC5601477; doi:10.1038/s41598-017-11866-x)
Supplement: Supplementary file 1 — Supplementary Notes + Supp. figures [file 41598_2017_11866_MOESM1_ESM.pdf]

Scientific Reports—Article

Supplementary Information

Genome sequencing reveals metabolic and cellular interdependence in an amoeba-kinetoplastid symbiosis

Goro Tanifuji[1,2], Ugo Cenci[1,2], Daniel Moog[1,2], Samuel Dean[3], Takuro Nakayama[4], Vojtěch David[1,2,5], Ivan Fiala[5], Bruce A. Curtis[1,2], Shannon Sibbald[1,2], Naoko T. Onodera[1,2], Morgan Colp[1,2], Pavel Flegontov[5,6], Jessica Johnson-MacKinnon[1,2], Michael McPhee[1,2], Yuji Inagaki[4,7], Tetsuo Hashimoto[7], Steven Kelly[8], Keith Gull[3], Julius Lukeš[5,9,10], and John M. Archibald[1,2,10]

[1]Department of Biochemistry & Molecular Biology, Dalhousie University, Halifax, Nova Scotia, Canada. [2]Centre for Comparative Genomics and Evolutionary Bioinformatics, Dalhousie University, Halifax, Nova Scotia, Canada. [3]Sir William Dunn School of Pathology, University of Oxford, Oxford, United Kingdom. [4]Center for Computational Sciences, University of Tsukuba, Japan. [5]Institute of Parasitology, Biology Centre, Czech Academy of Sciences, České Budějovice, Czech Republic. [6]Life Science Research Centre, Faculty of Science, University of Ostrava, Ostrava, Czech Republic. [7]Graduate School of Life and Environmental Sciences, University of Tsukuba, Japan. [8]Department of Plant Sciences, University of Oxford, Oxford, United Kingdom. [9]Faculty of Sciences, University of South Bohemia, České Budějovice, Czech Republic. [10]Canadian Institute for Advanced Research, CIFAR Program in Integrated Microbial Biodiversity, Toronto, Canada. Correspondence and requests for materials should be addressed to J.M.A. (email: john.archibald@dal.ca).

Supplementary Note 1. Genomes, Transcriptomes and Proteomes

1.1. Strand polarity in the *Perkinsela* sp. nuclear genome

A non-random pattern of gene orientation is readily apparent in the *Perkinsela* sp. nuclear genome, similar to that seen in the genomes of trypanosomatid parasites[1,2] and to a lesser extent, that of the free-living bodonid *Bodo saltans*[3]. The average number of consecutive genes on the same strand in the *Perkinsela* sp. genome was found to be 4.5 (median = 3.0). This compares to an average of 12.3 (median = 4.0) for *Trypanosoma brucei* 427 (ver 4.2) and 39.5 (median = 15) for *Leishmania major* Friedlin (ver. 4.2). As a control, the mean and median values calculated for the *P. pemaquidensis* (i.e., host) nuclear genome were 1.7 and 1.0, respectively. Three prominent examples of strand polarity in the *Perkinsela* sp. genome are shown in Fig. S1.1. Examination of the sizes of intergenic regions between divergent and convergent gene blocks revealed that both are greater than the mean intergenic distance for the genome as a whole, which is ~515 bp (Table 1, main text). With a minimum gene block size of ≥4, the mean intergenic distances were 671 and 1,381 bp for divergent and convergent gene blocks, respectively (i.e., gene blocks whose transcription is oriented away or towards one another).

1.2. *Perkinsela* sp. mobile genetic elements

The nuclear genomes of kinetoplastids are typically rich in mobile genetic elements (or transposable elements)[1,4,5]. 62 putative proteins with similarity to retrotransposons were found in the *Perkinsela* sp. final protein set (BlastP cutoff of e<1e-10), and an additional 148 retrotransposon-like sequences were identified using tblastN against genomic scaffolds (minimum length of 100 bp and a cutoff of e<1e-10). 57 of these 148 coding regions were inspected manually, 17 of which were predicted to be pseudogenes (i.e., they were degenerate in various ways, including the presence of numerous frame shifts). The strongest matches were to the L1*Tc* element of *T. cruzi*[6], which belongs to the *ingi* family of non-LTR retrotransposons. RT-PCR (data not shown) was used to amplify a complete L1*Tc*-like sequence from *Perkinsela* sp., which had a spliced leader sequence at its 5’ end, indicating that at least some of these elements are expressed

and functional. Approximately 40 sequences showed weak similarity to the SIDER elements found in *Leishmania* genomes (Short Interspersed DEgenerated Retroposons[7]).

As a control, we searched for transposable elements in the genomic scaffolds assigned to the host of *Perkinsela* sp., i.e., *P. pemaquidensis*. Not surprisingly, >30 sequences were identified with significant matches to non-LTR retrotransposons in other amoebozoans, most notably *Entamoeba histolytica*. Importantly, none of these *P. pemaquidensis* sequences showed significant similarity to any of the retrotransposon or retrotransposon-like sequences predicted for *Perkinsela* sp., providing additional confidence in our ability to distinguish between host- and endosymbiont-derived genomic scaffolds.

1.3. *Perkinsela* sp. spliced leader RNA genes

Using the previously identified *Perkinsela* sp. spliced leader (SL) RNA gene[8] as a BlastN query, we identified >500 distinct matching sequences distributed on many different scaffolds. Interestingly, only a single intact (and presumably functional) SL RNA gene could be identified (on scaffold 688; Table S1.3.1). This gene encodes a 38 bp SL mini-exon and a 468 bp SL intron. We predict that scaffold 688 contains a tandem array of SL RNA genes, as suggested by Tanifuji et al.[8], although this could not be verified due to the presence of sequence gaps. Manual investigation of 71 of 531 SL RNA and SL RNA-like coding regions revealed genes in various stages of decay. These included genes with insertions / deletions as well as stand-alone SL introns, the latter presumably the result of reverse transcription and genomic reintegration. We also found >30 instances of ‘stand-alone’ sequences matching only the mini exon and the first 20 nucleotides of the SL RNA intron. We speculate that at least some of the retrotransposons in *Perkinsela* sp. (above), which have a reverse transcriptase (RT) domain-encoding region, provide the RT activity producing cDNA copies of the SL RNA introns, which are frequently inserted into the genome. Interestingly, we also identified 24 instances in which retrotransposon-like sequences were found in close proximity to SL RNA-like elements (Table S1.3.2). This arrangement is reminiscent of the SLACS (spliced leader-associated conserved sequence) type of site-specific non-LTR

retrotransposons found in various *Trypanosoma* species (e.g., refs[9,10]). However, the SL-RNA gene-associated retrotransposon coding regions we found in *Perkinsela* sp. do not bear any specific affinity to the SLACS-type elements of trypanosomatids; they are instead more like the *ingi*-type elements discussed in the previous section. A possible link between retrotransposon spread and SL RNA genes in the *Perkinsela* sp. nuclear genome is worthy of further investigation.

1.4. *Perkinsela* sp. leucine-rich proteins

A curious feature of the *Perkinsela* sp. nuclear genome is the presence of hundreds of hypothetical genes encoding leucine-rich proteins, most of which have little or no sequence similarity to proteins encoded by other sequenced genomes. 462 such proteins were identified by BlastP against our final set of AGUSTUS-predicted proteins, and hundreds more were detected using tBLASTn against the genomic scaffolds (e-value cutoff of <1e-10 and a minimum of 100 bp in length). Sequence similarity amongst the *Perkinsela* sp. leucine-rich proteins varies greatly: many form clusters of highly similar sequences while others are not obviously homologous to one another (i.e., they are similar solely by virtue of their repetitive, leucine-rich sequences). We could find no evidence that these leucine-rich repeat proteins are divergent homologs of the bodonin proteins of *Bodo saltans*; BLAST searches using various bodonins as queries (BS90090, BS52525, BS08390, BS73585, BS90835, BS11510, BS31875, BS37140, BS11320, BS92780) yielded no significant hits in the *Perkinsela* sp. genome. Although many of them are supported by RNA-seq expression data, the significance of the leucine-rich proteins to the biology of *Perkinsela* sp. is unclear.

1.5. Mitochondrial Genomes: Structure and Coding Capacity

The *P. pemaquidensis* mitochondrial genome is a circular mapping molecule 48,522 bp in size and with an average G/C content of 22.2% (Fig. S1.5.1). It contains 40 protein genes, single-copy large- and small-subunit rRNA genes, and 20 tRNA genes. With the exception of tRNA-Ala, all RNA and protein genes are located on the same strand. Interestingly, the cytochrome b gene is duplicated; the *cob* and *cob1* duplicates are separated by *nad3* and an unknown ORF (*orf171*). Overall, the size, coding capacity

and strand polarity of the *P. pemaquidensis* mitochondrial genome is very similar to that found in other amoebozoans such as *Acanthamoeba castellanii*, *Dictyostelium discoideum*, and *Phalansterium* sp. (see ref.[11] and references therein).

The mitochondrial genome of the endosymbiont *Perkinsela* sp. was characterized by David et al.[12]. Briefly, the genome appears to be highly reduced, with only six protein-coding genes (*cox1*, *cox2*, *cox3*, *cob*, *atp6* and *rps12*) residing on three distinct scaffolds. Ribosomal RNAs are probably fragmented and too divergent to be easily detected[12]. The true structure of the endosymbiont mitochondrial genome is unclear, but likely represents a disordered array of recombining linear fragments. Similar mitochondrial genome structures were found in *Euglena gracilis*[13], in chromerids[14] and in dinoflagellates[15]. Gene expression in the *Perkinsela* sp. mitochondrion involves extensive RNA editing in the form of U insertions and deletions.

1.6. Endosymbiotic Gene Transfer

Using the phylogenomics pipeline described in the Methods and summarized in Figure S1.6.2, we screened the *P. pemaquidensis* nuclear genome for genes of putative kinetoplastid (i.e., endosymbiont) ancestry. Our database (Table S1.6.1) was rich in genomic data from amoebozoans and kinetoplastids, so as to maximize the chances of identifying genes with anomalous evolutionary histories in our newly sequenced genomes. After a first round of ‘approximate’ maximum likelihood (ML) tree building (using FastTree) and sorting (3,846 analyzable trees in total), 1,916 trees showed *P. pemaquidensis* homologs branching with one or more amoebozoan homologs (consistent with vertical ancestry), and 10 of 35 genes / proteins initially flagged as possible EGTs were deemed worthy of further consideration. These 10 datasets were expanded after protein homolog retrieval from public databases and alignments were regenerated and trimmed. Phylogenetic trees were then reconstructed using rigorous ML and Bayesian methods. We also re-examined the scaffolds on which these genes were found and revisited the SL RNA / Total RNA library ratios for the contigs in question in order to confirm their genomic location.

Eight genes / proteins (each with RNA-seq support) were ultimately identified as being robust candidates for EGT, the topologies of five of which are consistent with

transfer from the nuclear genome of *Perkinsela* sp. to that of *P. pemaquidensis* (Table S1.6.3). Three genes with particularly strong kinetoplastid phylogenetic signatures were identified: a peptidase M20-like protein (Fig. S1.6.1a), a putative mitochondrial ADP/ATP translocase (Fig. S1.6.1b), and a generic mitochondrial carrier protein (Fig. S1.6.1c; in this case, *P. pemaquidensis* contains two homologs that branch robustly next to one another in the phylogeny, consistent with a gene duplication event after EGT). For six of these eight genes, a clear homolog still resides in the *Perkinsela* sp. nuclear genome, and in two cases (genes for a retrotransposon hotspot-like protein and a protein of unknown function; Fig. S1.6.1g and h) homologs could not be detected in any other known genome (i.e., they are *only* found in the host amoeba nuclear genome and the endosymbiont *Perkinsela* sp.).

Curiously, three of the eight EGT candidates encode proteins with mitochondrion-associated predicted functions. N-terminal sequence analyses did not provide robust support for the mitochondrial targeting of some or all of the endosymbiont-derived proteins. However, the mitochondrial ADP/ATP translocase (NPAc3631A) (Fig. S1.6.3a), as well as both of the mitochondrial carrier protein duplicates (Fig. S1.6.3b), were found to contain mitochondrial carrier protein signatures (PX[D/E]XX[K/R])[16], suggesting that these three proteins are indeed organelle localized (the homologs of these three proteins encoded in the *Perkinsela* sp. nuclear genome also contain such signatures). The biological significance of these endosymbiont-derived, host mitochondrion-targeted proteins is unclear, although it is possible that they mediate solute transport and contribute to the connectivity of the host and endosymbiont metabolisms (see main text and below).

We next carried out a phylogenetic analysis of 2,633 ‘treeable’ proteins encoded in the *Perkinsela* sp. nuclear genome. In 59% of these trees (1,559 of 2,633) the *Perkinsela* sp. homolog branched with one or more kinetoplastid homologs, as would be expected given the endosymbiont’s known ancestry. Of 2,633 proteins, two genes of potential amoebozoan origin were detected, suggestive of reverse endosymbiotic gene transfer. However, upon close inspection, these genes and the contigs they reside on could not be definitively assigned to the endosymbiont genome.

Finally, we carried out a BlastN-based analysis to explore the possibility that DNA transfers involving both coding and non-coding regions between the endosymbiont and host nuclear genomes are frequent. Here we focused only on scaffolds that were unambiguously assigned as either host or endosymbiont origin. Accounting for spurious matches between conserved genes (e.g., ribosomal RNA genes, elongation factors, heat shock proteins, etc.), we found no evidence of 'recent' and ongoing DNA transfer from endosymbiont to host or vice versa (data not shown). Overall, we conclude that DNA transfer from *Perkinsela* sp. to *P. pemaquidensis* is / has been infrequent and that the impact of EGT in shaping this obligate endosymbiotic relationship has been minimal. This may be related to potential incompatibilities between host and endosymbiont splicing and/or transcription machineries, and the presence of one or at most a few endosymbionts present per amoeba in *Paramoeba* species (e.g., refs[17,18]). In the case of mitochondria and chloroplasts, the frequency of EGT is directly related to the number of organelles per cell, with organelle lysis presumably serving as the main source of transferred DNA[19,20].

During the course of our detailed investigation of the metabolic and cell biological features of *Perkinsela* sp. and *P. pemaquidensis*, we identified several genes whose evolutionary distributions were suggestive of EGT and lateral gene transfer, despite the fact that they were not detected as putative transfers using the pipeline-based approach described in this section. For example, we identified an unusual P-type ATPase encoded by the *P. pemaquidensis* nuclear genome (protein ID c5648A), which bears similarity to kinetoplastid-type Na+/K+ transporters. Phylogenetic analyses are ambiguous as to whether or not this is an EGT.

1.7. Mitochondrial Proteome of *Perkinsela* sp.

To identify nucleus-encoded, mitochondrial-targeted proteins in *Perkinsela* sp., we combined 'top-down' and 'bottom-up' approaches. A total of 1,460 proteins were found to possess putative N-terminal mitochondrial targeting peptides by one or more of three prediction tools (TargetP, Predotar and PredSL); 424 proteins were predicted by all three (Fig. S1.7.1a). The use of Hidden Markov Model-based similarity searches using a curated set of mitochondrial proteins derived mostly from proteomic studies of *T. brucei*

brucei TREU 927[21-24] resulted in a set of 544 *Perkinsela* sp. proteins predicted to be mitochondrial. 44.9% of these 544 proteins overlapped with the set of putative mitochondrial proteins predicted by all three N-terminal targeting classifiers; an additional 37.7% of these proteins were predicted by only one or two tools. The final set of 721 proteins (Table S1.7.1) is thus composed of 94 proteins predicted solely on the basis of similarity to known mitochondrial homologs, 178 proteins which possess a predicted mitochondrial targeting peptide according to all three prediction tools, and 449 proteins predicted by both approaches.

Fig. S1.7.1b shows a KEGG functional breakdown of the 721 mitochondrial-targeted proteins. As expected, a large fraction of the proteins predicted solely on the basis of targeting peptides were hypothetical in nature; of the 721 proteins, only 213 could be functionally annotated using KEGG (with 160 unique KO tools). Consistent with the results of David et al.[12], the mitochondrial proteome of *Perkinsela* sp. is rich in proteins involved in RNA editing, transcription and translation. In addition, proteins involved in hallmark mitochondrial processes such as protein import and iron-sulfur cluster biogenesis are retained. The most apparent reduction has taken place in energy metabolism: while a reduced set of genes / proteins for respiratory chain complexes II, III, IV, and V persist, the core complex I subunits are missing entirely[12]. It is conceivable that the main biochemical role of the *Perkinsela* sp. mitochondrion is Fe-S biogenesis, with the limited suite of respiratory chain complexes serving to maintain membrane potential, which is critical for mitochondrial protein import.

Supplementary Note 2. Cell biology.

2.1. Absence of Flagellum in *Perkinsela* sp.

To determine whether *Perkinsela* sp. has the capacity to make a flagellum, we searched the genome for genes known to be associated with flagellum assembly or function. The evidence, summarized below, suggests that *Perkinsela* sp. either (i) builds a flagellum that is unlike that of any known organism (not requiring, e.g., basal bodies, the flagellum transition zone, flagellar associated tubulins, intraflagellar transport proteins (IFTs), proteins that are otherwise conserved in ciliated eukaryotes, or known kinetoplastid-

specific flagellar proteins) or (ii) does not have the capacity to build a flagellum. On balance, the evidence is most consistent with the second possibility.

The *Perkinsela* sp. nuclear genome encodes alpha, beta, and gamma tubulins but has lost tubulins associated specifically with flagellum function, such as epsilon and delta tubulin (basal body-associated), and zeta tubulin (kinetoplastid-specific, function unknown). Furthermore, the *Perkinsela* sp. alpha tubulin has a substitution at K40Q that is predicted to affect microtubule dynamics (a K370Q substitution is also found), and beta tubulin is missing two motifs required for attachment of dynein arms and specification of the central pair (below). *Bodo saltans* has retained alpha, beta, gamma, delta, epsilon, and zeta tubulins, suggesting that their apparent absence in *Perkinsela* sp. is not an artifact of evolutionary distance from *T. brucei* (Table S2.1.1).

Consistent with the tubulin substitutions described above, the *Perkinsela* sp. genome encodes no obvious homologs of a set of 21 conserved and functionally important dynein proteins. These include outer and inner arm dyneins, intermediate chain proteins, dynein docking proteins, radial spoke proteins, and central pair proteins (including the conserved protein PF16). Three proteins typically annotated as cytoplasmic dyneins are also absent, two of which have been shown to localize to the flagella of *T. brucei* and *Leishmania*, suggesting that they are functional in the flagellum in kinetoplastids. Again, homologs of these 'cytoplasmic' dyneins are found in *Bodo saltans* (Table S2.1.1).

Perkinsela sp. has lost all of the genes for proteins shown experimentally to localize to the basal body and flagellar transition zone (TZ). Many of these are ancient modules that are otherwise well conserved, including in ciliated kinetoplastids, such as components of the MKS complex (MKS6, MKS1, tectonic, etc.) and the BBSome complex (BBS5, BBS7, etc.). Canonical proteins such as SAS6 (which makes up the basal body 'cartwheel') that are otherwise highly conserved in ciliated organisms are also missing, as are the centrins (with the exception of TbCentrin4, which is a highly repetitive sequence and thus likely a false positive). Homologs of kinetoplastid-specific transition zone proteins (e.g. TZP103.8, TZP157[25]) and basal body proteins are also missing. In contrast, with the exception of BBS4, all of these proteins are found in *Bodo saltans*. The apparent absence of proteins with which to make a recognizable basal

body or transition zone—which are required for flagellum construction—is very strong evidence that *Perkinsela* sp. does not have a flagellum.

Zero of 20 known intraflagellar transport protein genes could be identified in the *Perkinsela* sp. nuclear genome, whereas *B. saltans* has retained almost all of them (18/20). To determine whether *Perkinsela* sp. has genes for any additional kinetoplastid-specific, flagellum-associated structures, we searched for genes encoding proteins of the flagellar pocket collar (1 protein[26]), bilobe (9 proteins[27]), Inv-like compartment (2 proteins[25]) and flagellar attachment zone (FAZ; 6 proteins[28]). Only one bilobe and one FAZ gene were detected, but in both cases these are long repetitive proteins and likely to be spurious matches. In contrast, *Bodo saltans* has retained 7/9 bilobe proteins, and 3/6 FAZ structures.

We next considered the results of two proteomics-based experimental investigations of the *T. brucei* flagellar proteome[29,30]. Combining these datasets with a 2-peptide cutoff resulted in a list of 433 proteins, which we then used as queries against the *Perkinsela* sp. nuclear genome. *Perkinsela* sp. has lost >85% of these proteins (only 61/433 remain). Examination of these 61 proteins suggests that they are in fact proteomics contaminants (e.g., mitochondrial proteins). In comparison, genes for 364/433 of these proteins were identified in the genome of the ciliated kinetoplastid *B. saltans*.

Hodges et al.[31] assembled a comprehensive set of proteins that are conserved in ciliated organisms but not in non-ciliated organisms. Their analyses took into account data from across the eukaryotic tree of life, including plants, excavates, holozoans and fungi. Trypanosomes have 143 genes/proteins with a ‘ciliary evolution profile’, 135 of which have homologs in *Bodo saltans*. In stark contrast, only 3 of these proteins were detected in *Perkinsela* sp. One of these proteins appears to be a mitochondrial precursor, another a lipid binding protein, and the third has three predicted transmembrane domains but no other distinguishing features.

2.2. *Perkinsela* sp. Cytoskeleton

The sub-pellicular microtubule corset-associated proteins CAP5.5, CAP5.5v and CAP51 were detected in *B. saltans*, but not in *Perkinsela* sp. This suggests that the corset is

either absent, or very different, in *Perkinsela* sp., consistent with their absence in electron micrographs. Interestingly, *Perkinsela* sp. has a clear homolog of the highly conserved protein XMAP215, which localizes to the tip of the cell body in *T. brucei* and is involved in adding microtubules subunits to the + end of microtubules. This suggests that the endosymbiont's microtubule organization bears at least some similarity to that of *T. brucei*.

2.3. Kinetoplast DNA (kDNA) Maintenance and Replication

Using *T. brucei* proteins as queries, we searched for, and found, three genes for topoisomerase II proteins in the *Perkinsela* sp. nuclear genome (Table S2.1.1). Although these proteins are likely to have additional roles beyond kDNA replication, it does suggest that the core machinery for replicating the kDNA remains. In fact, while *T. brucei* has 3 proteins in this ortholog group and *B. saltans* has 2, *Perkinsela* sp. has 5, suggesting that some expansion has taken place. In contrast, 4 known components of the tripartite attachment complex (TAC[32], TAC102[33], TAC40[34], p166[35] and p197[36]) appear to be to be missing in *Perkinsela* sp. Two components (p166 and TAC102) are also missing in *B. saltans*, but given the likely absence of the basal body and the dispersed nature of the kDNA, it seems likely that the *Perkinsela* sp. has dispensed with the TAC altogether.

2.4. *Perkinsela* sp. Kinetochores

Perkinsela sp. has clear homologs of 8 of 19 known kinetochore proteins in *T. brucei* (*B. saltans* has 18/19)[37]. No obvious pattern in the retention / loss of these genes / proteins in *Perkinsela* sp. was apparent.

2.5. A Glycosome / Peroxisome-like Organelle in *Perkinsela* sp.

A search for genes encoding peroxins, factors involved in biogenesis and protein import in peroxisome-like organelles (including glycosomes), in the nuclear genome of *Perkinsela* sp. revealed the presence of several core components. These include the specific cytosolic receptors for PTS1 (Pex5)- and PTS2 (Pex7)-mediated matrix protein import (soluble proteins), membrane proteins involved in translocation (Pex5, Pex13,

Pex14), and receptor recycling (Pex2, Pex10, Pex12, Pex1, Pex6, Pex4), as well as factors for peroxisome/glycosome division (Pex11) (Tables S2.5.1 and S2.5.2). However, proteins typically involved in the insertion of membrane proteins into peroxisome-like organelles such as Pex19, Pex3, and Pex16 could not be detected in *Perkinsela* sp. Although Pex3 and Pex19 are known to be involved in the *de novo* generation of peroxisome-like organelles in general, it is worth noting that Pex3 is apparently absent in trypanosomatids as well. In terms of primary amino acid sequence, Pex19 is not highly conserved among eukaryotes and even within kinetoplastids; it is thus possible that a divergent, but presently undetectable, Pex19 homolog is present in *Perkinsela* sp. As peroxisome-like organelles originate *de novo* from the ER, *Perkinsela* sp. might utilize alternative pathways for inserting proteins into glycosome membranes. This might take place in the ER, via vesicle fusion, or in the cytosol by alternative factors that have not been identified thus far. Genes for various other factors reported to be involved in the biogenesis of glycosomes in *Leishmania* (including ATPases, GTPases and SNAREs[38]) were found in *Perkinsela* sp. (Table S2.5.2). The metabolic processes predicted to occur in the *Perkinsela* sp. glycosome/peroxisome are summarized in Fig. S2.5.1, and discussed further in Supplementary Note 3 below).

As a point of reference, the host nuclear genome was also screened for peroxin-encoding genes, which might play a role in the peroxisomes of *P. pemaquidensis*. Similar to *Perkinsela* sp., a complete set of core components required for matrix protein import and peroxisome division was detected (Table S2.5.1). However, unlike the endosymbiont, the host seems to possess factors for peroxisomal membrane protein insertion and peroxisome biogenesis (Pex 3, Pex16 and Pex19), although a Pex4 homolog could not be detected.

2.6. Endocytosis and Exocytosis in *Perkinsela* sp. (see also Supplementary Note S3.12. Endosome / Lysosome / Reservosome)

We used a multi-tiered approach to investigate the possibility of endocytosis and exocytosis in *Perkinsela* sp. In addition to electron microscopy, this included bioinformatic analyses using OrthoFinder[39], PFAM-based domain searches, and consideration of well annotated pathway components in *T. brucei* and other

trypanosomes[40]. In some cases the *P. pemaquidensis* nuclear genome was analyzed as a control, as was the *B. saltans* genome as a control for evolutionary distance. The results are summarized in Table S2.1.1 (ESCRT components, lysosome and Golgi factors, Rabs), Table S2.6.1 (PFAM search for putative Rabs), Table S2.6.2 (SNARE and SNARE-related proteins), and Table S2.6.3 (summary of endocytosis-related proteins in host and endosymbiont).

The *Perkinsela* sp. nuclear genome has genes for a variety of components related to vesicle trafficking. Of 9 known components of the ESCRT complex in *T. brucei* (required for many trafficking processes) one is possibly present in *Perkinsela* sp. (VPS4, the same as in *B. saltans*). Similarly, clathrin and most of the SNAREs are also present, suggesting that clathrin-mediated endocytosis and vesicle fusion during trafficking occurs, respectively. *Perkinsela* sp. appears to be missing both known kinetoplastid components of the Golgi apparatus (sec34 and GRIP70), and all four components of the lysosome, including p67. All are present in *B. saltans*. Moreover, using OrthoFinder, *Perkinsela* sp. appears to be missing >85% of the Rab proteins (retaining only 2/16, compared to 15/16 in *Bodo saltans*). The two that remain are Rab1 and Rab2, which have been shown to associate with the Golgi in *T. brucei* and are required in the early secretory pathway. PFAM analysis was unsuccessful in allowing us to detect additional Rab protein genes in *Perkinsela* sp. beyond Rab1 and Rab2. For reference, four PFAM domains were found in known trypanosome RAB proteins: PFAM Ras, PFAM zf-C3HC4_3, PFAM Miro, and Pfam-B_17478. None of these yielded meaningful matches in the *Perkinsela* sp. genome. All things considered, these analyses suggest that although *Perkinsela* sp. retains the ability to perform endo- and exocytosis its intracellular trafficking machinery is highly divergent and reduced, presumably related to its intracellular lifestyle.

An important question relating to the mechanics of endocytosis is the number of membranes surrounding *Perkinsela* sp. Our results are most consistent with a single membrane, i.e., the plasma membrane of the endosymbiont is directly exposed to the cytoplasm of *P. pemaquidensis* (Fig. 1 and Fig. S2.6.1). However, it should be noted that Perkins and Castagna[41] suggested that *Perkinsela* sp. was surrounded by two membranes (plasma membrane plus outer amoeba-derived membrane). These authors

worked with a different species of amoeba than that studied here (*P. perniciosa* versus *P. pemaquidensis*). It is thus formally possible (though not likely in our opinion) that the endosymbionts of different *Paramoeba* species are surrounded by different numbers of membranes.

2.7. Membrane Transporters and Protein Secretion

As summarized in Fig. S2.7.1, we used various bioinformatic tools to identify potential membrane transporters encoded in the nuclear genomes of *Perkinsela* sp. and *P. pemaquidensis*. 226 and 66 membrane transporters were predicted in the *P. pemaquidensis* and *Perkinsela* sp. nuclear genomes, respectively, which were then annotated and classified according to transportDB[42]. Those in *Perkinsela* sp. corresponded to a variety of transporter superfamilies, with no superfamily dramatically overrepresented or depleted relative to those in other kinetoplastids. However, at the family level, the transporters appear somewhat differentially retained, with the MFS and F(V-A)ATPase families proportionally overrepresented in *Perkinsela* sp., and the AAAP, APC and ABC amino acid transporters somewhat underrepresented (Fig. S2.7.2).

We used various bioinformatic tools including TargetP, PredSL, Predotar, TMHMM 2.0 and PredGPI 2.0 to identify proteins possibly secreted by *Perkinsela* sp. into its host amoeba, as well as putative plasma membrane-localized proteins (Fig. S2.7.3). 48 proteins are predicted to be secreted from *Perkinsela* sp. to the host cytoplasm. This includes a phospholipase A2 enzyme, which may speak to the need for the degradation of phospholipid in order to obtain arachidonate, since no pathway was predicted in *Perkinsela* sp. to allow synthesis of this compound. 59 transmembrane domain-containing proteins were predicted to be plasma membrane-localized, and only two proteins were identified as possibly containing glycosylphosphatidylinositol (GPI)-anchors, consistent with the lack of obvious GPI-anchor biosynthetic capacity (data not shown).

2.8. Cell Cycle

Fig. S2.8.1 shows a KEGG-based summary of orthologous genes involved in the cell cycle of yeast. Putative orthologs of these proteins in *Perkinsela* sp. and the host

amoeba *P. pemaquidensis* were identified through KASS annotation of gene models (GHOSTX, reciprocal best hit, default eukaryotic query list + tbr). We augmented this list (Table S2.8.1) with additional cell cycle-related proteins of interest identified by reciprocal best BlastP hits using *Schizosaccharomyces pombe* and *T. brucei* queries against the *P. pemaquidensis* and *Perkinsela* sp. genomes, respectively (E-value cutoff 10^{-20}, hits beyond E-value of 10^{-100} and no worse than $\Delta_{score}20$ from the best hit were considered). We also took into consideration searches against two other amoebozoan genomes, those of *Dictyostelium discoideum* and *Entamoeba histolytica*, and additional data from GenBank and TriTrypdb (in the case of *T. brucei*).

These analyses confirm the presence of at least a handful of genes for the major phases of the cell cycle in *Perkinsela* sp. These include cyclins and CDKs, MCM proteins, cohesins, separases, and kinesins (Fig. S2.8.1). Interestingly, homologs of Origin Recognition Complex (ORC) proteins could not be detected in *Perkinsela* sp. but were found in *P. pemaquidensis*. In general the suite of cell cycle-related genes in *Perkinsela* sp. is much smaller than in *P. pemaquidensis*, although not so small that obvious host dependency must be inferred.

Supplementary Note 3. Host and Endosymbiont Metabolism

3.1. Metabolic Interdependence between *Perkinsela* sp. and *Paramoeba pemaquidensis*

Figure 3 shows a KEGG-based overview of the predicted metabolic pathways of *Perkinsela* sp. and its amoebozoan host *Paramoeba pemaquidensis*, rendered using iPath 2.0 (Interactive Pathway Explorer[43]). There is considerable overlap between the inferred metabolic capacities of the two organisms; genes / enzymes for many biochemical pathways are present in both the host and endosymbiont (shown in blue). Nevertheless, the metabolism of *Perkinsela* sp. is strikingly reduced in various areas, most notably fatty acid degradation, isoprenoid synthesis, and arginine and proline biosynthesis. Mosaic metabolic pathways discussed in the main text and below include glutathione / trypanothione metabolism (Fig. S3.1.1a), ubiquinone / terpenoid-quinone biosynthesis (Fig. 3c and Fig. S3.1.1b), arginine and proline metabolism (Fig. 3b and

Fig. S3.1.1c), fatty acid metabolism (Fig. 3a and Fig. S3.1.1d), purine metabolism (Fig. 3a and Fig. S3.1.1e), terpenoid biosynthesis (Fig. 3a and Fig. S3.1.1f), and the citrate cycle (tricarboxylic acid, or TCA cycle) (Fig. 3a and Fig. S3.1.1g). The sections that follow discuss various aspects of endosymbiont and host metabolism, concluding with a discussion of cell biological issues related to how the two might be connected.

3.2. Glycolysis and Carbohydrate Metabolism

The glycolytic pathway is the hallmark of glycosomes. In the procyclic form of *Trypanosoma brucei*, for example, the first seven enzymes of glycolysis (from hexokinase, HK, to phosphoglycerate kinase, PGK) are glycosome-localized. The glycolytic pathway is not known to be present in other peroxisome-like organelles except the glycosomes of kinetoplastids and diplonemids. The *Perkinsela* sp. nuclear genome was found to encode a complete set of glycolytic enzymes (with one isoform per enzyme), presumably enabling the conversion of glucose to pyruvate (Fig. S2.5.1). A search for the presence of potential glycosome targeting signals in the glycolytic enzymes identified in *Perkinsela* sp. showed that the first seven enzymes contain either PTS1 (PFK, GAPDH, PGK) or PTS2 (HK, ALD, TIM) signals, with the exception of PGI, whose PTS signal is unclear. No putative glycosome targeting signals were detected for the last three enzymes of the pathway (PGM, ENO, PK). These three enzymes presumably operate in the cytosol, as in other kinetoplastids, resulting in a neutral glycolysis-related ADP/ATP balance within the putative *Perkinsela* sp. glycosome.

We also found a putative PTS1 targeting signal on the *Perkinsela* sp. glycerol-3-phosphate dehydrogenase (GPDH) enzyme. GPDH could convert dihydroxyacetone phosphate (DHAP) to glycerol-3-phosphate, which is then probably shuttled to the mitochondrion where it is converted back to DHAP by a mitochondrial-targeted isoform of GPDH (which was also identified) and transported back to the glycosome (i.e., a glycerol-3-phosphate shuttle). This would ensure NAD^+/NADH balance for the glycosome-localized reactions of glycolysis. Glycerol kinase (GK), which converts glycerol-3-phosphate to ATP and glycerol under anoxic conditions in *T. brucei*, is apparently absent from the *Perkinsela* sp. genome. Net energy from glycolysis might thus be produced in the *Pekinsela* sp. cytoplasm only via the reaction catalyzed by

pyruvate kinase (Fig. S2.5.1).

The fate of the end product of glycolysis, pyruvate, is uncertain, as *Perkinsela* sp. seems to lack the enzymes known to convert pyruvate into acetyl-CoA (specifically pyruvate dehydrogenase, PDH) so as to connect glycolysis to the TCA cycle (Fig. 3a and Fig. S3.1.1g). In addition, *Perkinsela* sp. has apparently lost the first three enzymes of the TCA cycle (citrate synthase, aconitase and isocitrate dehydrogenase), despite the fact that enzymes for the remaining part of the TCA cycle from alpha-ketoglutarate to oxaloacetate seem to be present and mitochondrion-localized (Fig. 3a and Table S2.5.2). Nevertheless, an apparently cytosolic protein that *Perkinsela* sp. might use to utilize pyruvate is alanine aminotransferase (AAT). This enzyme catalyzes the conversion of pyruvate and glutamate to alpha-ketoglutarate and alanine and could thus serve to link glycolysis, amino acid metabolism and the mitochondrial TCA cycle in *Perkinsela* sp.

In addition to the mitochondrion-targeted enzymes of the incomplete TCA cycle, *Perkinsela* sp. possesses at least two potential TCA cycle enzymes—malate dehydrogenase, MDH, and fumarate reductase, FR—that might be glycosome-targeted (they possess predicted PTS1 signals) (Table S2.5.2). Together with phosphoenolpyruvate carboxykinase (PEPCK, which has a predicted PTS1 signal) and a malic enzyme (ME, which is PTS1-positive), MDH and FR probably connect the glycolytic pathway to glycosomal succinate synthesis and the incomplete mitochondrial TCA cycle (Fig. 3a and Fig. S3.1.1g). If phosphoenol pyruvate from the cytosolic portion of glycolysis is used as an initial metabolite for this pathway, net ATP could be produced within the putative *Perkinsela* sp. glycosome, with the total ADP/ATP being the same that is produced from a complete run-through of the glycolysis pathway (utilizing cytosolic PK). *Perkinsela* sp. appears to lack the ability to perform gluconeogenesis: although it possesses a PEPCK, it apparently lacks genes for fructose-1,6-bisphosphatase (FBP) and pyruvate carboxylase (PC) enzymes. Pyruvate, if produced at all by glycolysis, might be converted to malate by glycosomal-localized ME or possibly released into the cytoplasm of the host amoeba cell by a mechanism similar to that occurring in the blood stages of *T. brucei* (Fig. S2.5.1).

With respect to carbohydrate utilizing enzymes, while the composition of the

hypothetical *Perkinsela* sp. glycosome is very similar to the *T. brucei* glycosome, there are also significant differences between the two. In contrast to *T. brucei*, which contains a complete or near-complete pentose phosphate pathway (PPP), we found no evidence for glycosome-targeted PPP enzymes in *Perkinsela* sp. In fact, the endosymbiont might even have lost the oxidative part of the PPP, whereas the reductive portion of the pathway seems to be located in its cytoplasm.

The presence and subcellular distributions of glycolytic enzymes for *P. pemaquidensis* was also investigated. As in *Pekinsela* sp., a complete set of glycolysis enzymes could be identified in the host nuclear genome. With the exception of TPI/TIM for which a putative PTS1 signal was predicted, none of the core glycolytic enzymes showed any clear evidence for targeting beyond the cytoplasm of the host cell. Glycolysis thus most likely occurs in the cytoplasmic compartment of *P. pemaquidensis*. In addition to the existence of a complete glycolytic pathway, the host cell harbors the enzymatic potential to perform gluconeogenesis (genes for FBP, PEPCK and PC enzymes are present), in contrast to the endosymbiont.

3.3. Fatty Acid Beta-oxidation

Several genes for enzymes involved in the beta-oxidation of fatty acids were detected in the *Perkinsela* sp. nuclear genome. In contrast, the host cell contains a complete set of beta-oxidation enzymes, some of which possess potential peroxisomal targeting signals (Table S2.5.2).

3.4. Nucleotide and Nucleotide-sugar Metabolism

In contrast to *T. brucei*, the putative glycosome-like organelle of *Perkinsela* sp. does not appear to contain any enzymes for sugar-nucleotide metabolism, involving nucleotides bound to glucose, galactose, fucose or mannose. In fact, we could not identify homologs for most of the *T. brucei* proteins involved in these processes at all. Nevertheless, adenylate kinase (AK) and AMP deaminase (AMPD) enzymes, both with PTS1 signals at their C-termini (Table S2.5.2), are encoded in the *Perkinsela* sp. genome and, therefore, most likely localized to the glycosome. In addition to these two enzymes, which are presumably capable of utilizing ADP/ATP, we found a gene

encoding a homolog of PMP47, an ADP/ATP transporter, which might reside in the membrane of the putative *Perkinsela* sp. glycosome and play a key role in mediating metabolite exchange.

Consideration of genomic data suggests that *Perkinsela* sp. might play an important role in phosphate metabolism of nucleotides for RNA and DNA. While nucleoside monophosphate kinases for guanine, cytosine and uridine were found in the nuclear genome of *Perkinsela* sp., we could not find their counterparts in the amoeba host nuclear genome. Enzymes facilitating conversion between triphosphate and diphosphate nucleoside forms are encoded by both nuclear genomes. *Perkinsela* sp. also seems to possess a homolog of ecto-nucleotidase (Ecto-NTPDase), an enzyme associated with increased virulence in trypanosomes[44]. Ecto-NTPDase dephosphorylates free nucleotide phosphates in the extracellular space of *T. cruzi*, which are then imported separately.

3.5. ROS Metabolism (Trypanothione Metabolism)

As noted elsewhere, enzymes for kinetoplastid-specific trypanothione metabolism are encoded in the nuclear genome of *Perkinsela* sp. However, none of these proteins were found to contain putative targeting signals for glycosomal import. Trypanothione metabolism in *Perkinsela* sp. might therefore be mainly cytosolic and in part mitochondrial. As there is apparently no beta-oxidation of fatty acids, which is usually a source of reactive oxygen species (ROS) in peroxisome-like organelles, there would seem to be no requirement for a glycosome-localized ROS detoxification system in *Perkinsela* sp.

3.6. Amino Acid Metabolism

We identified several nucleus-encoded, glycosome-targeted enzymes putatively involved in amino acid metabolism in *Perkinsela* sp., including S-adenosylmethionine synthetase (MAT) and homoserine kinase (HSK), both of which produced robust PTS1 signal predictions (Table S2.5.2). Two additional enzymes, aspartate aminotransferase (GOT) and S-adonosylmethionine homocysteinase (SAH; see acidocalcisome section below), are also potentially glycosomal, although their PTS1 predictions are less

convincing, and they could thus be cytosolic. In other eukaryotes, GOT is typically localized to the mitochondrion, but in *Perkinsela* sp. there is no indication of mitochondrial targeting signals (Table S2.5.2). However, as GOT catalyzes the conversion of aspartate and alpha-ketoglutarate to oxaloacetate and glutamate, it could play an important role in *Perkinsela* sp. cells, serving to connect (glycosomal or cytosolic) amino acid metabolism to the incomplete mitochondrial TCA cycle of the kinetoplastid endosymbiont (Fig. 3 and Fig. S3.1.1g).

3.7. Sterol / Isoprenoid Metabolism

No genes encoding enzymes for sterol metabolism were identified in the *Perkinsela* sp. genome. However, a mevalonate kinase (MVK) enzyme with a potential PTS1 targeting signal was found. The remaining mevalonate pathway (HMGCoA, PMVK and MVD) is most likely cytosolic.

3.8. Glycosome-associated Protein Folding, Regulation and Signaling

A Hsp40 molecular chaperone (with a PTS1 signal) and a ubiquitin-specific protease-like DUB protein were the only "cell biological" factors of the putative *Perkinsela* sp. glycosome that could be reliably identified via BLAST searches using the *T. brucei* glycosomal proteome as queries. Additional potential glycosome-targeted candidates in this category are three kinases (PKA, STK, and CK1) but their PTS1 signal predictions are weak and they could well be cytosolic. Evidence for glycosomal phosphatases in *Perkinsela* sp. is lacking. In *T. brucei*, kinases and phosphatases could be involved in the regulation of glycosomal or glycosome-associated proteins; whether this is true for *Perkinsela* sp. is unclear.

3.9. Glycosomal Transporters and Membrane Proteins

Beyond the ADP/ATP transporter PMP47 (see above), little could be inferred about glycosomal membrane proteins in *Perkinsela* sp. As noted above, homologs of the classical peroxisome-type, membrane-associated import factors could not be identified, i.e., Pex3, Pex16, and Pex19. Nevertheless, potential mPTS signals, which are Pex19 binding sites, were found on Pex10, Pex11, and Pe14 (Table S2.5.2). If a Pex19

homolog is truly absent, proteins destined for the *Perkinsela* sp. glycosome must use alternative pathways (see below).

3.10. PTS1 Motifs in *Perkinsela* sp. Proteins

We performed a systematic search for proteins containing potential C-terminal PTS1 signals based on information taken from Jamhade et al.[38], i.e., the presence of the tri-peptide [ASCGPNYTV][KNRHQDS][LMVAIF] within the last four amino acid positions (accounting for the possible presence of a stop codon (*) in the fourth position). 321 candidate proteins were identified from an interim set of 5,302 predicted *Perkinsela* sp. proteins. Of these 321 PTS1 motif-containing proteins, several belong to the glycolysis pathway discussed above, as well as GOT1, MAT, MVK, Hsp40, AMPD, ME, PPase (see below), PEPCK, MDH, AK, HSK and DUB. However, the majority of these were not predicted to contain PTS1 signals with the other prediction tools used in this study (i.e., PTS1 predictor and Target signal predictor). This set thus presumably contains many false positives, but nevertheless served to expand the list of putative glycosomal proteins in *Perkinsela* sp. for future study (Table S2.5.2). Proteins with positive PTS1 predictions using the motif search above as well as with PTS1 predictor and Target signal predictor, and for which no conflicting targeting signals were detected, include: an ankyrin and TPR-domain containing protein; a AAA ATPase domain containing protein ('fidgetin-like'); VPS16, a protein with homology to vacuolar sorting protein 16; GAP, a potential GTPase activating protein; NRDE, a member of a protein family predicted to play a role in protein secretion and Golgi organization (a potential phosphatase); and several proteins with unknown functions (DUF866, UKF1-5). How many of these are actually glycosome-localized is unclear. All things considered, the putative glycosome of *Perkinsela* sp. possesses many, but by no means all, of the biochemical features associated with glycosomes in other kinetoplastids.

3.11. Autophagy / Pexophagy

Autophagy is the process by which cells degrade macromolecules and organelles; pexophagy refers to the breakdown of peroxisome-like organelles, including glycosomes. In *Trypanosoma,* pexophagy plays a role in regulation of the number of

glycosomes per cell during the different life cycle stages and the transitions between them. There are three general types of autophagy, (i) cytoplasm-to-vacuole-targeting (Cvt), (ii) microautophagy and (iii) macroautophagy, each with their own diagnostic molecular markers. In trypanosomatids, Cvt seems to be entirely absent whereas basic protein factors for micro- / macroautophagy have been identified[45]. A BLAST search using all potential *T. brucei* autophagy / pexophagy components as queries suggests that this cellular process is completely absent in *Perkinsela* sp. Although a few proteins with very weak sequence similarity to certain kinases involved in autophagy were retrieved, clear homologs of known autophagy-related gene (ATG) factors were not found (Fig. S3.11.1). It thus seems unlikely that *Perkinsela* sp. operates a classical micro-/macroautophagy pathway. Certainly *Perkinsela* sp. would not seem to use autophagy to degrade its single large mitochondrion, although how the organism's glycosomes are recycled, if at all, is unclear. Alternative mechanisms may exist (see below).

3.12. Endosome / Lysosome / Reservosome

In addition to glycosomes, other single membrane-bound organelles include endosomes and lysosomes / lysosome-related organelles (LROs). In trypanosomatids, the endocytic pathway ends with what is called the 'reservosome', a lysosome-related organelle that, in addition to protein degradation, serves as a storage compartment for both lipids and proteins. Reservosomes thus contain a characteristic set of enzymes involved in protein and lipid metabolism, as well as several typical endosome- / lysosome-specific factors that have been characterized in proteomics studies of *Trypanosoma*.

The endosomal pathway usually starts with the generation of early endosomes from the trans-Golgi network (TGN) that can fuse with endocytic vesicles. Whereas *Perkinsela* sp. cells appear capable of performing endocytosis (above), we were not able to determine whether this reduced kinetoplastid endosymbiont is likely to be capable of classical endosome formation. While a large set of endosome-associated proteins were identified in the nuclear genome of the host amoeba *P. pemaquidensis*, very few clear homologs were identified in *Perkinsela* sp. (Fig. S3.12.1). As discussed above, it is not clear whether *Perkinsela* sp. possesses a classical Golgi apparatus, and

the set of genes encoding Rab proteins, small GTPases diagnostic of the different endosome / lysosome maturation steps, is greatly reduced and seems restricted to the presence of the ER/Golgi-specific Rab1 and Rab2 proteins (Table S2.5.2). In addition, genes encoding most proteins associated with the endosomal sorting complex required for transport (ESCRT) could not be found in the *Perkinsela* sp. genome (Fig. S3.12.1 and Table S2.1.1). Altogether, based on protein presence / absence alone, there is very little evidence for the existence of a classical endosomal / lysosomal system in *Perkinsela* sp. This raises the question of where the observed endocytic vesicles go.

Neither a screen for endosomal / lysosomal components via KEGG annotation nor a BLAST-based search using 22 reservosome-specific proteins from *T. cruzi*[46] as queries resulted in clear evidence for the presence of endosomes or lysosomes in *Perkinsela* sp. Of the 17 reservosome factors known to be specifically involved in protein metabolism, the *Perkinsela* sp. nuclear genome was found to encode only two convincing homologs, a S-adenosylhomocysteine hydrolase (SAH) and a M20/M25/M40 peptidase. However, whereas the peptidase does not contain any predicted targeting signal, SAH contains a putative PTS1 motif (Table S2.5.2). Additional searches for endosomal/lysosomal proteins revealed that *Perkinsela* sp. contains a homolog of endosomal integral membrane protein 70 (Emp70). While the presence of Emp70 is consistent with the existence of an endosome / lysosome / reservosome in *Perkinsela* sp., it cannot be ruled out that this protein has adopted some other function(s) in vesicular regulation unrelated to these pathways. The same may be true for the vacuolar protein sorting protein 45 (Vps45), a homolog for which was detected in the *Perkinsela* sp. genome, which could somehow be linked to ESCRT, as well as other vesicular processes (Fig. S3.12.1). A more detailed search for *Perkinsela* sp. proteins with homology to known *Trypanosoma* reservosome-specific pumps, channel proteins, and metabolic factors (e.g., those involved in lipid metabolism) will perhaps shed further light on these uncertainties.

3.13. Acidocalcisome

Acidocalcisomes are single membrane-bound organelles dedicated to the storage of inorganic phosphate and calcium. They are present in diverse cells ranging from

bacteria to humans and have characteristics in common with LROs (lysosome-related organelles), including shared protein targeting mechanisms, protein content, morphology, and acidity. We carried out a BLAST analysis of the *Perkinsela* sp. nuclear genome using 14 experimentally verified acidocalcisomal proteins from *T. brucei*[47] as queries. We identified clear homologs of numerous core acidocalcisomal components, including a vacuolar H^+-PPase, a vacuolar- Ca^{2+}-ATPase, a vacuolar H^+ATPase (a+d subunits), a vacuolar iron transporter, a potential phosphate transporter and a putative vacuolar soluble phosphatase. Remarkably, however, the latter protein was found to contain a weakly predicted PTS1 signal, consistent with a glycosomal localization (see PPase in Fig. S2.5.1). Homologs of the *T. brucei* acidocalcisomal IP_3 receptor, the vacuolar transporter chaperone 1/4, the zinc transporter and the acid phosphatase could not be detected in the *Perkinsela* sp. genome. Furthermore, *Perkinsela* sp. appears to lack clear homologs of the AP-3 complex subunits, which in *T. brucei* are involved in acidocalcisome biogenesis. However, *Perkinsela* sp. seems to contain homologs of AP-1, AP-2 and AP-4 complexes, which are known to be involved in sorting to the endosomal compartment, the formation of endocytic vesicles at the plasma membrane, and vesicular traffic to the endosomal/lysosomal system, respectively (Fig. S3.13.1). While it is unclear whether acidocalcisome generation is facilitated by some of these AP-complex subunits, our analyses support the existence of a basic acidocalcisome in *Perkinsela* sp. that could be involved in phosphate storage and osmoregulation. Recent work on the role of the *T. brucei* acidocalcisome in autophagy[48] suggests that the apparent lack of classical autophagy and endosomal/lysosomal systems in *Perkinsela* sp. might be compensated for by the presence of an acidocalcisome (see below).

3.14 Endocytosis and Endosymbiont-Host Metabolic Integration

Our discovery of a glycosome in the highly reduced cytoplasm of *Perkinsela* sp. (Fig. S2.5.1), as exists in other kinetoplastids and the deep branching diplonemids, underscores the importance of the organelle to the biochemistry and metabolism of these unusual protists. The strongest evidence for the existence of a glycosome / peroxisome-like organelle in *Perkinsela* sp. comes from the identification of a near-

complete set of peroxin genes in the nuclear genome, as well as the fact that a significant part of the glycolytic pathway appears to be organelle-targeted. While the mechanism(s) for glycosomal membrane protein targeting could not be inferred (Pex3, Pex16, Pex19 protein genes were not found), it is worth noting that a Pex3 homolog is also missing in *T. brucei*, where the receptor, Pex19, is nevertheless present and Pex16 was recently identified[49]. In *Trypanosoma*, the Pex16 protein also seems to play a role in glycosome biogenesis, making its apparent absence in *Perkinsela* sp. somewhat perplexing. However, numerous other proteins involved in glycosome biogenesis are encoded in the *Perkinsela* sp. genome, and collectively the evidence for a glycosome / peroxisome-like organelle in *Perkinsela* sp. is robust. Here we discuss how the *Perkinsela* sp. glycosome might interact with other single-membrane bound organelles in the cell and, more generally, serve as a link between the metabolisms of the endosymbiont and its amoebozoan host.

As *Perkinsela* sp. apparently uses endocytosis to internalize material from the cytoplasm of *P. pemaquidensis* (Fig. 1 and Fig. S2.6.1), it is worth considering the fate and significance of the ingested materials / metabolites. The textbook endocytic pathway involves the fusion of plasma membrane-derived vesicles with early endosomes generated from the trans-Golgi network, which then mature to late endosomes and lysosomes, where digestion ultimately takes place. As mentioned above, it is unclear whether *Perkinsela* sp. cells are actually capable of endosome formation. Several observations, including the retention of a highly reduced set of Rab proteins (only Rab1 and Rab2), the apparent lack of an ESCRT system and, if present at all, a highly reduced / peculiar Golgi apparatus, make the presence of a classical endosomal / lysosomal pathway in *Perkinsela* sp. unlikely.

If there are no endosomes in *Perkinsela* sp., where might endocytic vesicles go? One intriguing possibility is that they fuse with the putative glycosome / peroxisome-like organelle. The glycosome is predicted to be rich in metabolic capacity, especially relative to the modest overall metabolic potential of this reduced obligate endosymbiont. The *Perkinsela* sp. glycosome is predicted to harbor various glycolytic enzymes, as well as enzymes involved in the metabolism of nucleotides, mevalonate and amino acids (Fig. S2.5.1). Fusion of endocytic vesicles with glycosomes would allow metabolites

derived from the amoeba cytoplasm to feed directly into the biochemical pathways of *Perkinsela* sp. Consistent with this hypothesis is the close association of, and perhaps fusion of, vesicles of differing electron densities within the *Perkinsela* sp. cytoplasm (Fig. 1, Fig. S2.6.1). Plasma membrane-derived vesicles could also fuse directly with acidocalcisomes (lysosome-related organelles), which could perform at least some of the degradative functions in the *Perkinsela* sp. cell. As noted above, however, *Perkinsela* sp. appears to have lost most of the classical lysosomal lytic enzymes presumed to have been present in its free-living ancestors, including several peptidases and glucosidases. The precise role(s) of an acidocalcisome-like organelle in *Perkinsela* sp. is at present unclear.

Another possibility is that glycosomes and acidocalcisomes represent different stages in the endocytic / digestive pathway in *Perkinsela* sp. Under this scenario, endocytic vesicles would first fuse with glycosomes, where ingested material is first metabolized. These compartments could then mature into acidocalcisomes, or, alternatively, fuse with preexisting acidocalcisomes. In this case, very little in the way of peroxisomal membrane (metabolite) transporters would be necessary in the glycosomal membranes, as they could be recycled from the acidocalcisomal membranes at the time of fusion; this could explain the apparent lack of Pex16 and Pex19 homologs in *Perkinsela* sp. At the same time, pexophagy would be unnecessary, as glycosomes would ‘disappear’ as part of the maturation / fusion process. In support of this fusion / maturation hypothesis is the presence of SAH and PPase in *Perkinsela* sp., enzymes which in *Trypanosoma* are localized to the reservosome and acidocalcisome, respectively, and possess putative PTS1 targeting signals (SAH and PPase are some of the few reservosome / acidocalcisome enzymes that could be detected in *Perkinsela* sp.; Table S2.5.2). The PTS1 targeting predictions are admittedly weak; it is unclear whether these proteins are indeed targeted to the *Perkinsela* sp. glycosome or some derivation of it. Regardless, the *Perkinsela* sp. glycosome appears to have evolved in concert with the organism’s intimate and obligate association with its amoeba host.

References for Supplementary Notes

1 El-Sayed, N. M. *et al.* Comparative genomics of trypanosomatid parasitic protozoa. *Science* **309**, 404-409 (2005).

2 Stuart, K. D. & Myler, P. J. in *Genomics and evolution of microbial eukaryotes* (eds L. A. Katz & D. Bhattacharya) Ch. 10, 155-168 (Oxford University Press., 2006).

3 Jackson, A. P. *et al.* Kinetoplastid phylogenomics reveals the evolutionary innovations associated with the origins of parasitism. *Current Biology* **26**, 161-172, doi:10.1016/j.cub.2015.11.055 (2016).

4 Bringaud, F., Ghedin, E., El-Sayed, N. M. & Papadopoulou, B. Role of transposable elements in trypanosomatids. *Microbes and Infectivity* **10**, 575-581, doi:10.1016/j.micinf.2008.02.009 (2008).

5 Jackson, A. P., Quail, M. A. & Berriman, M. Insights into the genome sequence of a free-living Kinetoplastid: *Bodo saltans* (Kinetoplastida: Euglenozoa). *BMC Genomics* **9**, 594, doi:10.1186/1471-2164-9-594 (2008).

6 El-Sayed, N. M. *et al.* The genome sequence of *Trypanosoma cruzi*, etiologic agent of Chagas disease. *Science* **309**, 409-415 (2005).

7 Smith, M., Bringaud, F. & Papadopoulou, B. Organization and evolution of two SIDER retroposon subfamilies and their impact on the *Leishmania* genome. *BMC Genomics* **10**, 240, doi:10.1186/1471-2164-10-240 (2009).

8 Tanifuji, G. *et al.* Genomic characterization of *Neoparamoeba pemaquidensis* (Amoebozoa) and its kinetoplastid endosymbiont. *Eukaryotic Cell* **10**, 1143-1146, doi:10.1128/EC.05027-11 (2011).

9 Aksoy, S., Williams, S., Chang, S. & Richards, F. F. SLACS retrotransposon from *Trypanosoma brucei* gambiense is similar to mammalian LINEs. *Nucleic Acids Research* **18**, 785-792 (1990).

10 Patrick, K. L. *et al.* Genomic rearrangements and transcriptional analysis of the spliced leader-associated retrotransposon in RNA interference-deficient *Trypanosoma brucei*. *Molecular Microbiology* **67**, 435-447, doi:10.1111/j.1365-2958.2007.06057.x (2008).

11 Pombert, J. F. *et al.* The complete mitochondrial genome from an unidentified *Phalansterium* species. *Protist Genomics* **2**, 25-32. (2014).

12 David, V. *et al.* Gene loss and error-prone RNA editing in the mitochondrion of *Perkinsela*, an endosymbiotic kinetoplastid. *mBio* **6**, e01498-01415 (2015).

13 Dobakova, E., Flegontov, P., Skalicky, T. & Lukeš, J. Unexpectedly streamlined mitochondrial genome of the euglenozoan *Euglena gracili*s. *Genome biology and evolution* **7**, 3358-3367, doi:10.1093/gbe/evv229 (2015).

14 Flegontov, P. *et al.* Divergent mitochondrial respiratory chains in phototrophic relatives of apicomplexan parasites. *Molecular Biology & Evolution* **32**, 1115-1131, doi:10.1093/molbev/msv021 (2015).

15 Nash, E. A., Nisbet, R. E., Barbrook, A. C. & Howe, C. J. Dinoflagellates: a mitochondrial genome all at sea. *Trends in Genetics* **24**, 328-335 (2008).

16 Zara, V. *et al.* Biogenesis of yeast dicarboxylate carrier: the carrier signature facilitates translocation across the mitochondrial outer membrane. *Journal of Cell Science* **120**, 4099-4106, doi:10.1242/jcs.018929 (2007).
17 Dyková, I., Fiala, I., Lom, J. & Lukeš, J. *Perkinsiella amoebae*-like endosymbionts of *Neoparamoeba* spp., relatives of the kinetoplastid *Ichthyobodo*. *European Journal of Protistology* **39**, 37-52 (2003).
18 Dyková, I. *et al. Neoparamoeba branchiphila* n. sp., and related species of the genus *Neoparamoeba* Page, 1987: morphological and molecular characterization of selected strains. *Journal of Fish Diseases* **28**, 49-64, doi:10.1111/j.1365-2761.2004.00600.x (2005).
19 Barbrook, A. C., Howe, C. J. & Purton, S. Why are plastid genomes retained in non-photosynthetic organisms? *Trends in Plant Science* **11**, 101-108 (2006).
20 Smith, D. R., Crosby, K. & Lee, R. W. Correlation between nuclear plastid DNA abundance and plastid number supports the limited transfer window hypothesis. *Genome Biology and Evolution* **3**, 365-371, doi:10.1093/gbe/evr001 (2011).
21 Butter, F. *et al.* Comparative proteomics of two life cycle stages of stable isotope-labeled *Trypanosoma brucei* reveals novel components of the parasite's host adaptation machinery. *Molecular and Cellular Proteomics* **12**, 172-179, doi:10.1074/mcp.M112.019224 (2013).
22 Gunasekera, K., Wuthrich, D., Braga-Lagache, S., Heller, M. & Ochsenreiter, T. Proteome remodelling during development from blood to insect-form *Trypanosoma brucei* quantified by SILAC and mass spectrometry. *BMC Genomics* **13**, 556, doi:10.1186/1471-2164-13-556 (2012).
23 Niemann, M. *et al.* Mitochondrial outer membrane proteome of *Trypanosoma brucei* reveals novel factors required to maintain mitochondrial morphology. *Molecular and Cellular Proteomics* **12**, 515-528, doi:10.1074/mcp.M112.023093 (2013).
24 Urbaniak, M. D., Guther, M. L. & Ferguson, M. A. Comparative SILAC proteomic analysis of *Trypanosoma brucei* bloodstream and procyclic lifecycle stages. *PLoS one* **7**, e36619, doi:10.1371/journal.pone.0036619 (2012).
25 Dean, S., Moreira-Leite, F., Varga, V. & Gull, K. Cilium transition zone proteome reveals compartmentalization and differential dynamics of ciliopathy complexes. *Proceedings of the National Academy of Sciences USA* **113**, E5135-5143, doi:10.1073/pnas.1604258113 (2016).
26 Bonhivers, M., Nowacki, S., Landrein, N. & Robinson, D. R. Biogenesis of the trypanosome endo-exocytotic organelle is cytoskeleton mediated. *PLoS Biology* **6**, e105, doi:10.1371/journal.pbio.0060105 (2008).
27 Morriswood, B. *et al.* Novel bilobe components in *Trypanosoma brucei* identified using proximity-dependent biotinylation. *Eukaryotic Cell* **12**, 356-367, doi:10.1128/EC.00326-12 (2013).
28 Sunter, J. D., Varga, V., Dean, S. & Gull, K. A dynamic coordination of flagellum and cytoplasmic cytoskeleton assembly specifies cell morphogenesis in trypanosomes. *Journal of Cell Science* **128**, 1580-1594, doi:10.1242/jcs.166447 (2015).

29 Broadhead, R. *et al.* Flagellar motility is required for the viability of the bloodstream trypanosome. *Nature* **440**, 224-227, doi:10.1038/nature04541 (2006).

30 Hart, S. R. *et al.* Analysis of the trypanosome flagellar proteome using a combined electron transfer/collisionally activated dissociation strategy. *Journal of the American Society for Mass Spectrometry* **20**, 167-175, doi:10.1016/j.jasms.2008.08.014 (2009).

31 Hodges, M. E., Scheumann, N., Wickstead, B., Langdale, J. A. & Gull, K. Reconstructing the evolutionary history of the centriole from protein components. *Journal of Cell Science* **123**, 1407-1413, doi:10.1242/jcs.064873 (2010).

32 Povelones, M. L. Beyond replication: division and segregation of mitochondrial DNA in kinetoplastids. *Molecular and Biochemical Parasitology* **196**, 53-60, doi:10.1016/j.molbiopara.2014.03.008 (2014).

33 Trikin, R. *et al.* TAC102 Is a novel component of the mitochondrial genome segregation machinery in trypanosomes. *PLoS Pathogy* **12**, e1005586, doi:10.1371/journal.ppat.1005586 (2016).

34 Schnarwiler, F. *et al.* Trypanosomal TAC40 constitutes a novel subclass of mitochondrial beta-barrel proteins specialized in mitochondrial genome inheritance. *Proceedings of the National Academy of Sciences USA* **111**, 7624-7629, doi:10.1073/pnas.1404854111 (2014).

35 Zhao, Z., Lindsay, M. E., Roy Chowdhury, A., Robinson, D. R. & Englund, P. T. p166, a link between the trypanosome mitochondrial DNA and flagellum, mediates genome segregation. *EMBO Journal* **27**, 143-154, doi:10.1038/sj.emboj.7601956 (2008).

36 Gheiratmand, L., Brasseur, A., Zhou, Q. & He, C. Y. Biochemical characterization of the bi-lobe reveals a continuous structural network linking the bi-lobe to other single-copied organelles in *Trypanosoma brucei*. *The Journal of Biological Chemistry* **288**, 3489-3499, doi:10.1074/jbc.M112.417428 (2013).

37 Akiyoshi, B. & Gull, K. Discovery of unconventional kinetochores in kinetoplastids. *Cell* **156**, 1247-1258, doi:10.1016/j.cell.2014.01.049 (2014).

38 Jamdhade, M. D. *et al.* Comprehensive proteomics analysis of glycosomes from *Leishmania donovani*. *OMICS* **19**, 157-170, doi:10.1089/omi.2014.0163 (2015).

39 Emms, D. M. & Kelly, S. OrthoFinder: solving fundamental biases in whole genome comparisons dramatically improves orthogroup inference accuracy. *Genome Biology* **16**, 157, doi:10.1186/s13059-015-0721-2 (2015).

40 Field, M. C. & Carrington, M. The trypanosome flagellar pocket. *Nature Reviews Microbiology* **7**, 775-786, doi:10.1038/nrmicro2221 (2009).

41 Perkins, F. O. & Castagna, M. Ultrastructure of the Nebenkörper or 'secondary nucleus' of the parasitic amoeba *Paramoeba perniciosa* (Amoebida, Paramoebidae). *Journal of Invertebrate Pathology* **17**, 186-193 (1971).

42 Ren, Q., Chen, K. & Paulsen, I. T. TransportDB: a comprehensive database resource for cytoplasmic membrane transport systems and outer membrane channels. *Nucleic Acids Research* **35**, D274-279, doi:10.1093/nar/gkl925 (2007).

43 Yamada, T., Letunic, I., Okuda, S., Kanehisa, M. & Bork, P. iPath2.0: interactive pathway explorer. *Nucleic Acids Research* **39**, W412-415, doi:10.1093/nar/gkr313 (2011).

44 Santos, R. F. *et al.* Influence of ecto-nucleoside triphosphate diphosphohydrolase activity on *Trypanosoma cruzi* infectivity and virulence. *PLoS Neglected Tropical Diseases* **3**, e387, doi:10.1371/journal.pntd.0000387 (2009).
45 Herman, M., Gillies, S., Michels, P. A. & Rigden, D. J. Autophagy and related processes in trypanosomatids: insights from genomic and bioinformatic analyses. *Autophagy* **2**, 107-118 (2006).
46 Sant'Anna, C. *et al.* Subcellular proteomics of *Trypanosoma cruzi* reservosomes. *Proteomics* **9**, 1782-1794, doi:10.1002/pmic.200800730 (2009).
47 Huang, G. *et al.* Proteomic analysis of the acidocalcisome, an organelle conserved from bacteria to human cells. *PLoS Pathogens* **10**, e1004555, doi:10.1371/journal.ppat.1004555 (2014).
48 Li, F. J. & He, C. Y. Acidocalcisome is required for autophagy in *Trypanosoma brucei*. *Autophagy* **10**, 1978-1988, doi:10.4161/auto.36183 (2014).
49 Kalel, V. C., Schliebs, W. & Erdmann, R. Identification and functional characterization of *Trypanosoma brucei* peroxin 16. *Biochimica et Biophysica Acta* **1853**, 2326-2337, doi:10.1016/j.bbamcr.2015.05.024 (2015).
50 Guther, M. L., Urbaniak, M. D., Tavendale, A., Prescott, A. & Ferguson, M. A. High-confidence glycosome proteome for procyclic form *Trypanosoma brucei* by epitope-tag organelle enrichment and SILAC proteomics. *Journal of Proteome Research* **13**, 2796-2806, doi:10.1021/pr401209w (2014).
51 Kanehisa, M., Sato, Y., Kawashima, M., Furumichi, M. & Tanabe, M. KEGG as a reference resource for gene and protein annotation. *Nucleic Acids Research* **44**, D457- 462, doi:10.1093/nar/gkv1070 (2016).

Supplementary Figure Legends

Fig. S1.1. Strand polarity in the *Perkinsela* sp. nuclear genome. **(a)** A 48 Kb region of scaffold 4 (positions 187,000-234,899); **(b)** a 130 Kb region of scaffold 1 (147,000-277,00); **(c)** a 131 Kb region of scaffold 9 (3,000-134,167). Each rectangle represents a single gene. Genes on the forward strand are on the top of each panel (oriented left to right), genes on the bottom are on the bottom (right to left). Blocks of genes on each scaffold are highlighted. **(d)** Intergenic spacer sizes between gene blocks calculated using four different minimum block sizes. Divergent gene blocks are those whose transcription is oriented away from each other (i.e., on '-' and '+' strands), convergent gene blocks ('+ -') point towards each another.

Fig. S1.5.1. Mitochondrial genome of *Paramoeba pemaquidensis* CCAP 1560/4. The genome is a circular mapping molecule 48,522 bp in size. All genes are on the same

strand, with the exception of a single tRNA gene (tRNA-Ala). The gray inner graph shows G/C content across the genome; the middle line showing 50% G/C. Genes are color-coded according to the predicted functional categories shown in the lower left.

Fig. S1.6.1a-h. Maximum likelihood phylogenetic trees of candidate genes / proteins derived by endosymbiotic gene transfer (EGT). Proteins encoded by genes found in the *Paramoeba pemaquidensis* (host) nuclear genome are highlighted by orange boxes, while homologs in the nuclear genome of the endosymbiont *Perkinsela* sp. (when present) are highlighted by blue boxes. Other amoebozoan and kinetoplastid sequences are highlighted with orange and blue text, respectively. RAxML Bootstrap values (100 replicates) are shown where ≥ 50%. Thick lines indicate Bayesian posterior probabilities ≥ 0.95. Scale bars show inferred number of amino acid substitutions per site.

Fig. S1.6.2. Phylogenomics pipeline for investigating endosymbiotic gene transfer (EGT). See supplemental text for details.

Fig. S1.6.3. Mitochondrial carrier signature sequences in *Paramoeba pemaquidensis* proteins derived by endosymbiotic gene transfer (EGT). **(a)** Alignment of a putative mitochondrial ADP/ATP translocase protein encoded in the *P. pemaquidensis* nuclear genome with two homologs found in *Perkinsela* sp. These sequences possess amino acid sequence motifs like mitochondrial carrier signature sequences found in other organisms (PX[D/E]XX[K/R]). **(b)** Alignment of two EGT-derived mitochondrial carrier proteins in *P. pemaquidensis* with a homolog in *Perkinsela* sp. These proteins also contain mitochondrial carrier signatures. Protein IDs are provided for each sequence.

Fig. S1.7.1. Mitochondrial proteome of *Perkinsela* sp. (a) Venn diagram showing the number of mitochondrial proteins predicted using TargetP, Predotar, and PredSL targeting peptide prediction tools. (b) Histogram showing the functional diversity of putative mitochondrial proteins identified based solely on N-terminal prediction tools and using an HMMER-based procedure guided by experimentally determined mitochondrial proteins in *Trypanosoma brucei brucei* TREU 927. The histogram shows only the

results of 107 proteins falling into prominent functional categories (minor / miscellaneous categories are not shown).

Fig. S2.5.1. Predicted metabolic map of the *Perkinsela* sp. glycosome-like organelle and associated pathways. In addition to the ability to import cytosolic proteins via a peroxin-based translocation machinery, the putative *Perkinsela* sp. glycosome is predicted to contain enzymes for glycolysis, various carbohydrate-metabolizing enzymes, as well as enzymes involved in nucleotide and amino acid metabolism. The *Perkinsela* sp. glycosome appears to lack conserved peroxisomal functions for fatty acid oxidation and detoxification of reactive oxygen species. Protein names in green indicate *Perkinsela* sp. components strongly predicted to be glycosome-targeted and with homologs in the *Trypanosoma brucei* glycosome[50]. Proteins in red are those known from other kinetoplastids to be potentially glycosome-targeted, but where evidence for targeting in *Perkinsela* sp. is lacking. Enzymes in gray are proteins that have thus far not been reported to be glycosome-localized in other organisms, as well as proteins with unknown function (UKFs). Superscripted numbers indicate presence of a predicted peroxisomal targeting signal 1 (1: PTS1) or 2 (2: PTS2). If the protein name/number is followed by a bracketed question mark (?), the targeting prediction is unclear. In cases where the protein name is followed by a question mark without brackets, the presence of the protein in *Perkinsela* sp. is unclear. Arrows show the direction of enzyme-catalyzed reactions and metabolite transport. Dashed lines or dashed arrows (black: very likely, gray: possible) represent putative transport routes without clear evidence for the presence of specific membrane translocators. For abbreviations of the protein names, see Table S2.5.2, which contains a detailed list of all putative glycosome-localized proteins, as well as proteins potentially connected to glycosome-associated metabolic pathways. Additional abbreviations: DUF: domain of unknown function, DHAP: dihydroxyacetone phosphate, PEP: phosphoenolpyruvate, Pyr: pyruvate, OXA: oxaloacetate, MAL: malate, FUM: fumarate, SUC: succinate, IMP: inosine monophosphate, SAM: S-Adenosyl methionine, SAH: -Adenosyl-L-homocysteine, HMGCoA: 3-hydroxy-3-methyl-glutaryl-coenzyme A, Mev: mevalonate.

Fig. S2.6.1. Transmission electron micrographs of the amoebozoan *Paramoeba pemaquidensis* CCAP 1560/4 and its kinetoplastid endosymbiont *Perkinsela* sp. **a.** Subcellular features of *P. pemaquidensis*, with the amoeba host nucleus (NP), host mitochondria (M), and nucleus-associated *Perkinsela* sp. endosymbiont (En) highlighted. Panel **a1** shows higher magnification of the host mitochondria with branched tubular cristae. Panel **a2** shows a putative endocytotic vesicle (Ve) within the endosymbiont, with what appears to be a single membrane (white arrow) and an inner glycoprotein surface (black arrow). For reference, the arrowhead points to the double membrane surrounding a nearby mitochondrion in the amoeba cytoplasm. Panel **a3** shows a higher magnification of the host nuclear envelope (Nm), while Panel **a4** highlights the plasma membrane of *P. pemaquidensis* with thin amorphous glycocalyx. **b.** Cross section showing microtubules (Mt) beneath the plasma membrane (black arrowhead) of the endosymbiont *Perkinsela* sp. The endosymbiont cytoplasm and host amoeba cytoplasm are labelled EnC and PaC, respectively. **c.** Longitudinal section of *Perkinsela* sp. microtubules (white arrows). Panel-specific scale bars are provided.

Fig. S2.7.1. Workflow for the identification of transporter protein genes in the *Perkinsela* sp. and *Paramoeba pemaquidensis* nuclear genomes.

Fig. S2.7.2. Classification of *Perkinsela* sp. transporter proteins and those of select organisms using TransportDB. *Perkinsela* sp. (red) was compared to *Leishmania major* Friedlin (black), *Trypanosoma brucei brucei* TREU927/4 GUTat10.2 (light blue), and *Trypanosoma cruzi* CL Brener TC3 (light red). (A) The percentage of predicted transporter proteins encoded by the *Perkinsela* sp. genome relative to the total number of protein-coding genes, compared to the same metric for other kinetoplastids. Transporters were first split into the following superfamilies: ATP-dependent, Ion Channels, Secondary transporters, and Unclassified. Each superfamily was then broken down into families. (B) Unclassified superfamily, (C) Secondary transporter superfamily, (D) Ion channels superfamily, (E) ATP-dependent superfamily.

Fig. S2.7.3. Workflow for prediction and analysis of *Perkinsela* sp. secreted proteins. (1) Proteins secreted into the host cytosol, (2) proteins present in the plasma membrane (with transmembrane domain) and (3) proteins present in the plasma membrane (with GPI anchor).

Fig. S2.8.1. KEGG pathway[51] showing cell cycle-associated proteins. Proteins present in both *Perkinsela* sp. (endosymbiont) and *Paramoeba pemaquidensis* (host) are highlighted blue, those only in *P. pemaquidensis* are shown in red, and those only in *Perkinsela* sp. are in green.

Fig. S3.1.1a. KEGG pathway[51] showing glutathione / trypanothione metabolism. Proteins with clear homologs in the endosymbiont *Perkinsela* sp. are highlighted green, while those encoded in the nuclear genome of *Paramoeba pemaquidensis* are highlighted red. Green text corresponds to gene / enzyme names in bacteria.

Fig. S3.1.1b. KEGG pathway[51] showing ubiquinone / terpenoid-quinone metabolism. Proteins with clear homologs in the endosymbiont *Perkinsela* sp. are highlighted green, while those encoded in the nuclear genome of *Paramoeba pemaquidensis* are highlighted red. Green text corresponds to gene / enzyme names in bacteria.

Fig. S3.1.1c. KEGG pathway[51] showing arginine and proline metabolism. Proteins with clear homologs in the endosymbiont *Perkinsela* sp. are highlighted green, while those encoded in the nuclear genome of *Paramoeba pemaquidensis* are highlighted red.

Fig. S3.1.1d. KEGG pathway[51] showing fatty acid metabolism. Proteins with clear homologs in the endosymbiont *Perkinsela* sp. are highlighted green, while those encoded in the nuclear genome of *Paramoeba pemaquidensis* are highlighted red.

Fig. S3.1.1e. KEGG pathway[51] showing purine metabolism. Proteins with clear homologs in the endosymbiont *Perkinsela* sp. are highlighted green, while those

encoded in the nuclear genome of *Paramoeba pemaquidensis* are highlighted red. Green text corresponds to gene / enzyme names in bacteria.

Fig. S3.1.1f. KEGG pathway[51] showing terpenoid biosynthesis. Proteins with clear homologs in the endosymbiont *Perkinsela* sp. are highlighted green, while those encoded in the nuclear genome of *Paramoeba pemaquidensis* are highlighted red.

Fig. S3.1.1g. KEGG pathway[51] showing the citrate cycle (tricarboxylic acid cycle). Proteins with clear homologs in the endosymbiont *Perkinsela* sp. are highlighted green, while those encoded in the nuclear genome of *Paramoeba pemaquidensis* are highlighted red.

Fig. S3.11.1. KEGG pathway[51] showing autophagy proteins. Proteins with clear homologs identified in the nuclear genome of *Paramoeba pemaquidensis* are highlighted red. No autophagy-associated proteins were identified in the nuclear genome of *Perkinsela* sp.

Fig. S3.12.1. KEGG pathway[51] showing endocytosis-associated proteins. Proteins with clear homologs in *Perkinsela* sp. are highlighted green, while those found in the nuclear genome of *Paramoeba pemaquidensis* are shown in red.

Fig. S3.13.1. KEGG pathway[51] showing lysosome-associated proteins. Proteins with clear homologs in the endosymbiont *Perkinsela* sp. are highlighted green, while those encoded in the nuclear genome of *Paramoeba pemaquidensis* are highlighted red.

Fig. S4. Density gradient centrifugation and quantification of total DNA fractions isolated from *Paramoeba pemaquidensis* CCAP 1560/4 and its endosymbiont *Perkinsela* sp. **(a)** UV light exposure of Hoechst dye-cesium chloride density gradient centrifugation tube after ultracentrifugation at 40,000 *g* for 67 hours. **(b-d)** Ethidium bromide stained agarose gel electrophoresis of semi-quantitative PCR amplicons generated using genome-specific primers and DNA from the three fractions shown in (a). **(e)** PCR

amplification of four different concentrations of plasmid DNA used as a standard. Abbreviations: M= host mitochondrion (*cox1* gene), IRE=*Perkinsela* sp. (*Ichthyobodo*-related endosymbiont) (*rpb1* gene), host=*Paramoeba pemiquidensis* (*rpb1* gene).

Fig. S5. RNA-seq-based *in silico* profiling of genomic scaffolds from *Paramoeba pemaquidensis* CCAP 1560/4 and its endosymbiont *Perkinsela* sp. RNA-seq reads from the spliced leader (SL)-amplified RNA-seq library were mapped to each of the genomic scaffolds, as were the RNA-seq reads derived from the total RNA library. A SL RNA / total RNA ratio was then calculated for each scaffold based on the total number of mapped RNA-seq reads from each library. **(a)** For each scaffold, the SL RNA / total RNA ratio was plotted against scaffold sequence depth coverage. **(b)** Plot showing SL RNA / total RNA ratio relative to scaffold G/C content. Colored shapes correspond to scaffolds flagged as being of endosymbiont nuclear, host nuclear, bacterial or kDNA origin (including, but not limited to, phylogenetic analyses carried out by David et al.[12]). Note that these analyses were not seen as definitive, but simply a preliminary assessment of the characteristics defining scaffolds of different genomic origins.

Fig. S6. Summary of bioinformatics pipeline used to investigate peroxisome- / glycosome-associated proteins in *Perkinsela* sp. and its host, *Paramoeba pemaquidensis*.

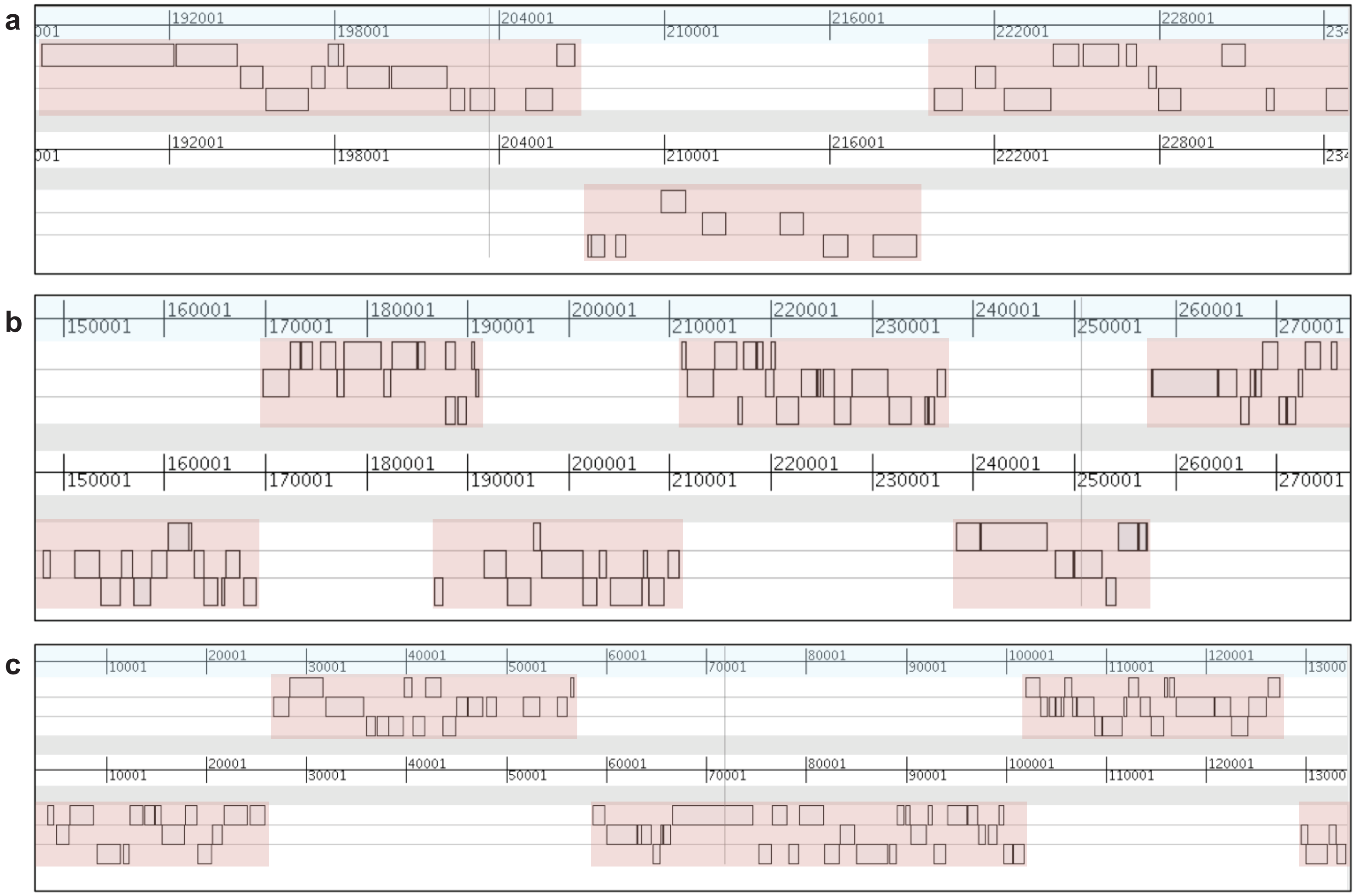

d

	Minimum gene block size			
	**≥1**	**≥2**	**≥3**	**≥4**
**Divergent (- +)**	738 bp	707 bp	665 bp	671 bp
**Convergent (+ -)**	1,218 bp	1,302 bp	1,372 bp	1,381 bp

Fig. S1.1. Strand polarity in the *Perkinsela* sp. nuclear genome.

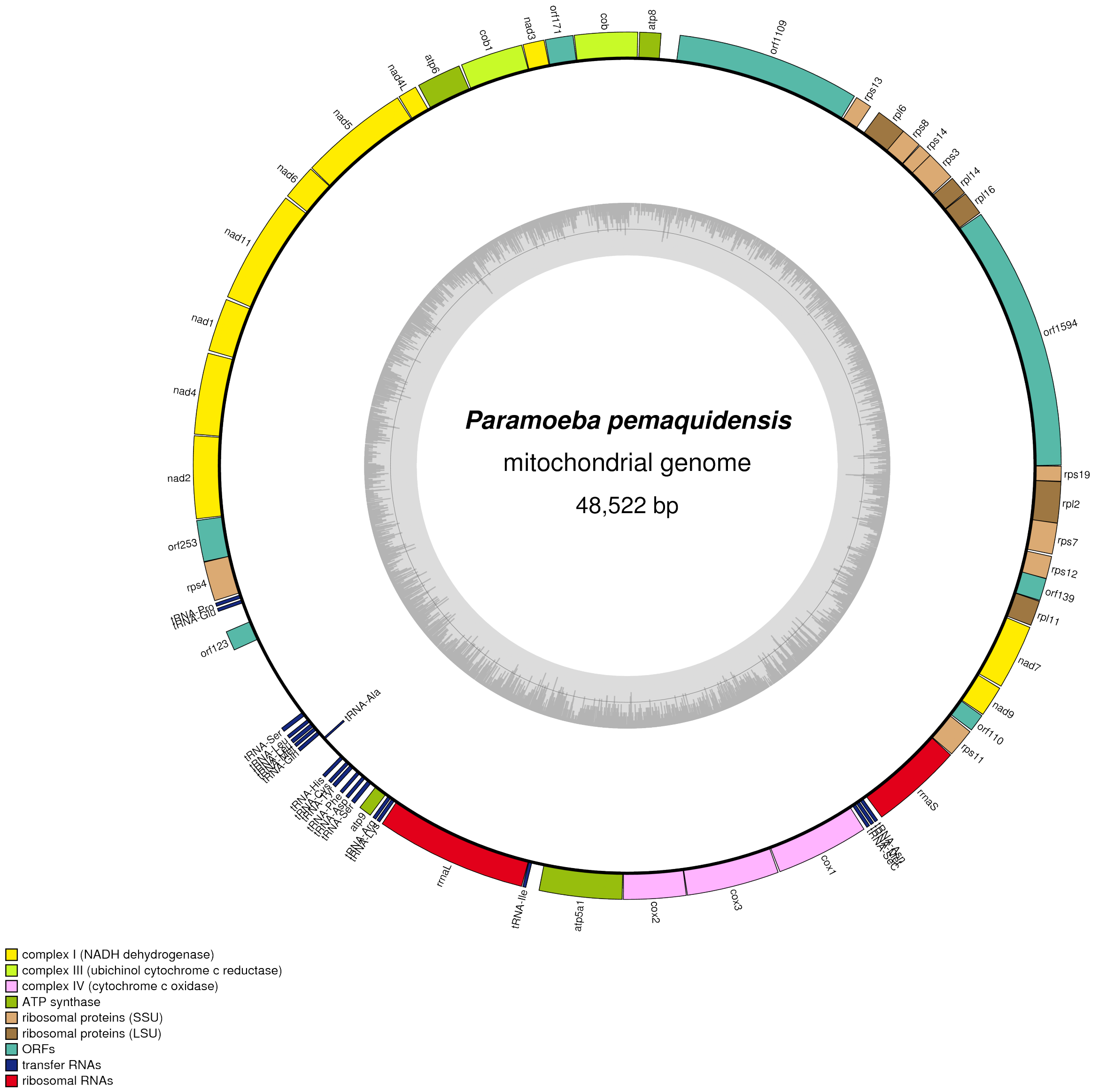

Fig. S1.5.1. Mitochondrial genome of *Paramoeba pemaquidensis* CCAP 1560/4.

a **Tree ID: 8307**
Query protein: NPAc7281A
Putative function: Peptidase M20 protein

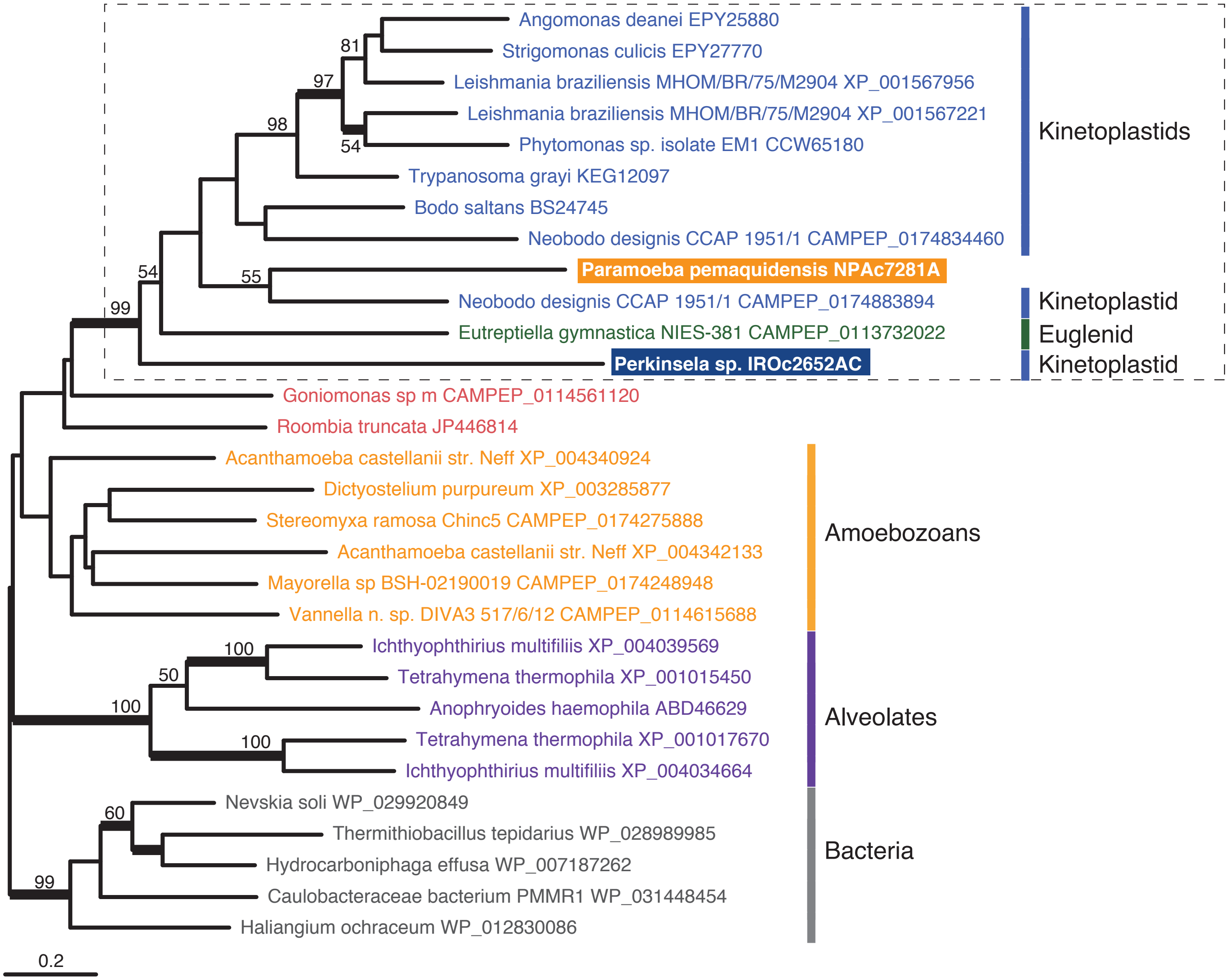

Figure S1.6.1. (a-h) Maximum likelihood phylogenetic trees of candidate genes / proteins derived by endosymbiotic gene transfer (EGT).

b **Tree ID: 4149**
Query protein: NPAc3631A
Putative function: Mitochondrial ADP/ATP translocase

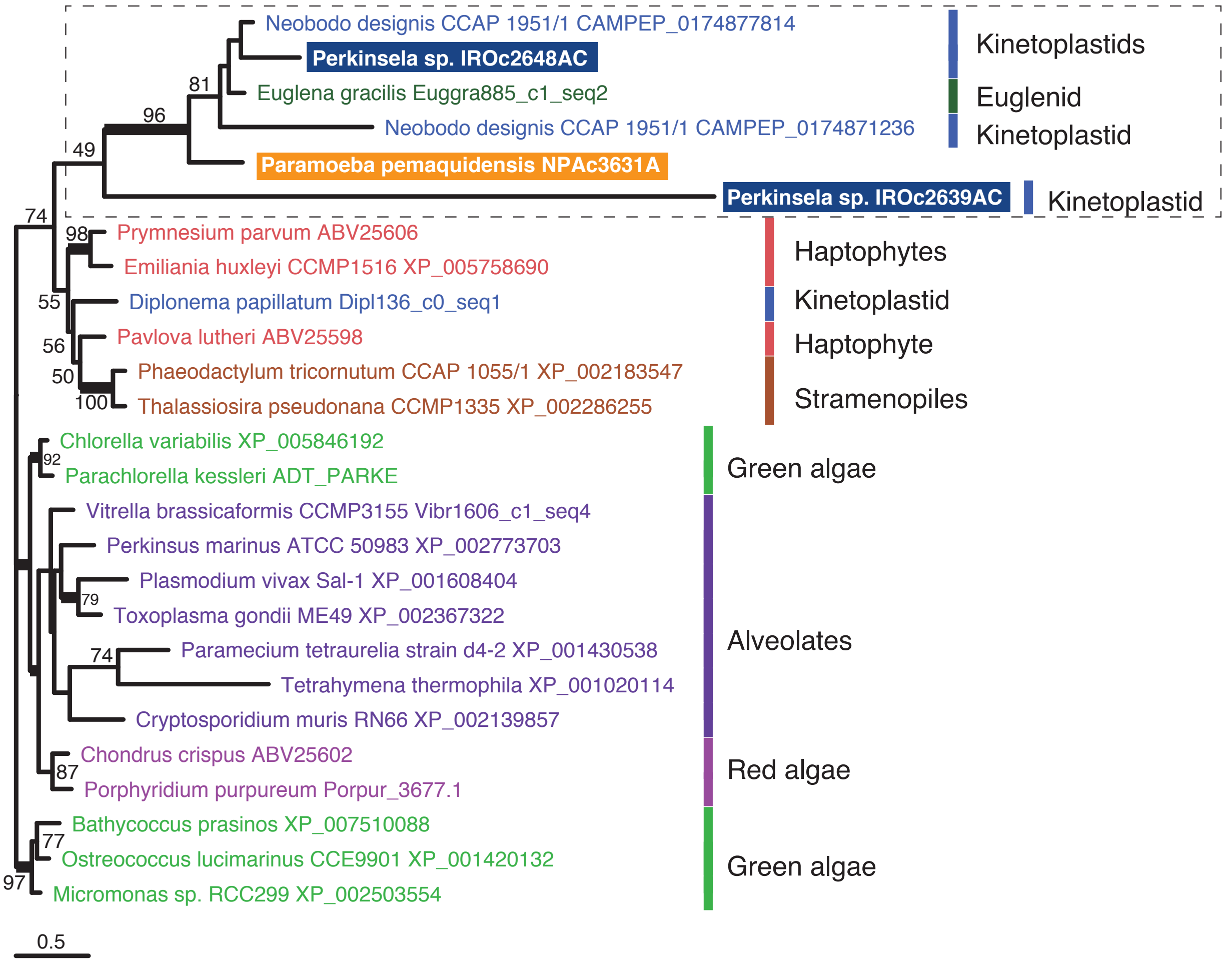

Figure S1.6.1. (a-h) Maximum likelihood phylogenetic trees of candidate genes / proteins derived by endosymbiotic gene transfer (EGT).

c

Tree ID: 7855
Query proteins: NPAc7727A & NPAc6887A
Putative function: Mitochondrial carrier protein

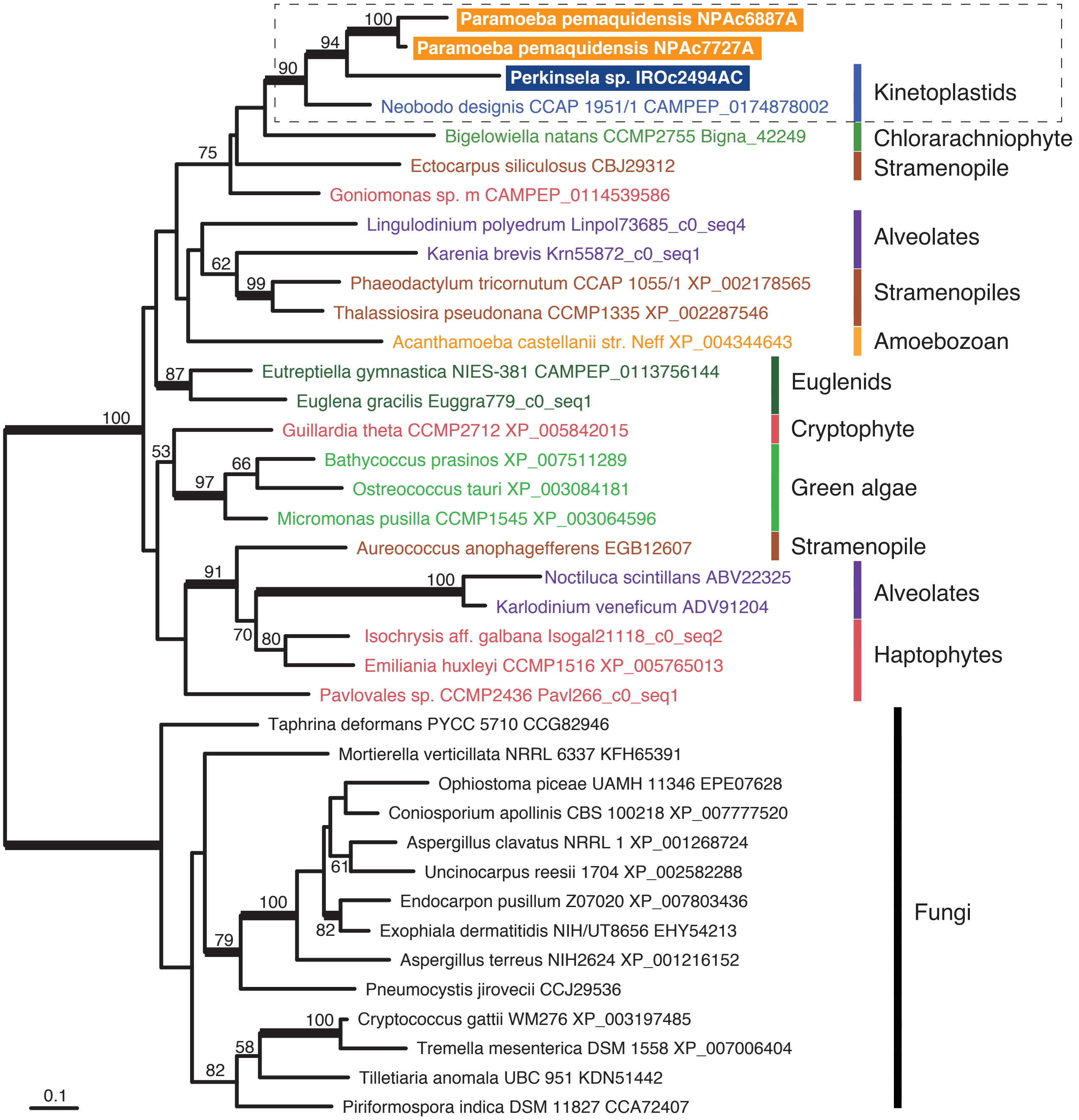

Figure S1.6.1. (a-h) Maximum likelihood phylogenetic trees of candidate genes / proteins derived by endosymbiotic gene transfer (EGT).

d **Tree ID: 8442**
Query protein: NPAc7401A
Putative function: Hybrid cluster protein (Hydroxylamine reductase)

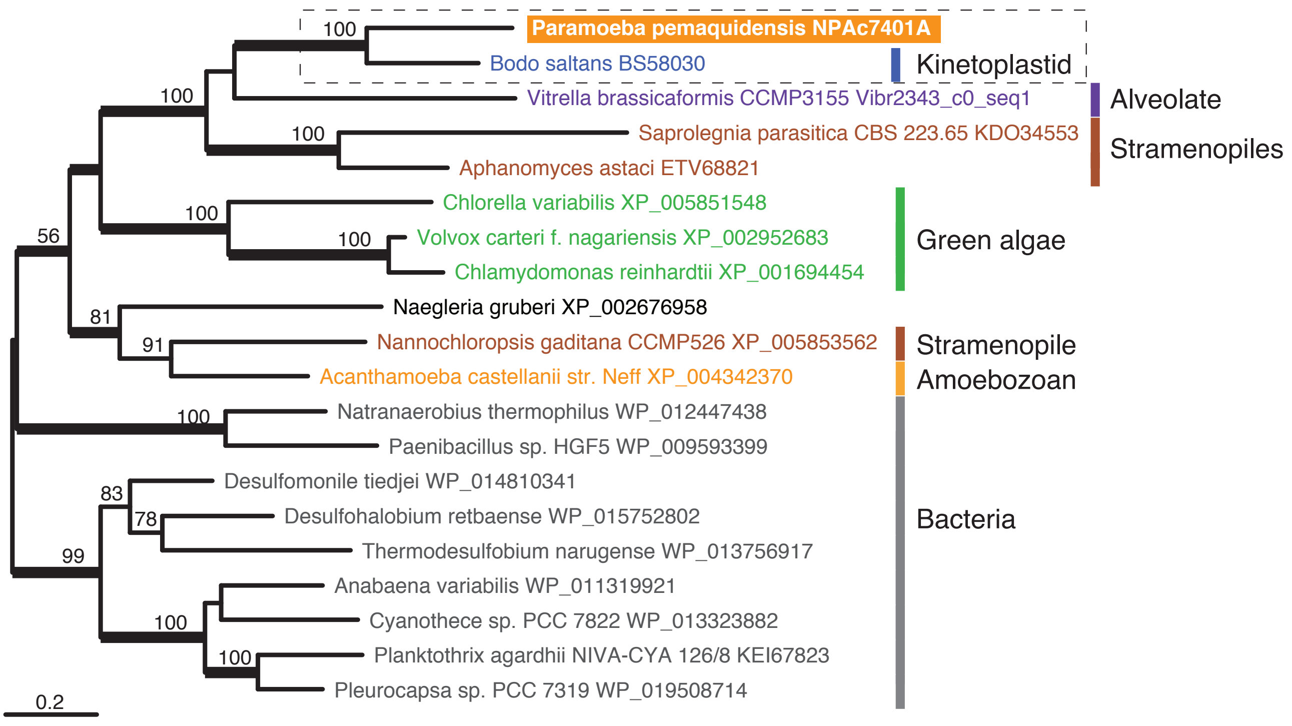

e **Tree ID: 10509**
Query protein: NPAc9246Abruce_1
Putative function: Mitochondrial aspartate aminotransferase

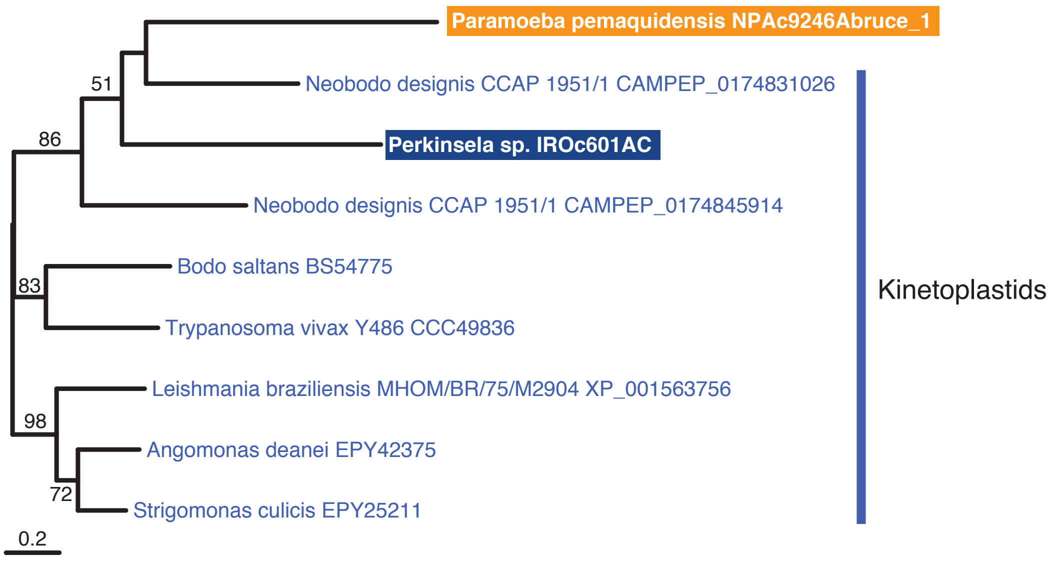

Figure S1.6.1. (a-h) Maximum likelihood phylogenetic trees of candidate genes / proteins derived by endosymbiotic gene transfer (EGT).

f **Tree ID: 2330**
Query protein: NPAc2055A
Putative function: Alkene reductase-like protein

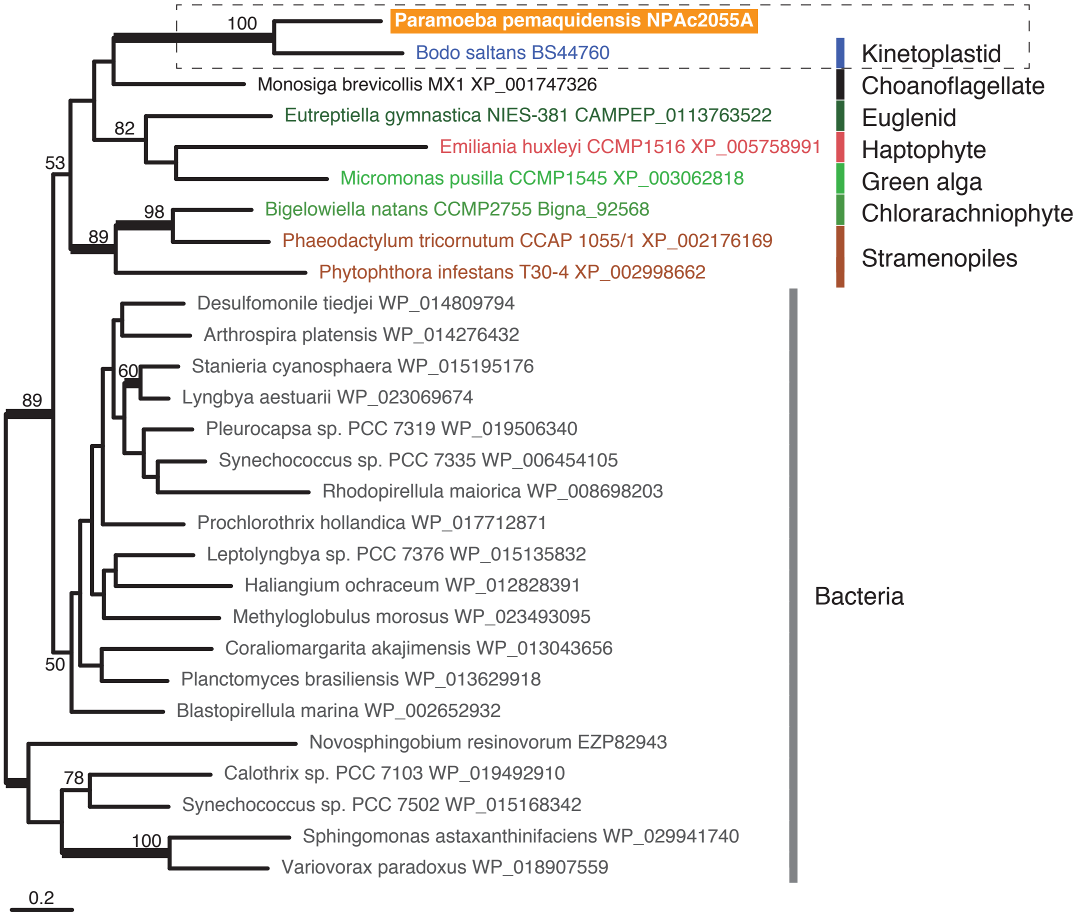

g **Tree ID: 11325**
Query protein: NPAc9974A
Putative function: Retrotransposon hotspot-like protein

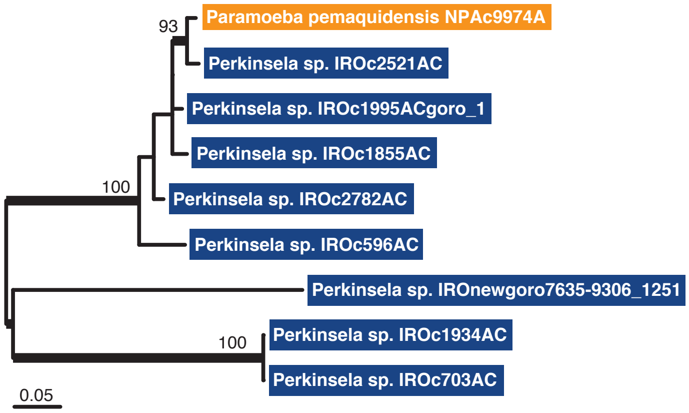

h **Tree ID: 11133**
Query protein: NPAc9801A
Putative function: unknown

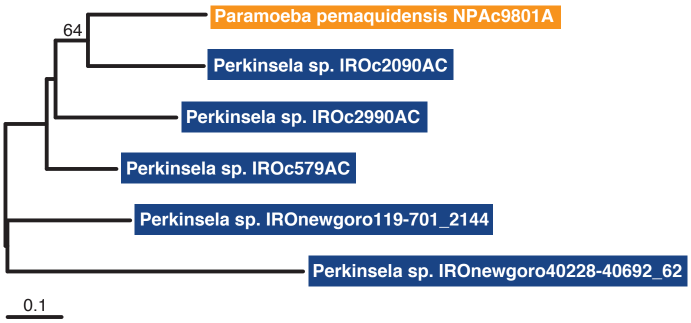

Figure S1.6.1. (a-h) Maximum likelihood phylogenetic trees of candidate genes / proteins derived by endosymbiotic gene transfer (EGT).

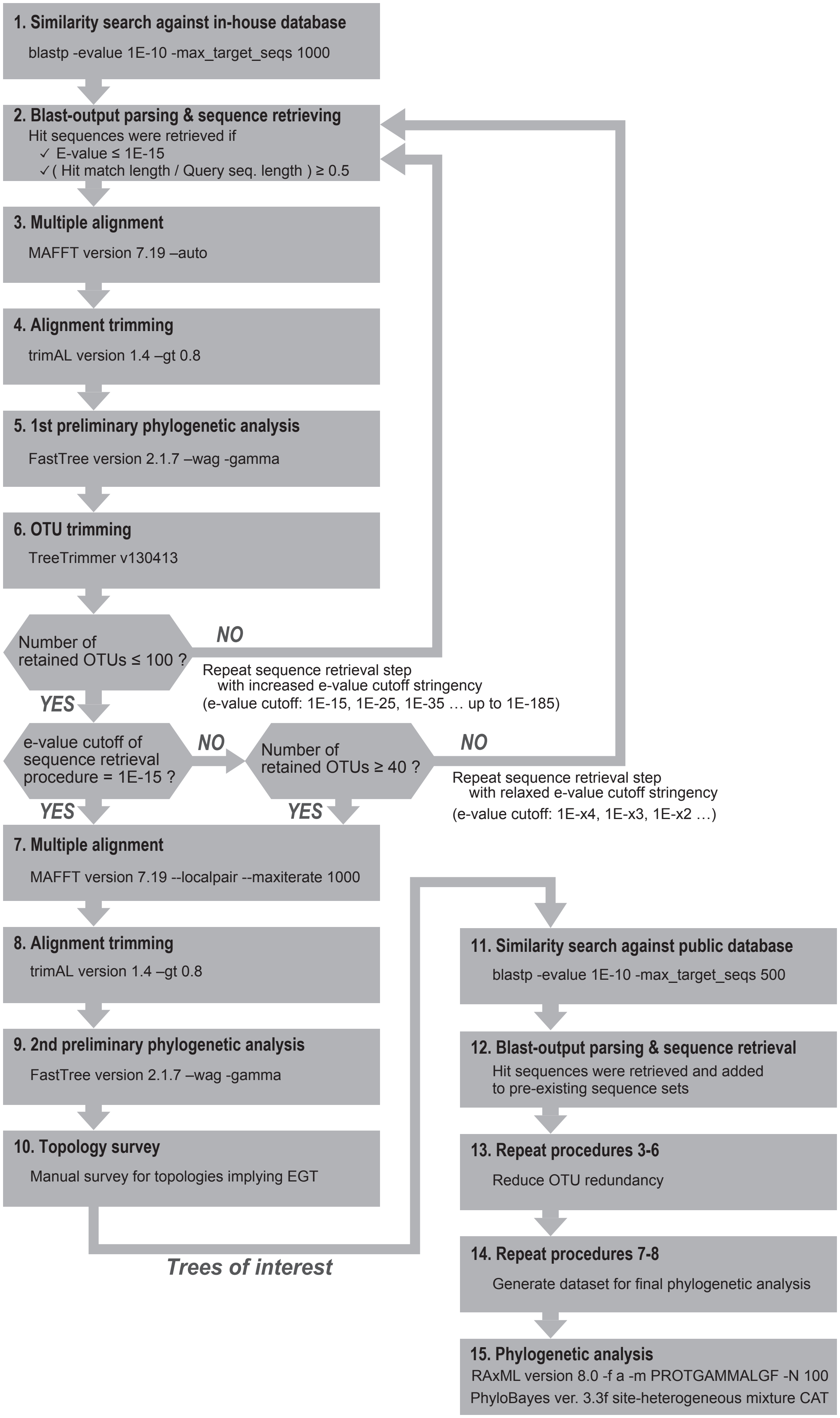

Fig. S1.6.2. Phylogenomics pipeline for investigating endosymbiotic gene transfer (EGT).

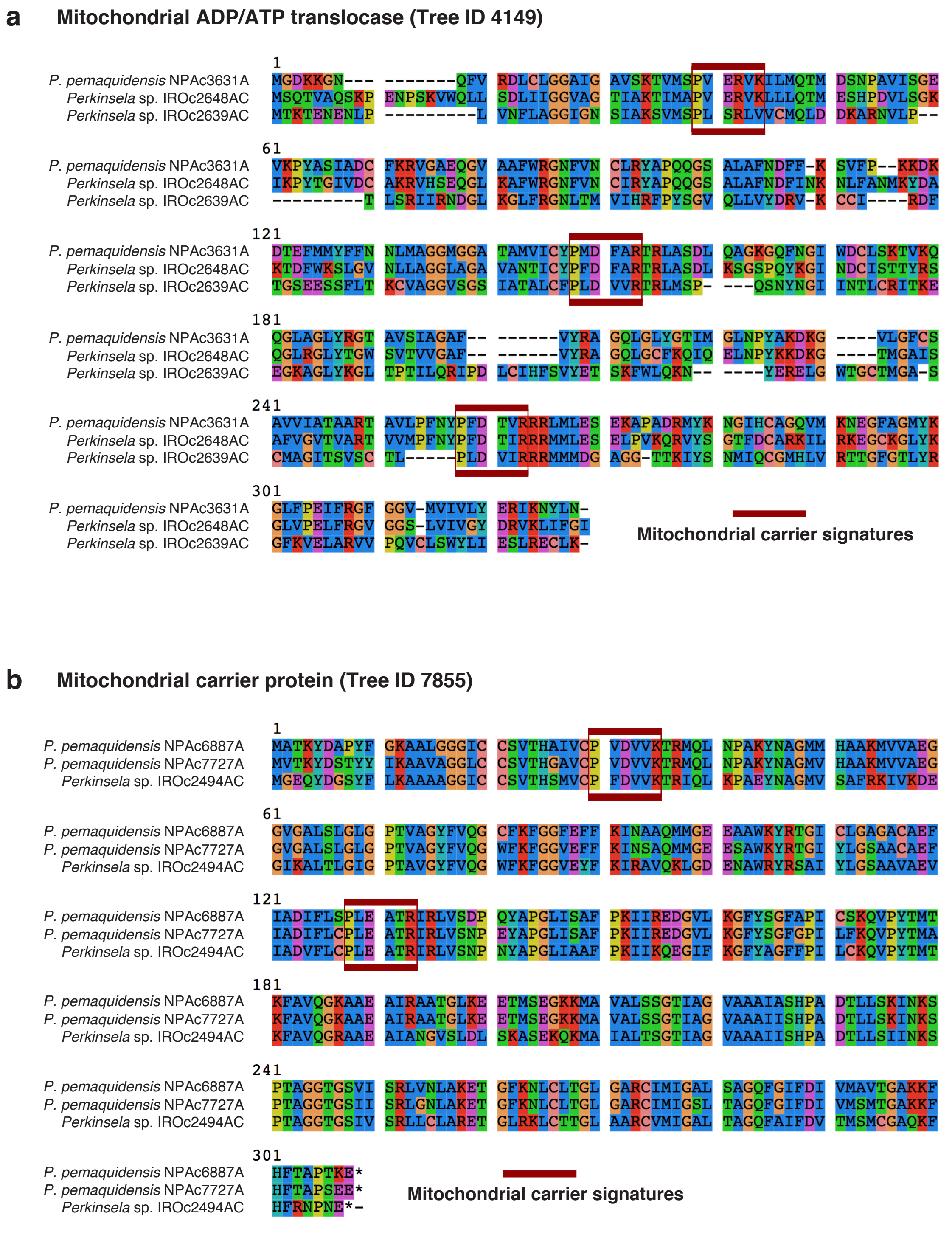

Figure. S1.6.3. Mitochondrial carrier signature sequences in *Paramoeba pemaquidensis* proteins derived by endosymbiotic gene transfer (EGT).

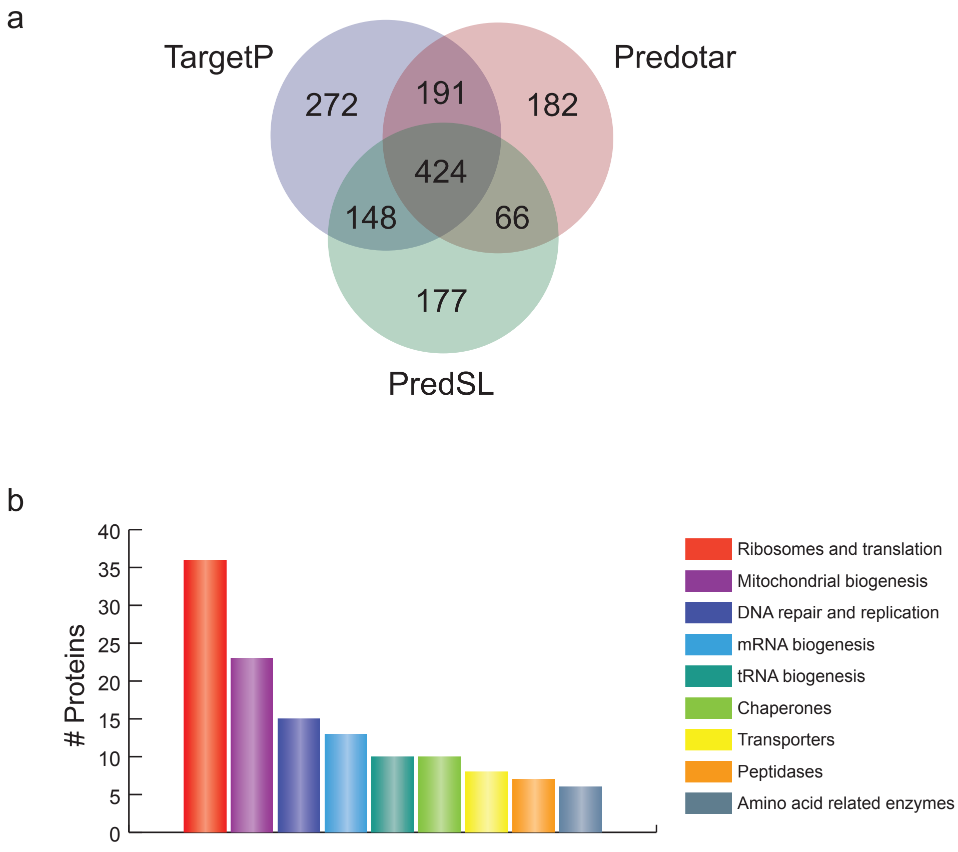

Figure. S1.7.1. Mitochondrial proteome of *Perkinsela* sp.

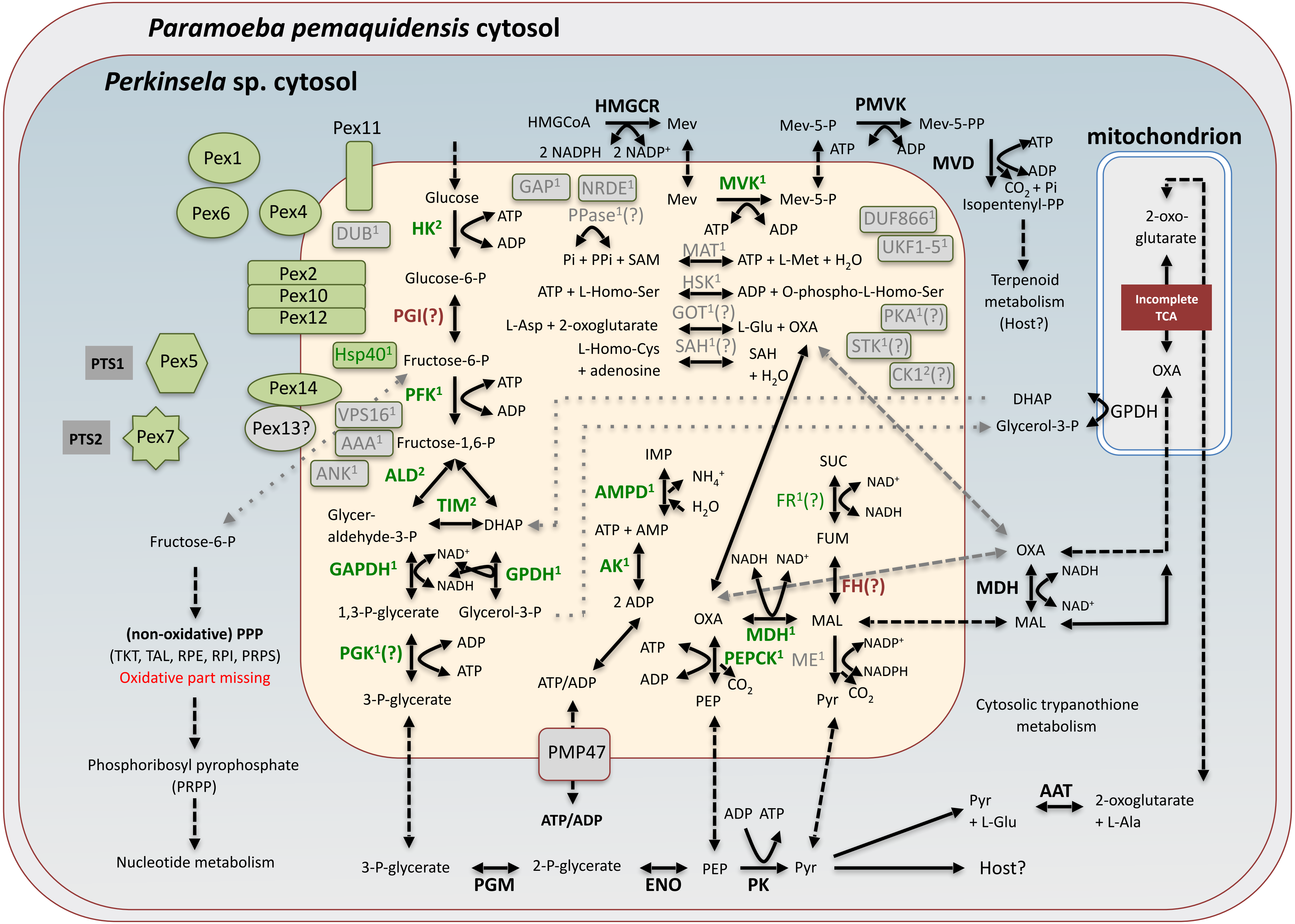

Supp. Fig. S2.5.1. Predicted metabolic map of the *Perkinsela* sp. glycosome-like organelle and associated pathways.

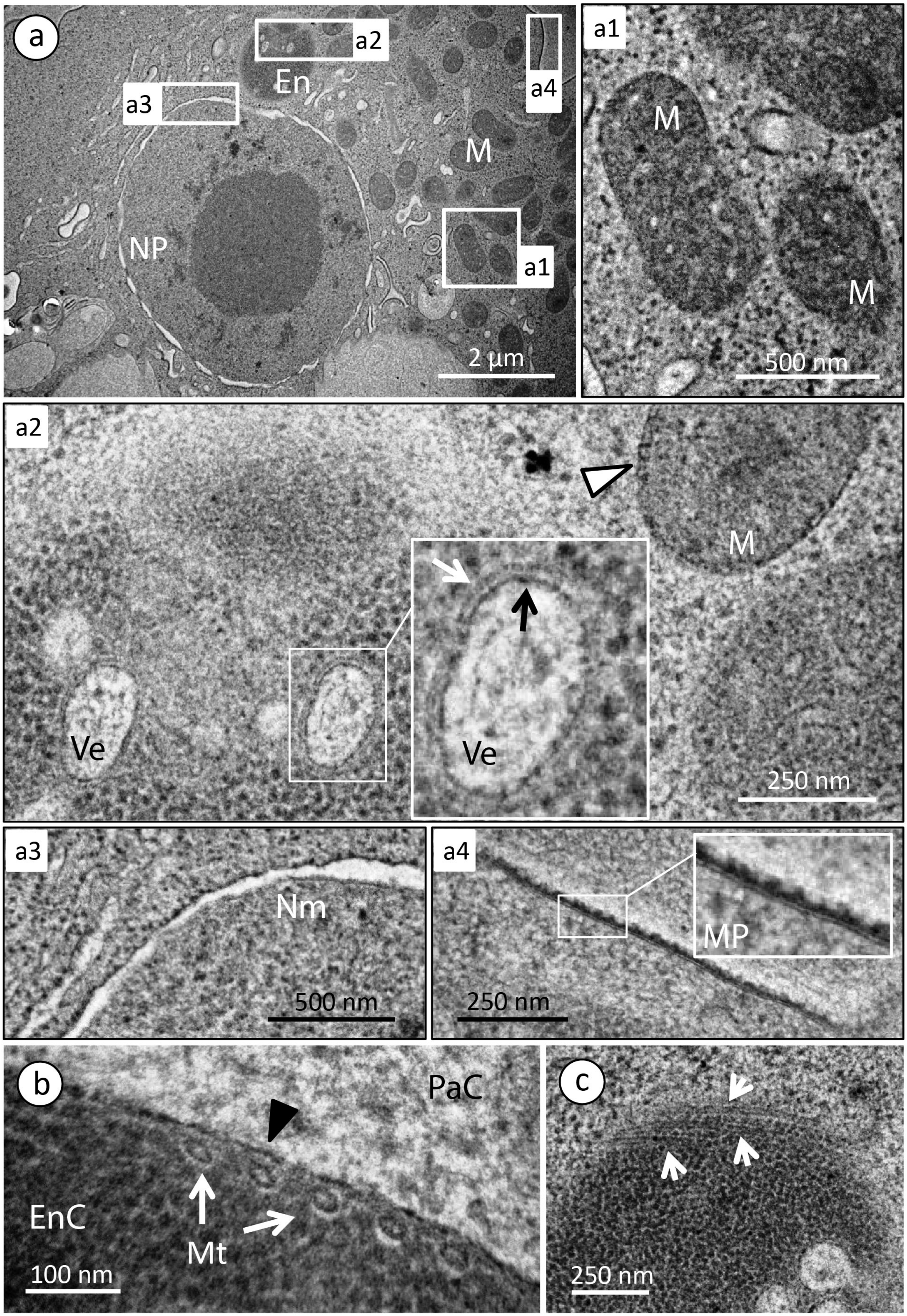

Figure. S2.6.1. Transmission electron micrographs of the amoebozoan *Paramoeba pemaquidensis* CCAP 1560/4 and its kinetoplastid endosymbiont *Perkinsela* sp.

Fig S2.7.1: Workflow for the identification of transporter protein genes in the *Perkinsela* sp. and *Paramoeba pemaquidensis* nuclear genomes.

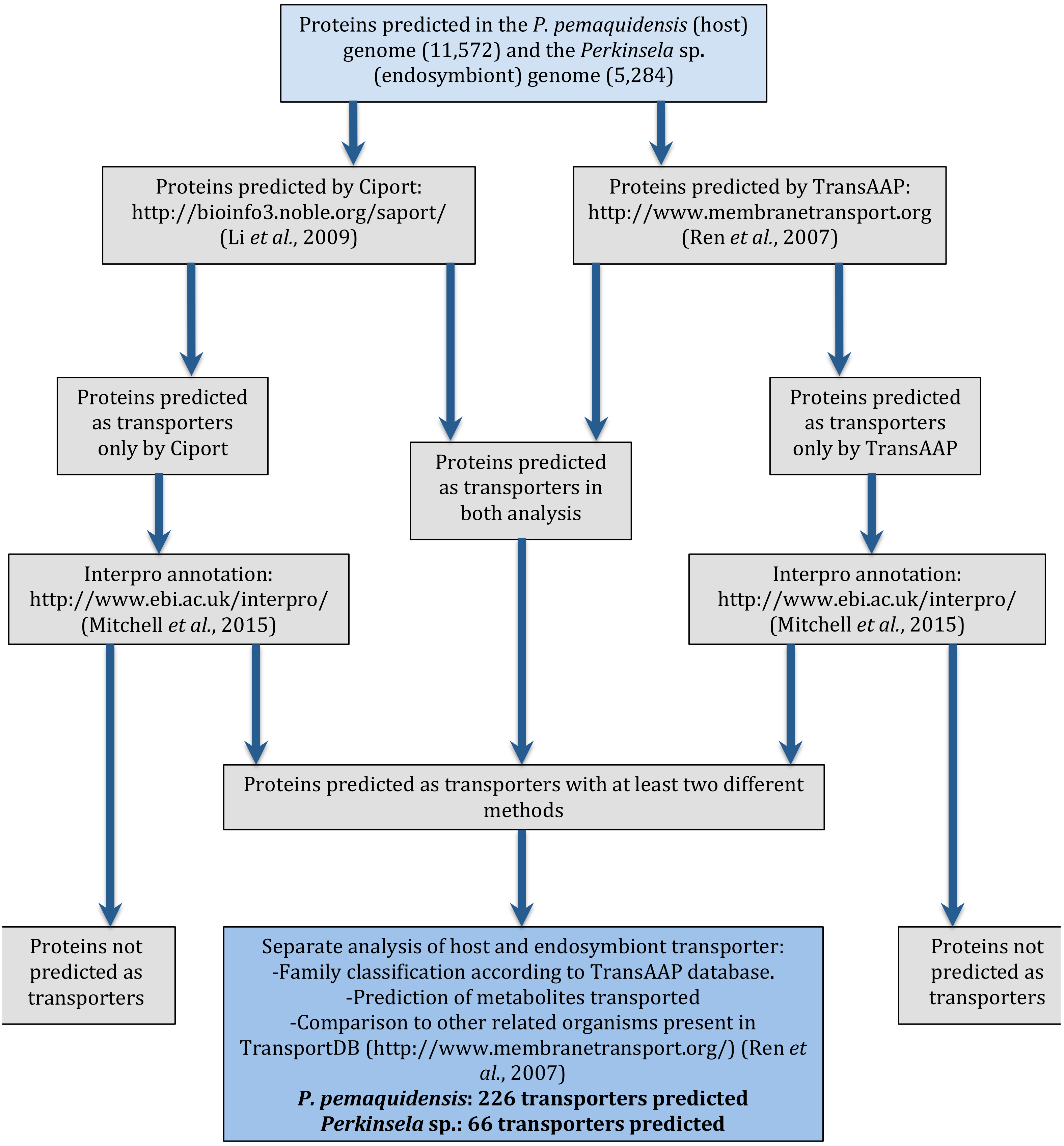

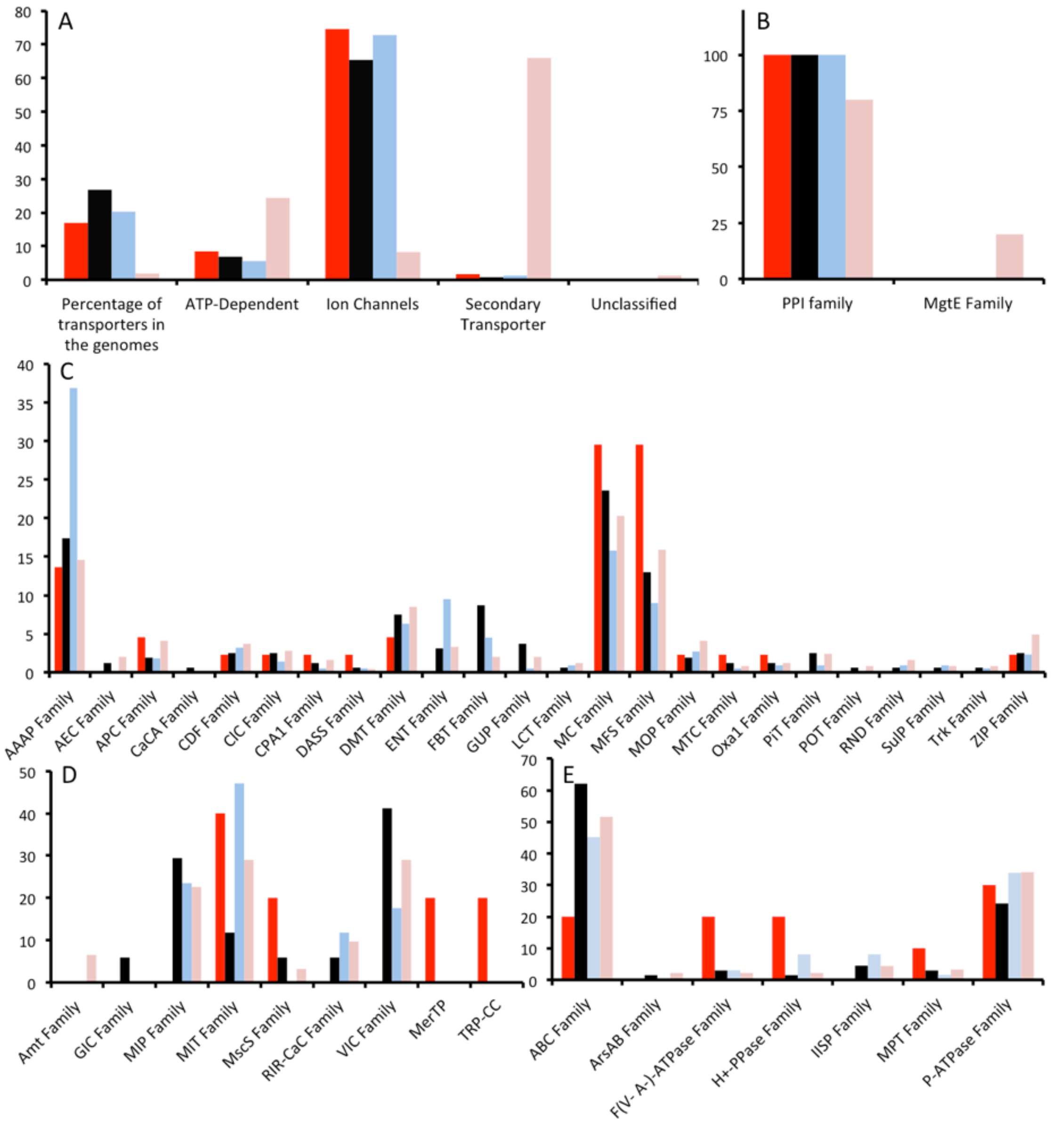

Figure. S2.7.2. Classification of *Perkinsela*. sp. transporter proteins and those of select organisms using TransportDB. (See text for full figure legend).

Fig. S.2.7.3. Workflow for prediction and analysis of *Perkinsela* sp. secreted proteins. (1) Proteins secreted into the host cytosol, (2) proteins present in the plasma membrane (with transmembrane domain) and (3) proteins present in the plasma membrane (with GPI anchor).

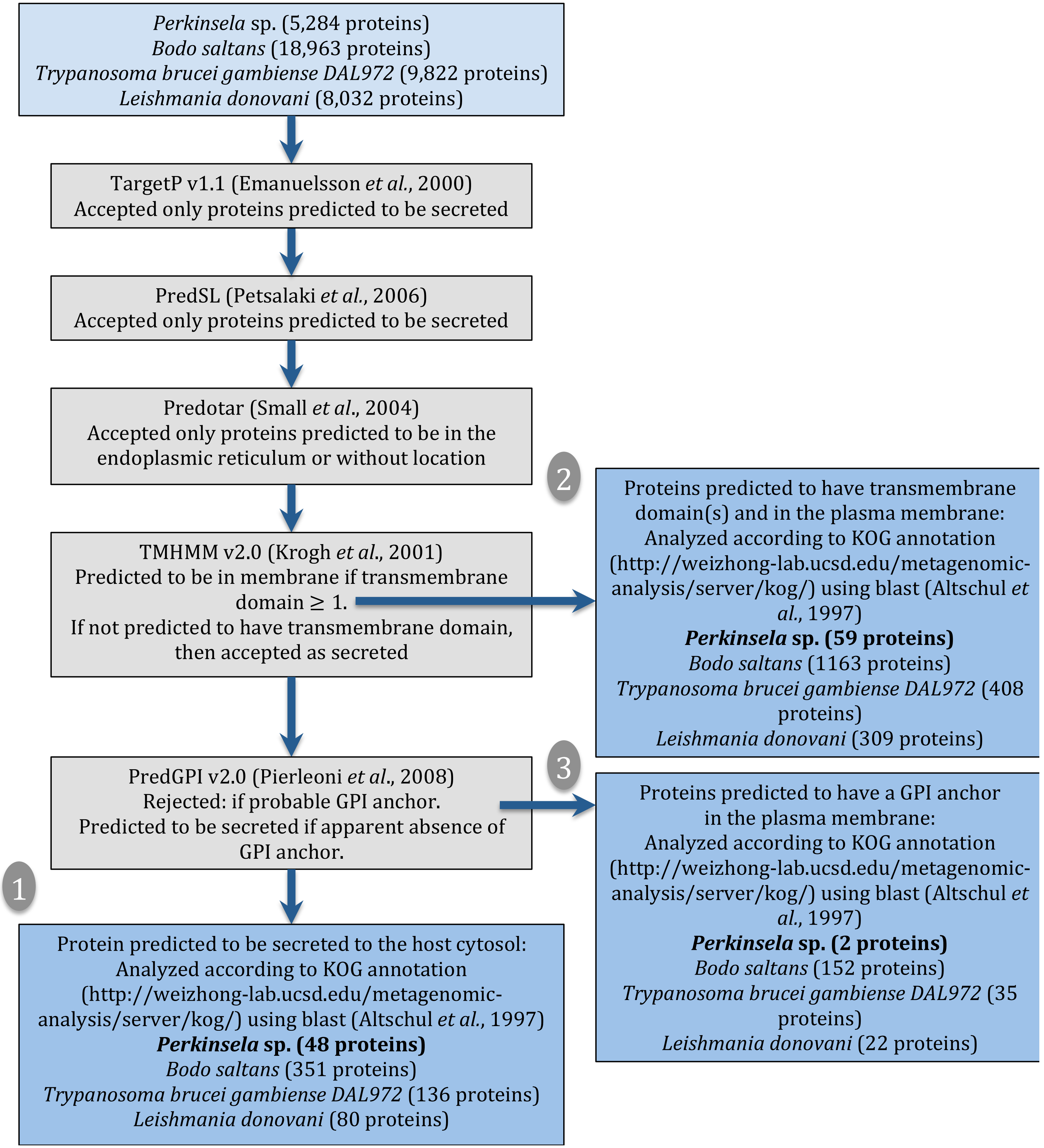

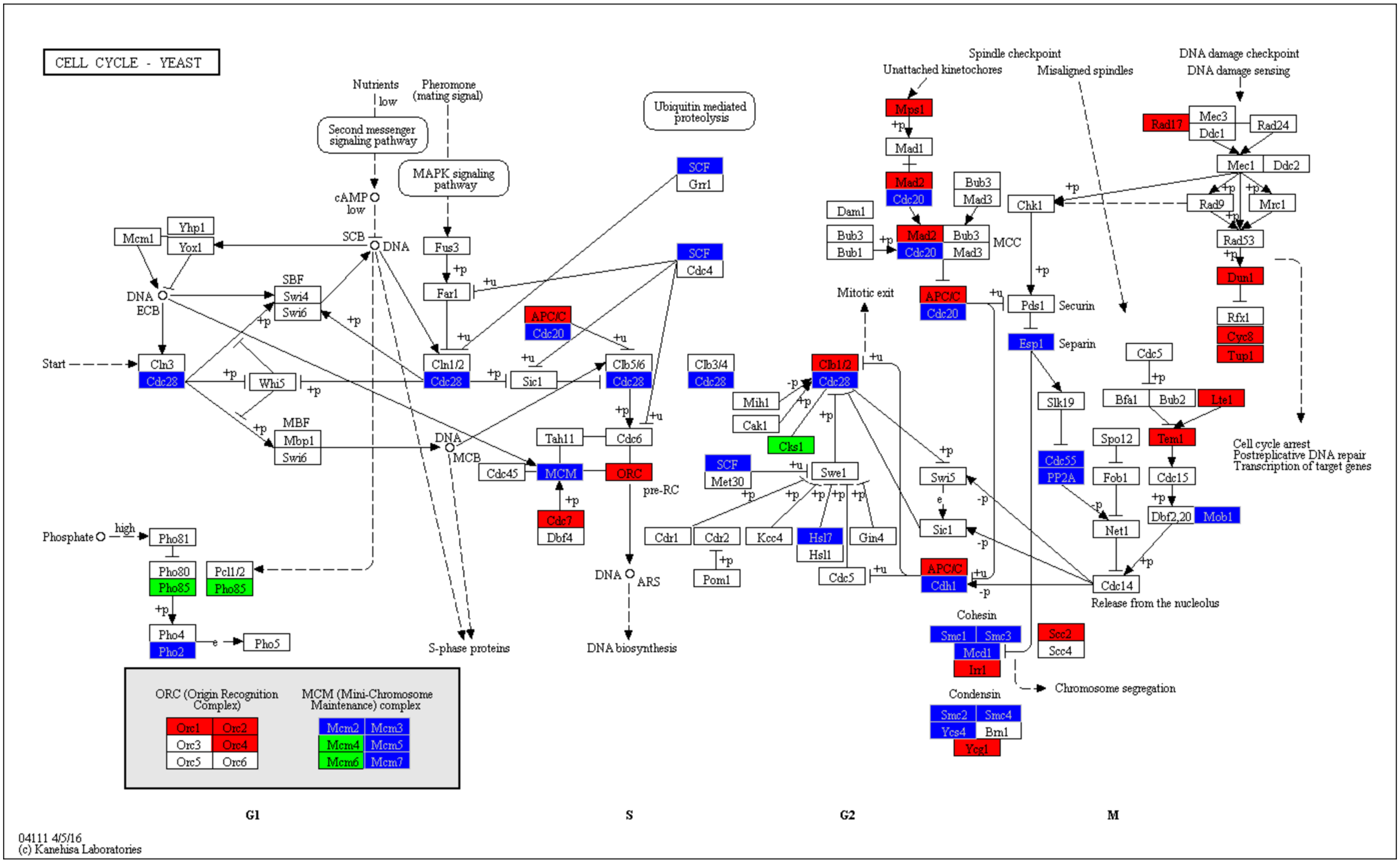

Figure. S2.8.1. KEGG pathway showing cell cycle-associated proteins. Proteins present in both *Perkinsela*. sp. (endosymbiont) and *Paramoeba pemaquidensis* (host) are highlighted blue, those only in *P. pemaquidensis* are shown in red, and those only in *Perkinsela*. sp. are in green.

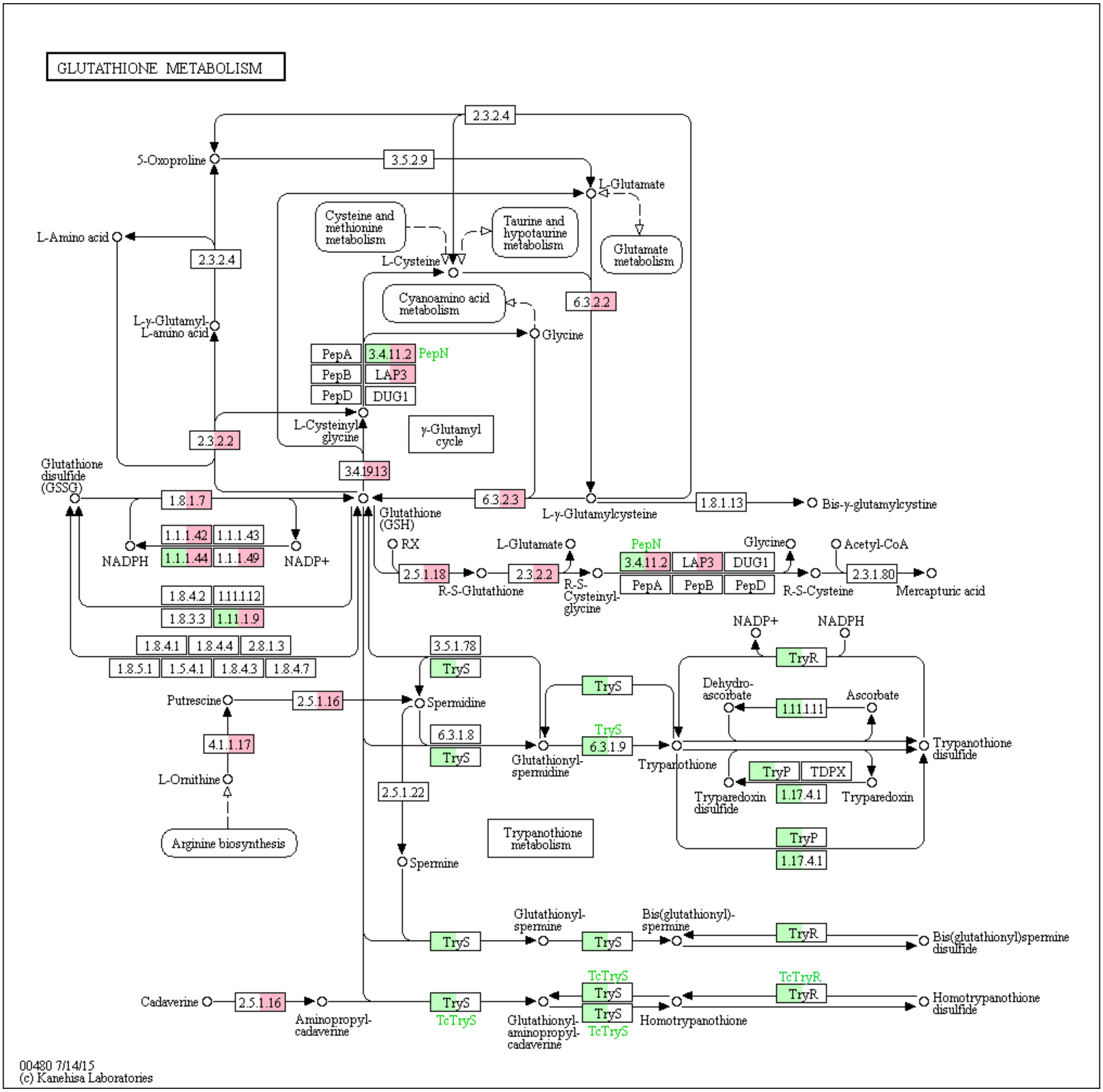

Figure. S3.1.1a. KEGG pathway showing glutathione / trypanothione metabolism. Proteins with clear homologs in the endosymbiont *Perkinsela*. sp. are highlighted green, while those encoded in the nuclear genome of the host *Paramoeba pemaquidensis* are highlighted red. Green text corresponds to gene / enzyme names in bacteria.

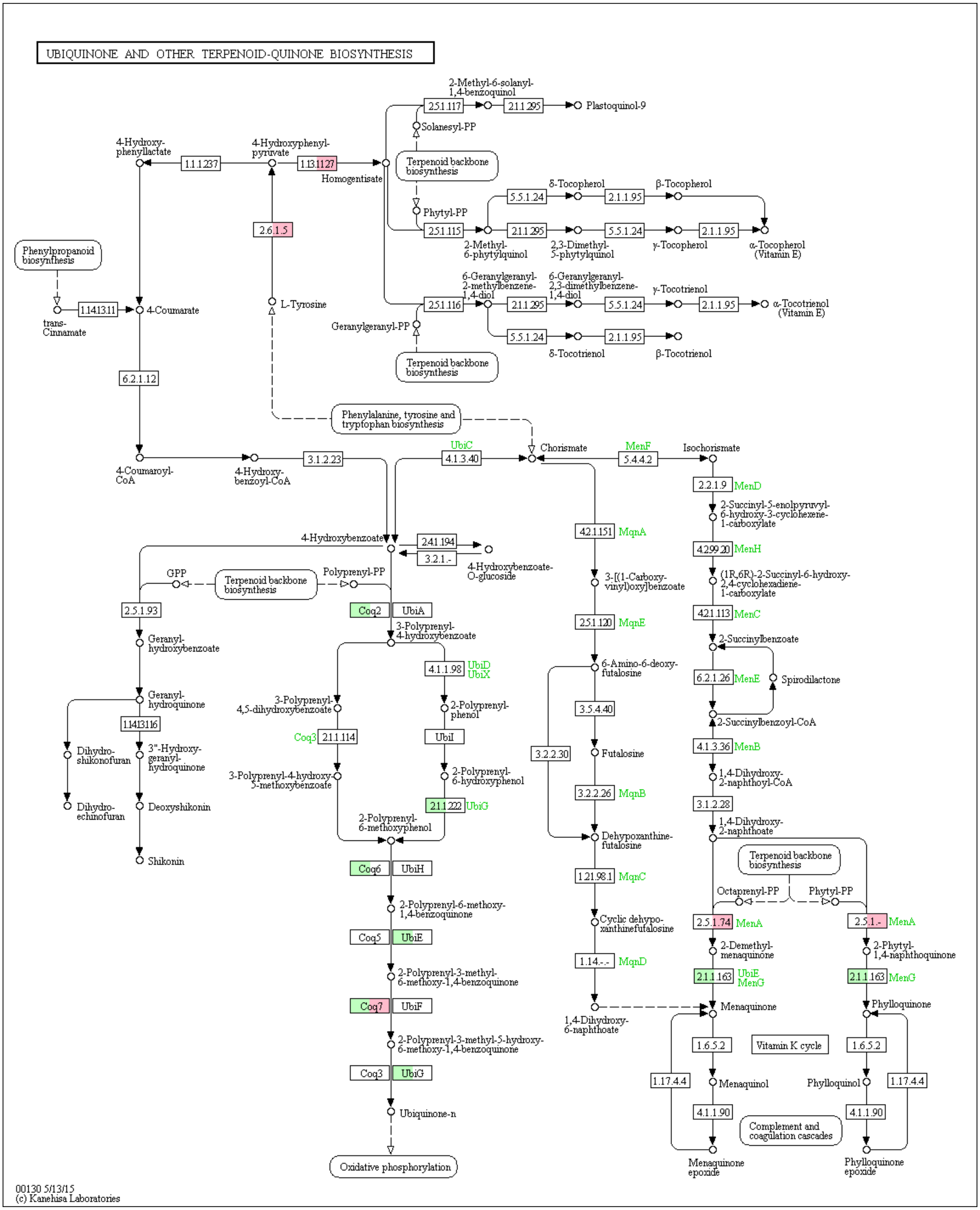

Figure. S3.1.1b. KEGG pathway showing ubiquinone / terpenoid-quinone metabolism. Proteins with clear homologs in the endosymbiont *Perkinsela*. sp. are highlighted green, while those encoded in the nuclear genome of the host *Paramoeba pemaquidensis* are highlighted red. Green text corresponds to gene / enzyme names in bacteria.

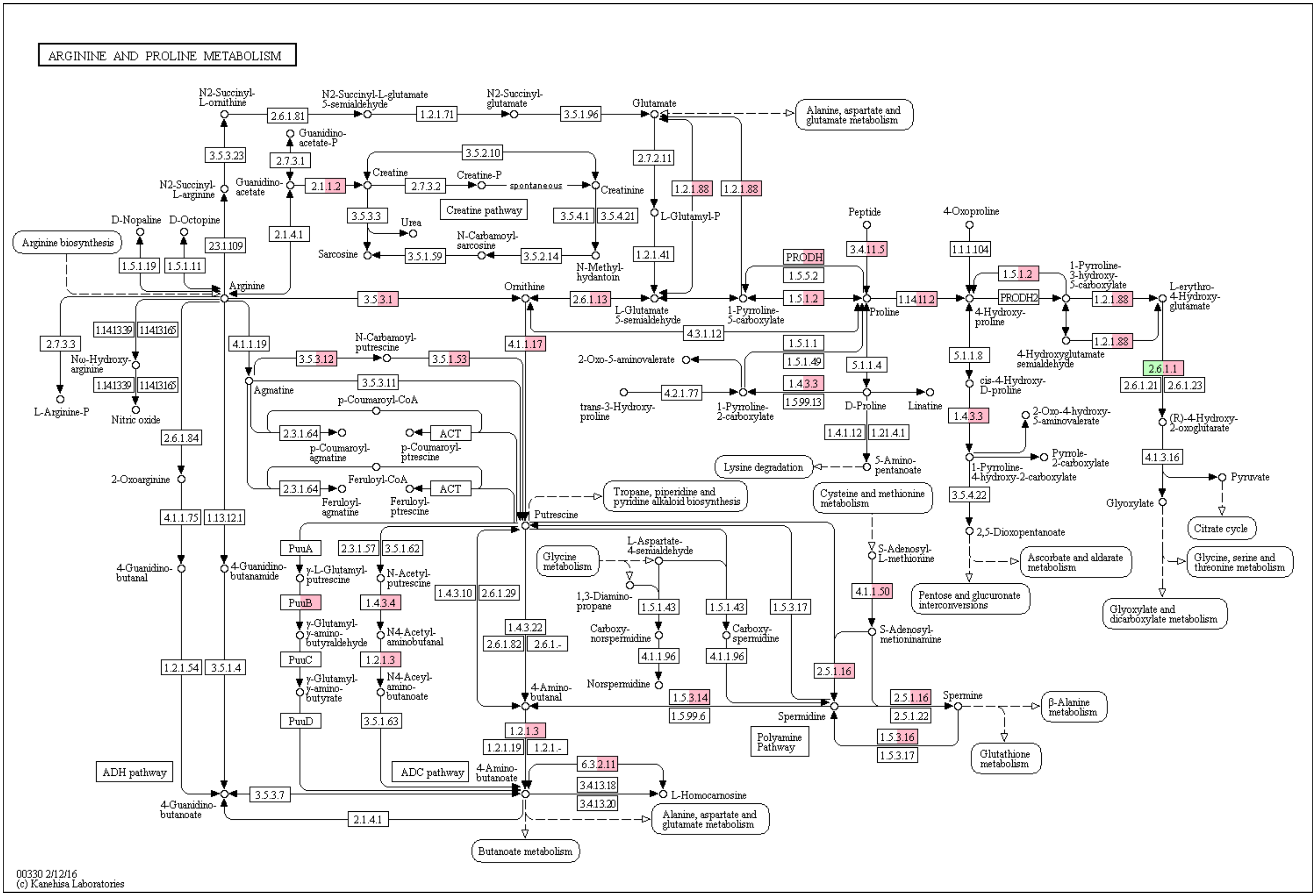

Figure. S3.1.1c. KEGG pathway showing arginine and proline metabolism. Proteins with clear homologs in the endosymbiont *Perkinsela*. sp. are highlighted green, while those encoded in the nuclear genome of the host *Paramoeba pemaquidensis* are highlighted red.

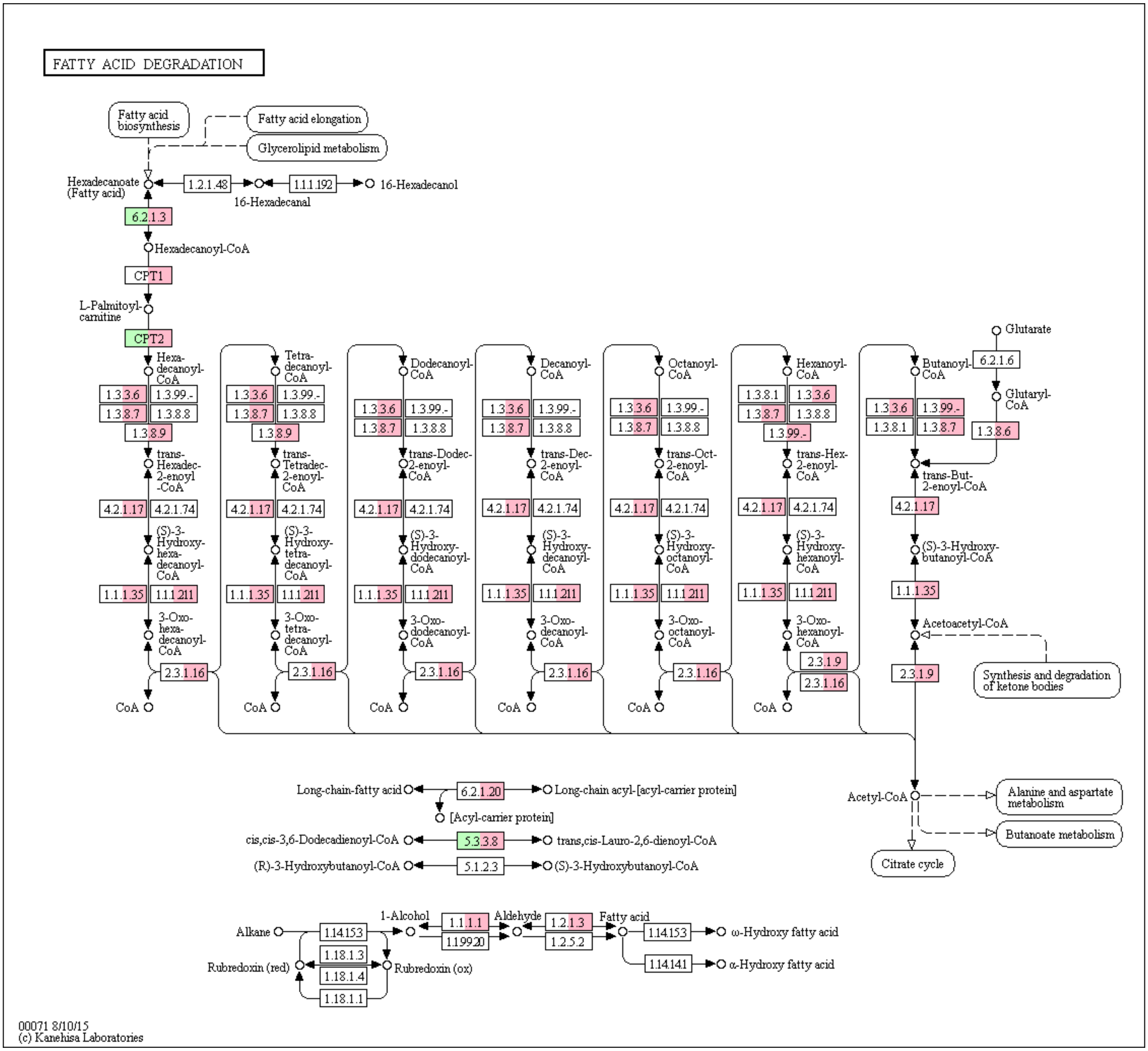

Figure. S3.1.1d. KEGG pathway showing fatty acid metabolism. Proteins with clear homologs in the endosymbiont *Perkinsela*. sp. are highlighted green, while those encoded in the nuclear genome of the host *Paramoeba pemaquidensis* are highlighted red.

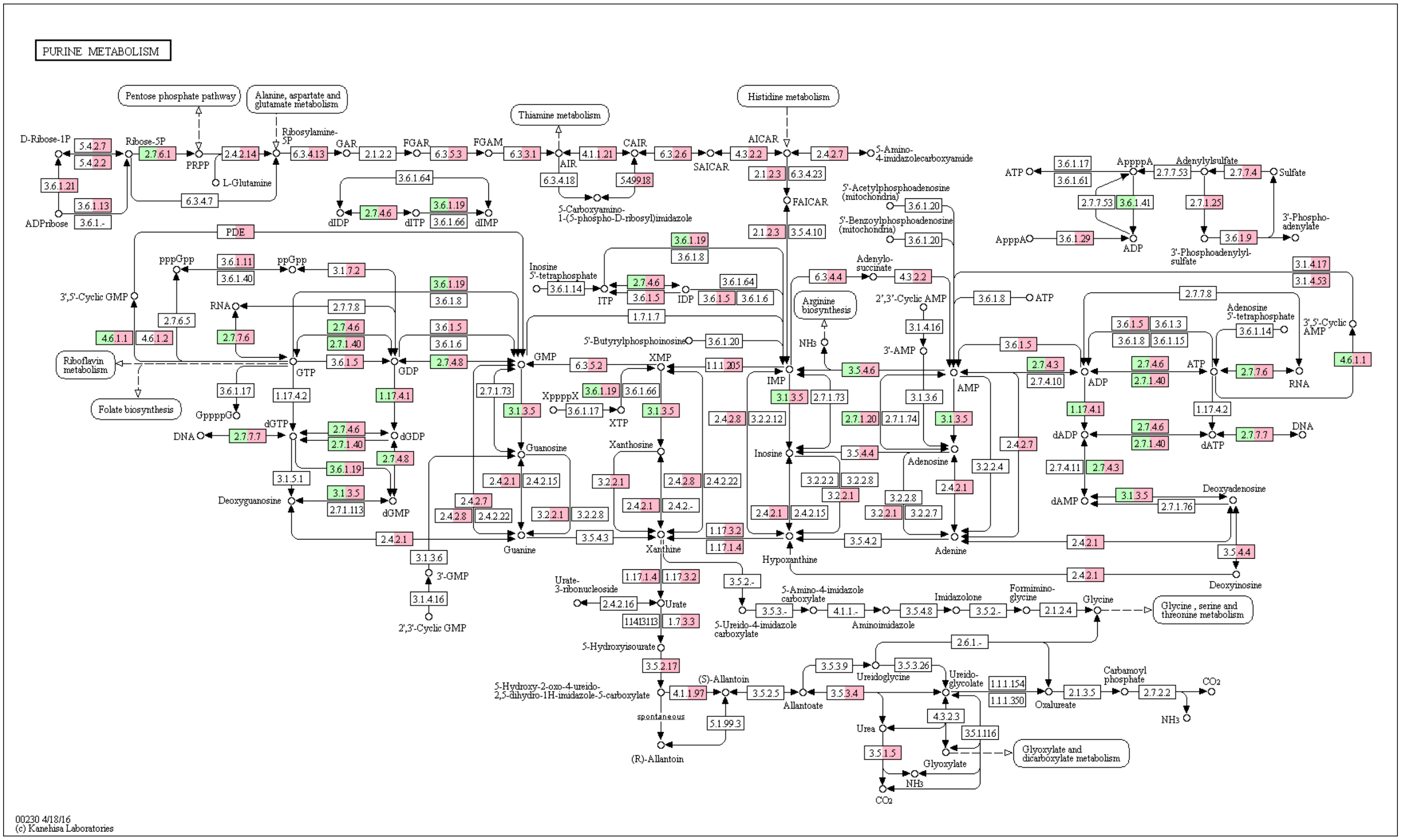

Figure. S3.1.1e. KEGG pathway showing purine metabolism. Proteins with clear homologs in the endosymbiont *Perkinsela*. sp. are highlighted green, while those encoded in the nuclear genome of the host *Paramoeba pemaquidensis* are highlighted red. Green text corresponds to gene / enzyme names in bacteria.

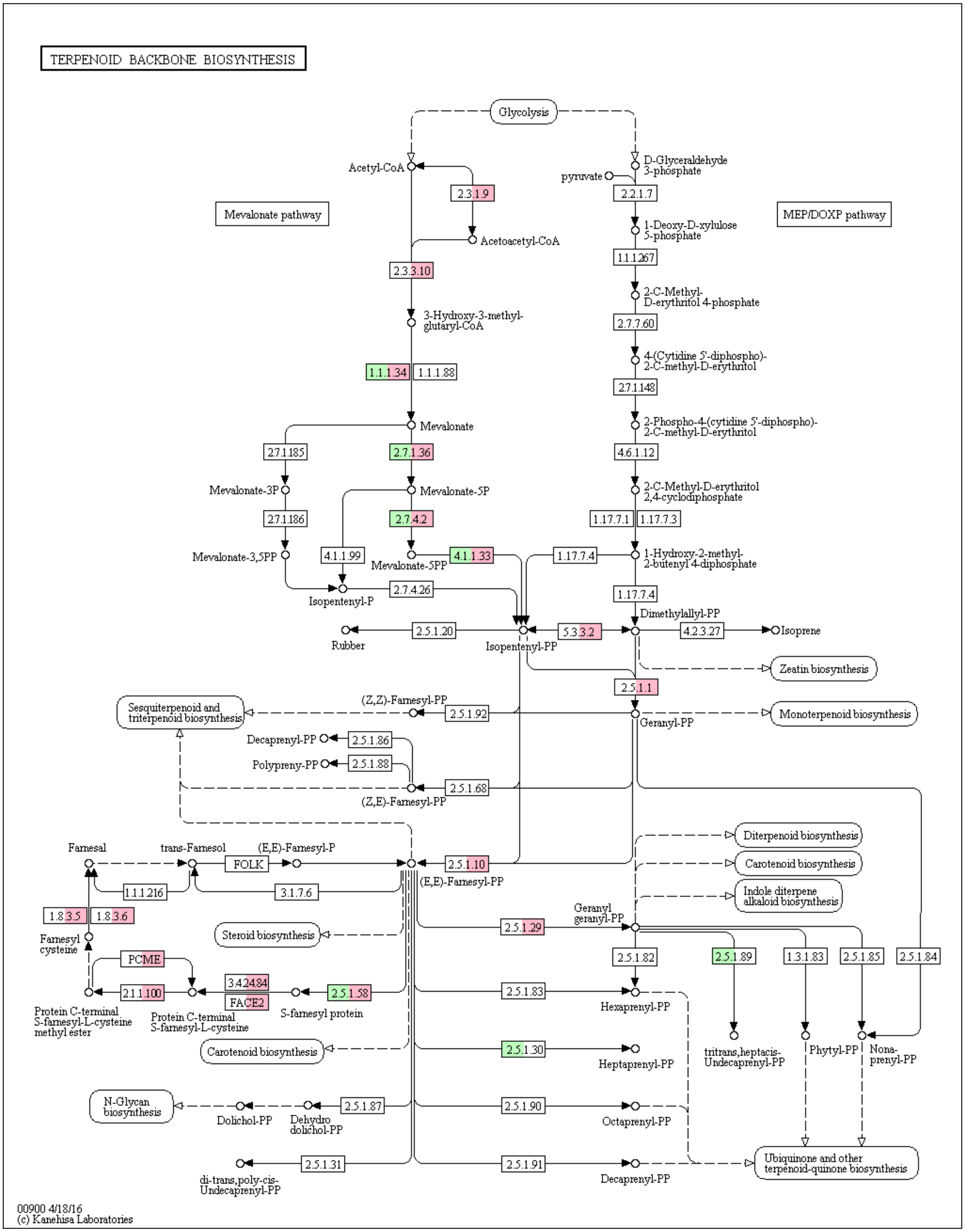

Figure. S3.1.1f. KEGG pathway showing terpenoid biosynthesis. Proteins with clear homologs in the endosymbiont *Perkinsela*. sp. are highlighted green, while those encoded in the nuclear genome of the host *Paramoeba pemaquidensis* are highlighted red.

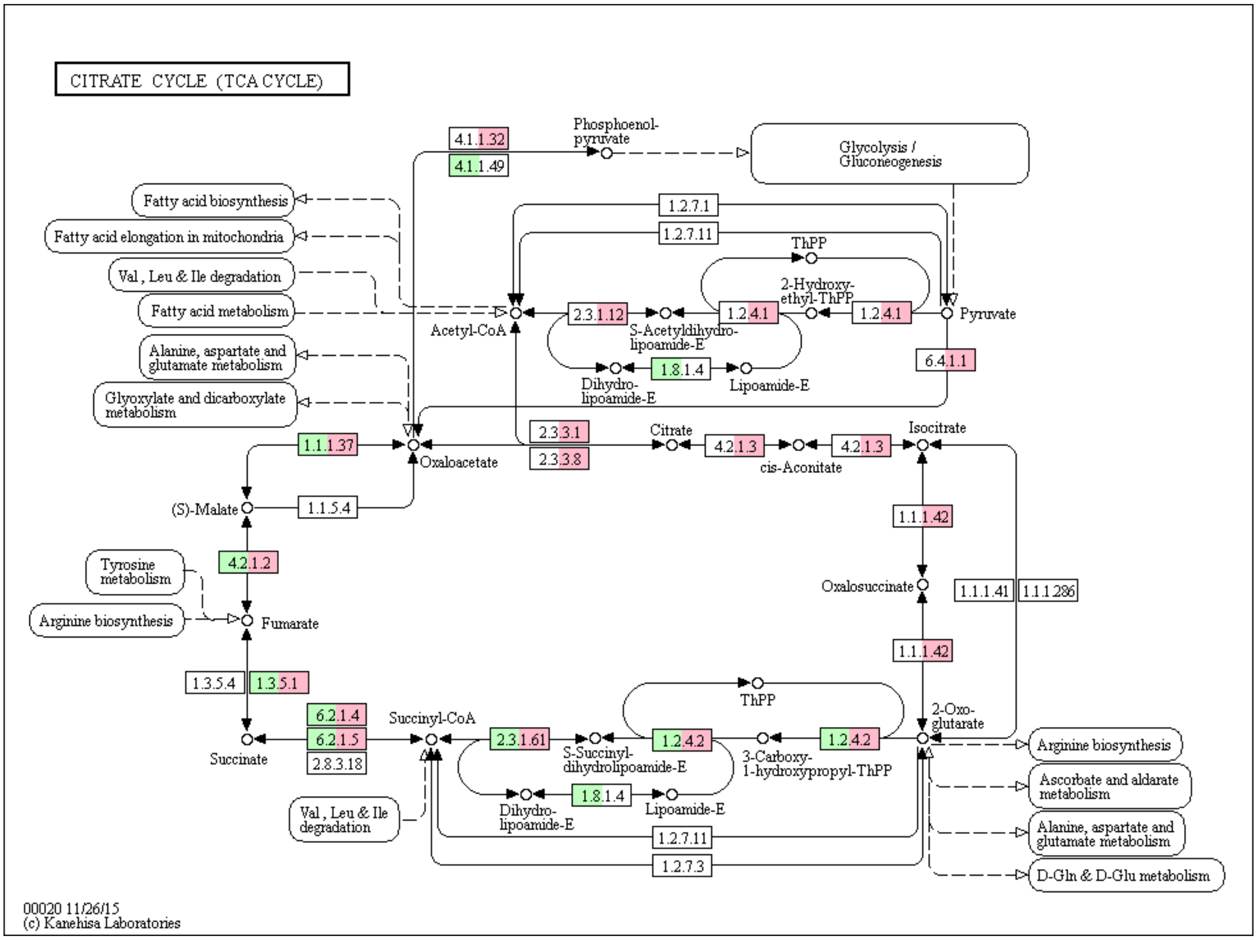

Figure. S3.1.1g. KEGG pathway showing the citrate cycle (tricarboxylic acid cycle). Proteins with clear homologs in the endosymbiont *Perkinsela*. sp. are highlighted green, while those encoded in the nuclear genome of the host *Paramoeba pemaquidensis* are highlighted red.

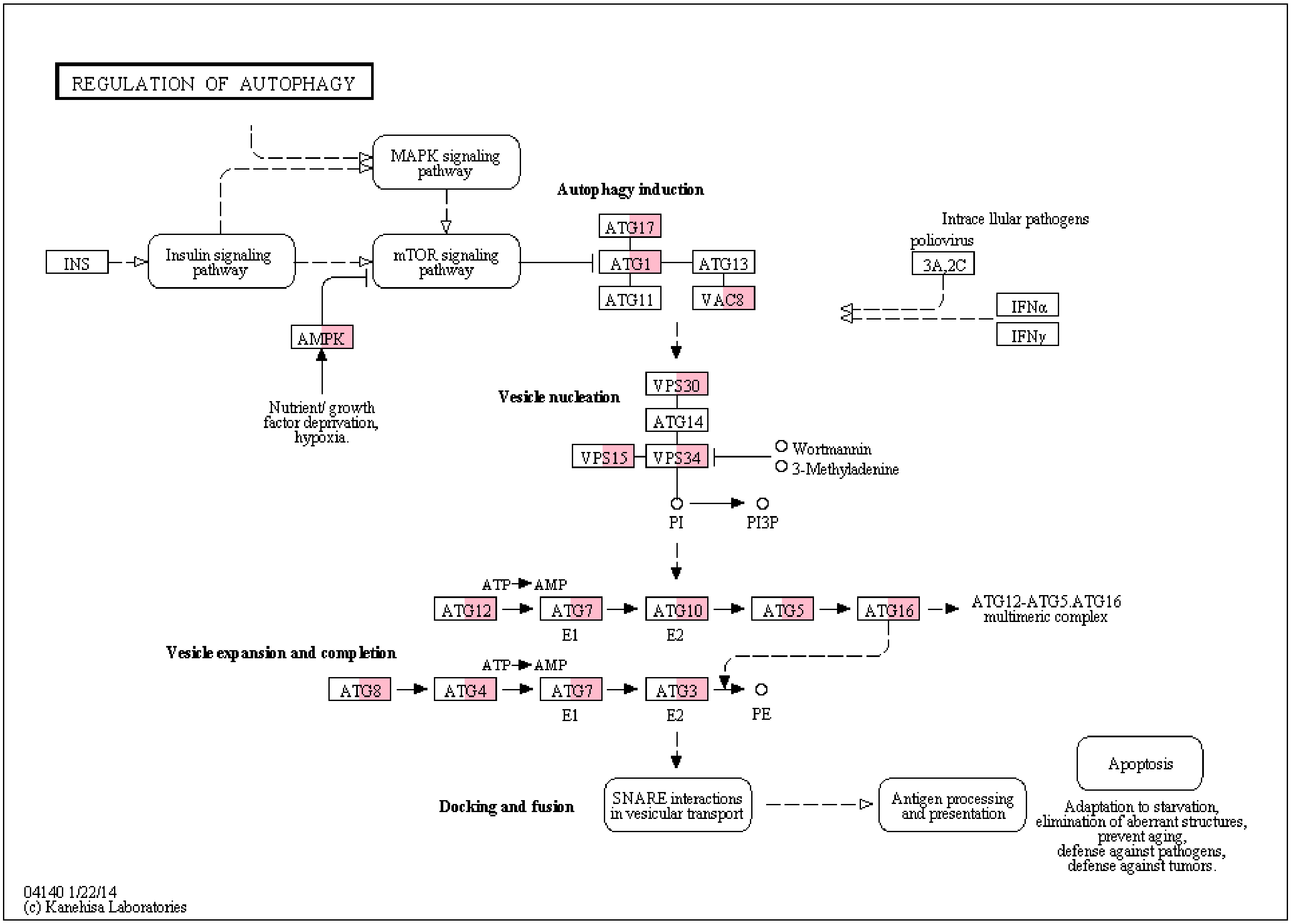

Figure. S3.11.1. KEGG pathway showing autophagy proteins. Proteins with clear homologs identified in the nuclear genome of *Paramoeba pemaquidensis* are highlighted red. No autophagy-associated proteins were identified in the nuclear genome of *Perkinsela*. sp.

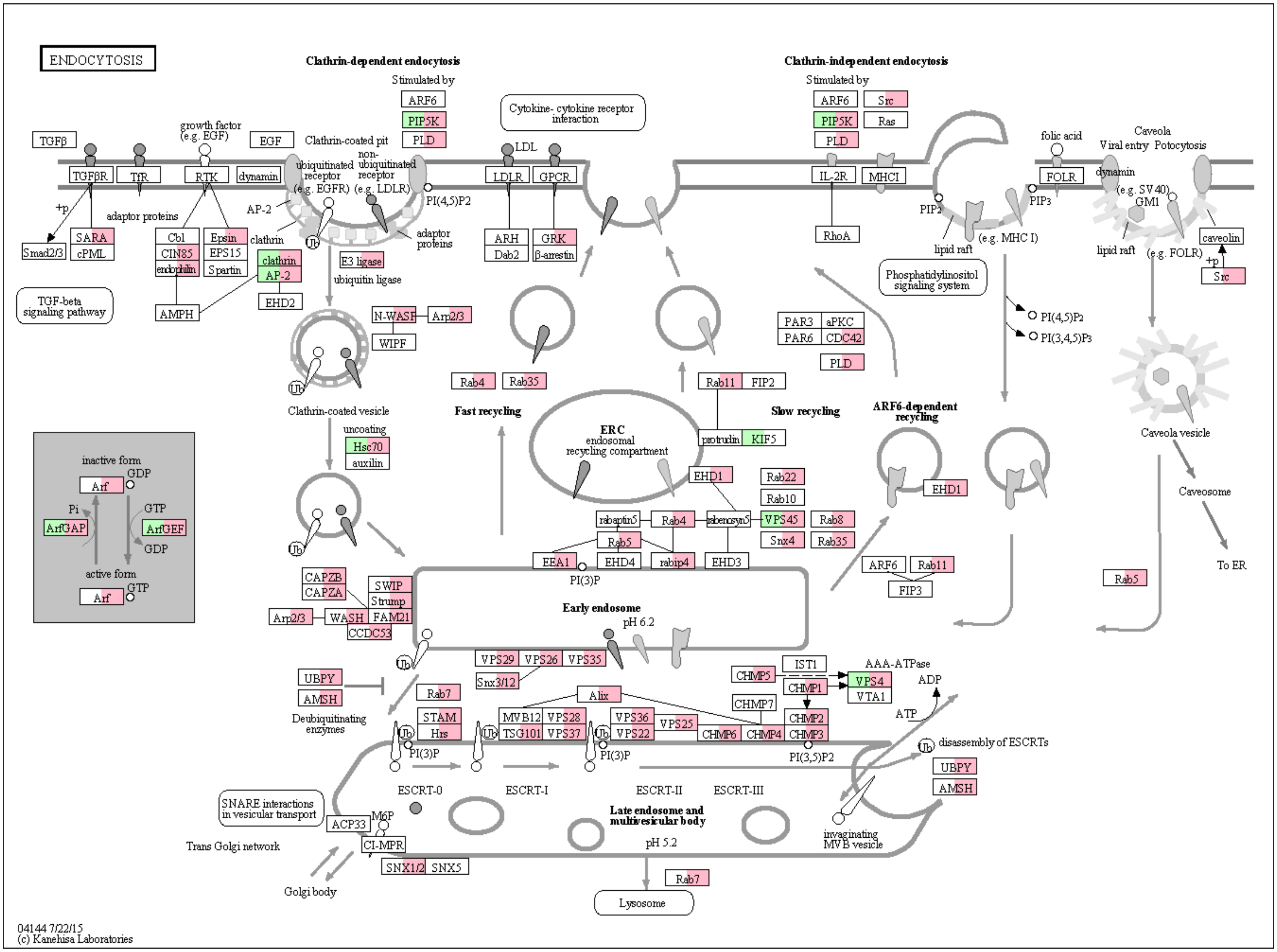

Figure. S3.12.1. KEGG pathway showing endocytosis-associated proteins. Proteins with clear homologs in *Perkinsela*. sp. are highlighted green, while those found in the nuclear genome of *Paramoeba pemaquidensis* are shown in red.

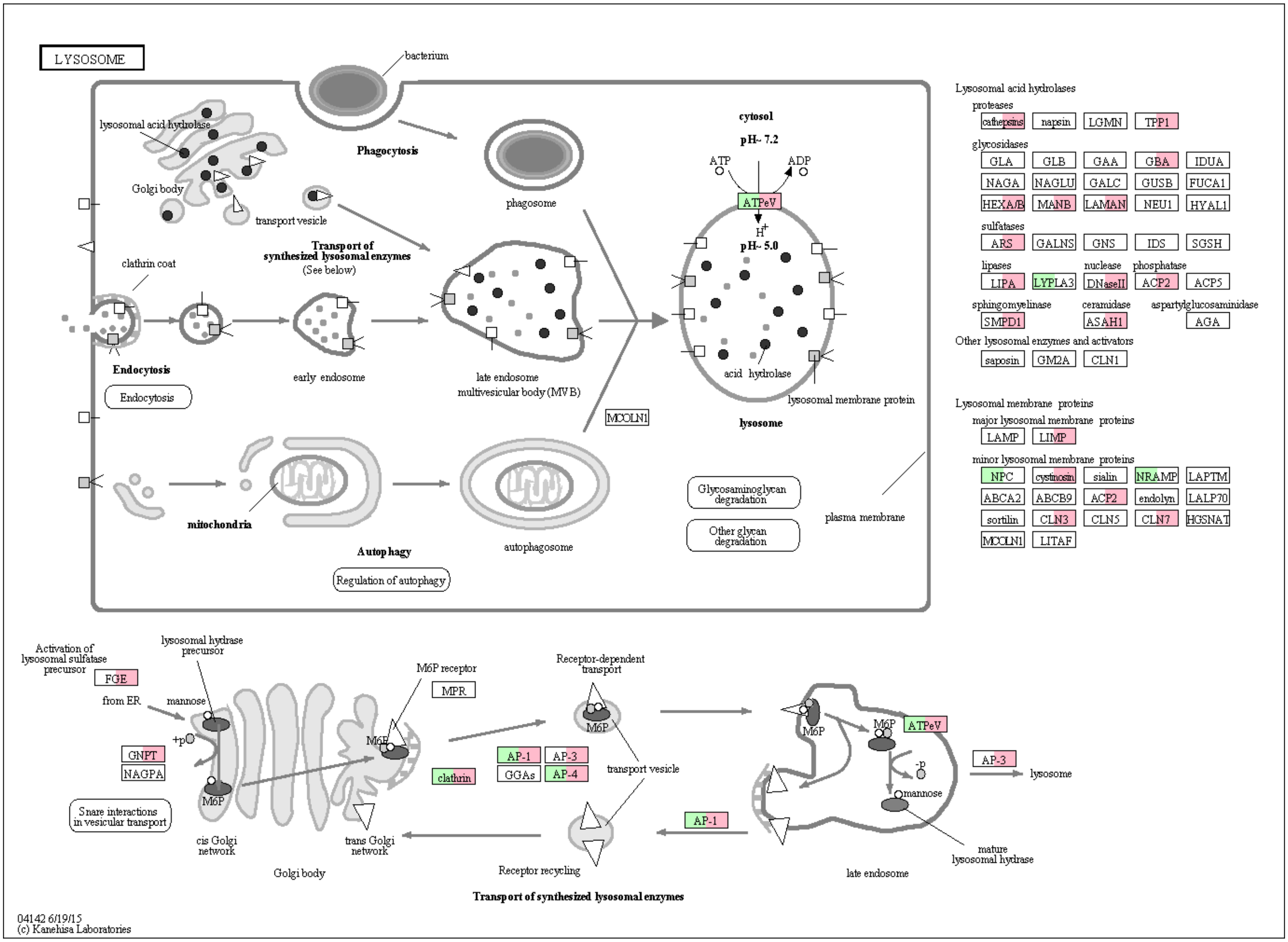

Figure. S3.13.1. KEGG pathway showing lysosome-associated proteins. Proteins with clear homologs in the endosymbiont *Perkinsela*. sp. are highlighted green, while those encoded in the nuclear genome of the host *Paramoeba pemaquidensis* are highlighted red.

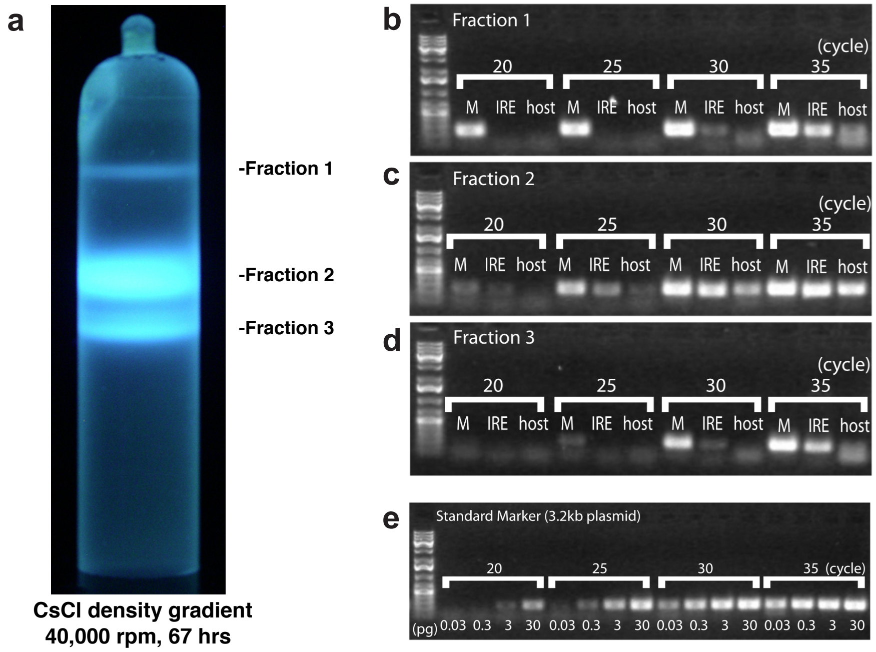

Fig. S4. Density gradient centrifugation and quantification of total DNA fractions isolated from *Paramoeba pemaquidensis* CCAP 1560/4 and its endosymbiont *Perkinsela* sp.

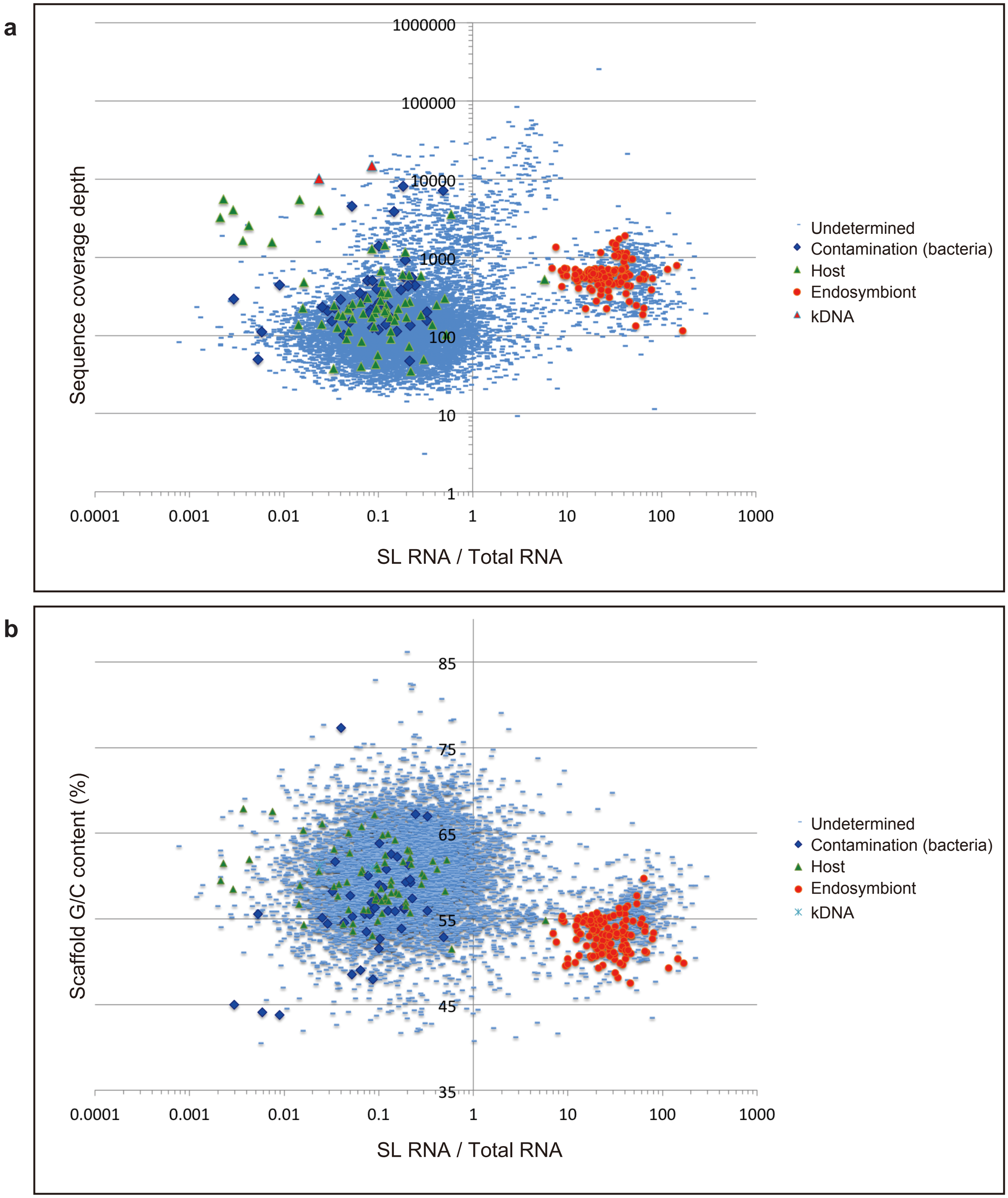

Fig. S5. RNA-seq-based *in silico* profiling of genomic scaffolds from *Paramoeba pemaquidensis* CCAP 1560/4 and its endosymbiont *Perkinsela* sp.

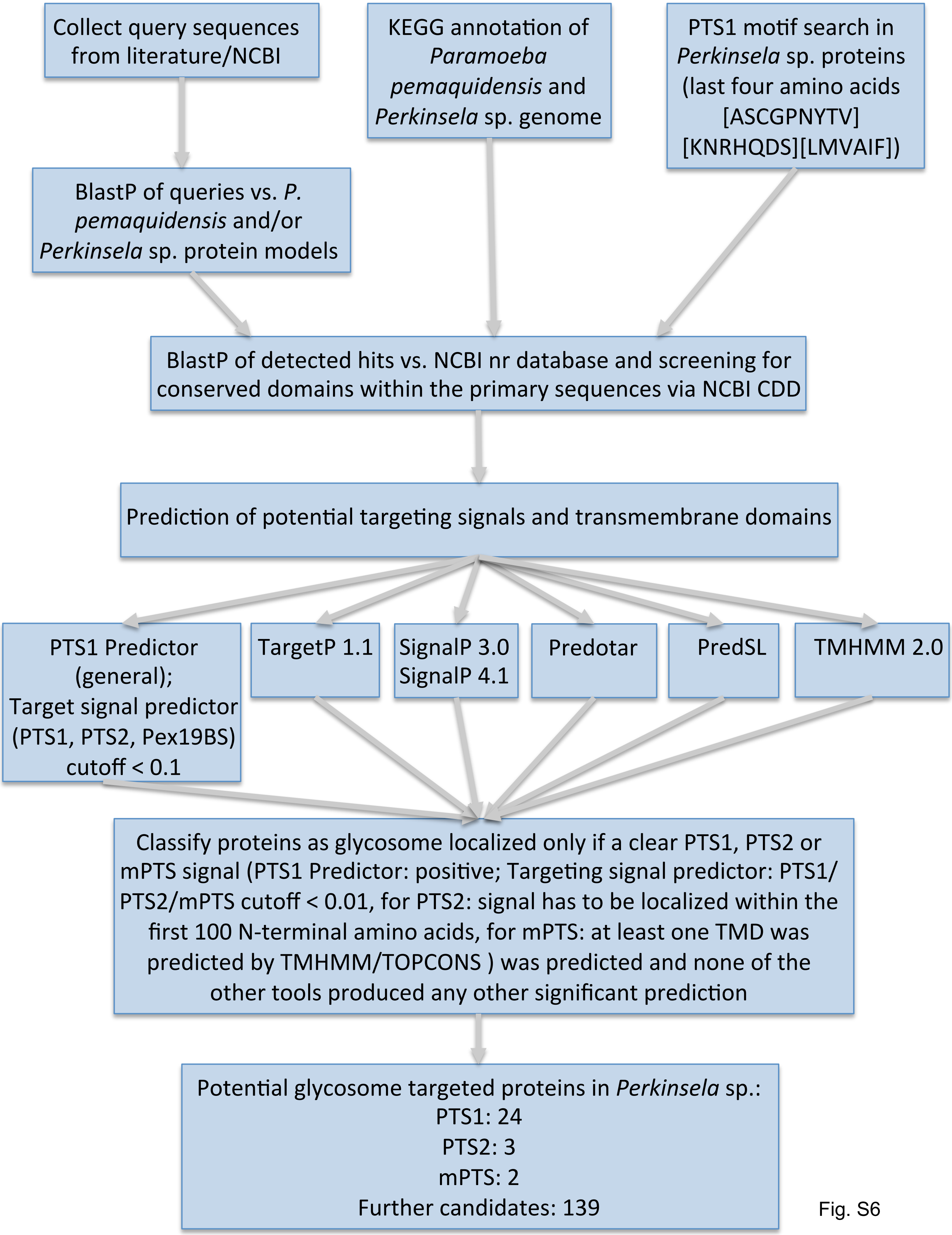
Collect query sequences from literature/NCBI
KEGG annotation of *Paramoeba pemaquidensis* and *Perkinsela* sp. genome
PTS1 motif search in *Perkinsela* sp. proteins (last four amino acids [ASCGPNYTV][KNRHQDS][LMVAIF])
BlastP of queries vs. *P. pemaquidensis* and/or *Perkinsela* sp. protein models
BlastP of detected hits vs. NCBI nr database and screening for conserved domains within the primary sequences via NCBI CDD
Prediction of potential targeting signals and transmembrane domains
PTS1 Predictor (general); Target signal predictor (PTS1, PTS2, Pex19BS) cutoff < 0.1
TargetP 1.1
SignalP 3.0
SignalP 4.1
Predotar
PredSL
TMHMM 2.0
Classify proteins as glycosome localized only if a clear PTS1, PTS2 or mPTS signal (PTS1 Predictor: positive; Targeting signal predictor: PTS1/PTS2/mPTS cutoff < 0.01, for PTS2: signal has to be localized within the first 100 N-terminal amino acids, for mPTS: at least one TMD was predicted by TMHMM/TOPCONS) was predicted and none of the other tools produced any other significant prediction
Potential glycosome targeted proteins in *Perkinsela* sp.:
PTS1: 24
PTS2: 3
mPTS: 2
Further candidates: 139

Fig. S6
