# Supplementary material for: Genome sequencing reveals metabolic and cellular interdependence in an amoeba-kinetoplastid symbiosis
Source: Sci Rep. 2017 Sep 15;7:11688. doi: 10.1038/s41598-017-11866-x (PMC5601477; doi:10.1038/s41598-017-11866-x)
Supplement: Supplementary file 2 — Table S1.1 [file 41598_2017_11866_MOESM2_ESM.doc]

**Genome sequencing reveals metabolic and cellular interdependence in an amoeba-kinetoplastid symbiosis**

Goro Tanifuji^1,2^, Ugo Cenci^1,2^, Daniel Moog^1,2^, Samuel Dean^3^, Takuro Nakayama^4^, Vojtěch David^1,2,5^, Ivan Fiala^5^, Bruce A. Curtis^1,2^, Shannon Sibbald^1,2^, Naoko T. Onodera^1,2^, Morgan Colp^1,2^, Pavel Flegontov^5,6^, Jessica Johnson-MacKinnon^1,2^, Michael McPhee^1,2^, Yuji Inagaki^4,7^, Tetsuo Hashimoto^7^, Steven Kelly^8^, Keith Gull^3^, Julius Lukeš^5,9,10^, and John M. Archibald^1,2,10^

^1^Department of Biochemistry & Molecular Biology, Dalhousie University, Halifax, Nova Scotia, Canada. ^2^Centre for Comparative Genomics and Evolutionary Bioinformatics, Dalhousie University, Halifax, Nova Scotia, Canada. ^3^Sir William Dunn School of Pathology, University of Oxford, Oxford, United Kingdom. ^4^Center for Computational Sciences, University of Tsukuba, Japan. ^5^Institute of Parasitology, Biology Centre, Czech Academy of Sciences, České Budějovice, Czech Republic. ^6^Life Science Research Centre, Faculty of Science, University of Ostrava, Ostrava, Czech Republic. ^7^Graduate School of Life and Environmental Sciences, University of Tsukuba, Japan. ^8^Department of Plant Sciences, University of Oxford, Oxford, United Kingdom. ^9^Faculty of Sciences, University of South Bohemia, České Budějovice, Czech Republic. ^10^Canadian Institute for Advanced Research, CIFAR Program in Integrated Microbial Biodiversity, Toronto, Canada.

Present addresses: Department of Zoology, National Museum of Nature and Science, Tsukuba, Japan (G.T.), Laboratory for Cell Biology, Philipps University Marburg, Germany (D.M.), Graduate School of Life Sciences, Tohoku University, Japan (T.N.), Institute for Marine and Antarctic Sciences, University of Tasmania, Launceston, Australia (J.J-M.), National Institute of Advanced Industrial Science and Technology, Tsukuba, Japan (N.T.O.)

**Table S1.1 Genome and transcriptomic data for *Perkinsela* sp. and *Paramoeba pemaquidensis***

|  | *Perkinsela* sp.^a^ (endosymbiont) | *Paramoeba**pemaquidensis*^a^ (host) |
| --- | --- | --- |
| **Genomic data** |  |  |
| Total [Mbp] | 9.5 | 43.7 |
| # scaffolds | 693 | 9,331 |
| Largest scaffold (Kbp) | 854 | 131 |
| N50 [Kbp] | 57.5 | 8.2 |
| G+C (%) | 47.1 | 43.3 |
| Protein-coding genes | 5,252 | 11,648 |
| Pseudogenes | >500^b^ | n/d |
| Spliceosomal introns | 0 | 40,539 |
| **Transcriptomic data** |  |  |
| Total [Mbp] | 5.4 | 20.5 |
| # contigs | 3,739 | 21,787 |

a. Strain CCAP1560/4; This study.

b. Most identified pseudogenes correspond to retrotransposons and spliced leader RNA genes
