# Supplementary material for: Genome sequencing reveals metabolic and cellular interdependence in an amoeba-kinetoplastid symbiosis
Source: Sci Rep. 2017 Sep 15;7:11688. doi: 10.1038/s41598-017-11866-x (PMC5601477; doi:10.1038/s41598-017-11866-x)
Supplement: Supplementary file 10 — Table S2.5.1 [file 41598_2017_11866_MOESM10_ESM.doc]

**Genome sequencing reveals metabolic and cellular interdependence in an amoeba-kinetoplastid symbiosis**

Goro Tanifuji^1,2^, Ugo Cenci^1,2^, Daniel Moog^1,2^, Samuel Dean^3^, Takuro Nakayama^4^, Vojtěch David^1,2,5^, Ivan Fiala^5^, Bruce A. Curtis^1,2^, Shannon Sibbald^1,2^, Naoko T. Onodera^1,2^, Morgan Colp^1,2^, Pavel Flegontov^5,6^, Jessica Johnson-MacKinnon^1,2^, Michael McPhee^1,2^, Yuji Inagaki^4,7^, Tetsuo Hashimoto^7^, Steven Kelly^8^, Keith Gull^3^, Julius Lukeš^5,9,10^, and John M. Archibald^1,2,10^

^1^Department of Biochemistry & Molecular Biology, Dalhousie University, Halifax, Nova Scotia, Canada. ^2^Centre for Comparative Genomics and Evolutionary Bioinformatics, Dalhousie University, Halifax, Nova Scotia, Canada. ^3^Sir William Dunn School of Pathology, University of Oxford, Oxford, United Kingdom. ^4^Center for Computational Sciences, University of Tsukuba, Japan. ^5^Institute of Parasitology, Biology Centre, Czech Academy of Sciences, České Budějovice, Czech Republic. ^6^Life Science Research Centre, Faculty of Science, University of Ostrava, Ostrava, Czech Republic. ^7^Graduate School of Life and Environmental Sciences, University of Tsukuba, Japan. ^8^Department of Plant Sciences, University of Oxford, Oxford, United Kingdom. ^9^Faculty of Sciences, University of South Bohemia, České Budějovice, Czech Republic. ^10^Canadian Institute for Advanced Research, CIFAR Program in Integrated Microbial Biodiversity, Toronto, Canada.

Present addresses: Department of Zoology, National Museum of Nature and Science, Tsukuba, Japan (G.T.), Laboratory for Cell Biology, Philipps University Marburg, Germany (D.M.), Graduate School of Life Sciences, Tohoku University, Japan (T.N.), Institute for Marine and Antarctic Sciences, University of Tasmania, Launceston, Australia (J.J-M.), National Institute of Advanced Industrial Science and Technology, Tsukuba, Japan (N.T.O.)

**Supplementary Table S2.5.1.** Select peroxin proteins identified in the nuclear genomes of *Perkinsela* sp. and its host, *Paramoeba pemaquidensis*.

| Protein | Function | *Perkinsela* sp. (endosymbiont) | *P. pemaquidensis*  (host) |
| --- | --- | --- | --- |
| Pex1 | ATPase, receptor export | + | + |
| Pex2 | Receptor export (ubiquitination) | + | + |
| Pex3 | PMP receptor docking | nd | + |
| Pex4 | Ubiquitin conjugation (export) | + | nd |
| Pex5 | PTS1 receptor | + | + |
| Pex6 | ATPase, receptor export | + | + |
| Pex7 | PTS2 receptor | + | + |
| Pex10 | Receptor export (ubiquitination) | + | + |
| Pex11 | Peroxisome division | + | + |
| Pex12 | Receptor export (ubiquitination) | + | + |
| Pex13 | Receptor docking | ? | nd |
| Pex14 | Receptor docking | + | + |
| Pex16 | PMP membrane insertion | nd | + |
| Pex19 | PMP receptor | nd | + |

Abbreviations: Pex, peroxin; PTS1/2, peroxisomal targeting signal 1 or 2; PMP peroxisomal membrane protein; +, detected; ?, unclear; nd, not detected.
