# Supplementary material for: Genome sequencing reveals metabolic and cellular interdependence in an amoeba-kinetoplastid symbiosis
Source: Sci Rep. 2017 Sep 15;7:11688. doi: 10.1038/s41598-017-11866-x (PMC5601477; doi:10.1038/s41598-017-11866-x)
Supplement: Supplementary file 13 — Table S2.6.2 [file 41598_2017_11866_MOESM13_ESM.doc]

**Genome sequencing reveals metabolic and cellular interdependence in an amoeba-kinetoplastid symbiosis**

Goro Tanifuji^1,2^, Ugo Cenci^1,2^, Daniel Moog^1,2^, Samuel Dean^3^, Takuro Nakayama^4^, Vojtěch David^1,2,5^, Ivan Fiala^5^, Bruce A. Curtis^1,2^, Shannon Sibbald^1,2^, Naoko T. Onodera^1,2^, Morgan Colp^1,2^, Pavel Flegontov^5,6^, Jessica Johnson-MacKinnon^1,2^, Michael McPhee^1,2^, Yuji Inagaki^4,7^, Tetsuo Hashimoto^7^, Steven Kelly^8^, Keith Gull^3^, Julius Lukeš^5,9,10^, and John M. Archibald^1,2,10^

^1^Department of Biochemistry & Molecular Biology, Dalhousie University, Halifax, Nova Scotia, Canada. ^2^Centre for Comparative Genomics and Evolutionary Bioinformatics, Dalhousie University, Halifax, Nova Scotia, Canada. ^3^Sir William Dunn School of Pathology, University of Oxford, Oxford, United Kingdom. ^4^Center for Computational Sciences, University of Tsukuba, Japan. ^5^Institute of Parasitology, Biology Centre, Czech Academy of Sciences, České Budějovice, Czech Republic. ^6^Life Science Research Centre, Faculty of Science, University of Ostrava, Ostrava, Czech Republic. ^7^Graduate School of Life and Environmental Sciences, University of Tsukuba, Japan. ^8^Department of Plant Sciences, University of Oxford, Oxford, United Kingdom. ^9^Faculty of Sciences, University of South Bohemia, České Budějovice, Czech Republic. ^10^Canadian Institute for Advanced Research, CIFAR Program in Integrated Microbial Biodiversity, Toronto, Canada.

Present addresses: Department of Zoology, National Museum of Nature and Science, Tsukuba, Japan (G.T.), Laboratory for Cell Biology, Philipps University Marburg, Germany (D.M.), Graduate School of Life Sciences, Tohoku University, Japan (T.N.), Institute for Marine and Antarctic Sciences, University of Tasmania, Launceston, Australia (J.J-M.), National Institute of Advanced Industrial Science and Technology, Tsukuba, Japan (N.T.O.)

**Table S2.6.2.** SNAREs and SNARE related proteins in *Perkinsela* sp. and other kinetoplastids.

| **Protein** | ***T. brucei* accession** | ***Perkinsela* sp.?** | ***Bodo saltans*?** |
| --- | --- | --- | --- |
| syntaxin binding protein 1 | Tb927.9.1970 | c47AC | BS70415 |
| QA-SNARE protein putative | Tb927.9.13030 | c1820AC | BS18635 |
| mu-adaptin 4 | Tb927.8.7050 | c1620AC | BS13080 BS57230 |
| delta-COP | Tb927.8.5250 | c1170AC | BS18460 |
| vSNARE | Tb927.8.3470 | c1327AC | BS61715 |
| v-SNARE protein | Tb927.7.6440 | c3823Pgoro_1 | BS92190 BS35105 |
| Mu-adaptin 1 | Tb927.7.3180 | c1620AC | BS13080 BS57230 |
| Synaptobrevin (VAMP) | Tb927.5.3560 | c3097AC c2936AC | BS68915c BS80005 |
| clathrin coat assembly protein ap19 | Tb927.3.4000 | c1232AC | BS80400 BS33010 BS12520 |
| Synaptobrevin | Tb927.2.5120 | c4AC | BS48290 BS91005 |
| signal recognition particle receptor alpha subunit | Tb927.11.9890 | c1919AC | BS23460 |
| t-SNARE | Tb927.11.8790 | c2347AC | BS01515 BS01510 |
| VPS45 | Tb927.10.6780 | c874AC | BS30870 |
| coatomer subunit zeta | Tb927.10.4270 | c2934AC c3095AC | BS34545 |
| Syntaxin | Tb927.10.1830 | c423AC | BS25930 BS81055 |
| Sec22 | Tb927.10.15130 | c5562Pgoro_1 | BS65210 |
| AP-4 complex subunit sigma-1, putative | Tb927.10.14260 | c1232AC | BS80400 BS33010 BS12520 |
| syntaxin 5 | Tb927.10.14200 | c10AC | BS12570 |
| VAMP | Tb927.9.14080 | no | BS88475 |
| BET5 (ER-golgi transport) | Tb927.9.12870 | no | BS18495 |
| clathrin assembly sigma-adaptin protein 3, putative | Tb927.8.6680 | no | BS85090 |
| Synbindin, SNARE superfamily | Tb927.8.5900 | no | BS59770 |
| mu-adaptin 3 | Tb927.4.2020 | no | BS44540 |
| Syntaxin | Tb927.3.5570 | no | BS03490 |
| tSNARE associated golgi protein | Tb927.11.8060 | no | no |
| Synbindin, SNARE superfamily | Tb927.11.15930 | no | BS43095 |
| golgi SNARE protein-like,GOLGI SNAP receptor complex member, putative | Tb927.11.10270 | no | BS74165 |
| tSNARE | Tb927.10.2340 | no | BS24390 |
| SNARE protein | Tb927.10.15500 | no | BS77225 |
| SNARE domain-containing protein, putative | Tb927.10.12450 | no | BS62095 |
| QA-SNARE protein | Tb927.10.11980 | no | BS08940 |
| SNARE associated Golgi protein | Tb927.1.4500 | no | BS00800 |
